# Visible-Light Responsive Sucrose-Containing Macrocyclic Host for Cations

**DOI:** 10.1021/acs.orglett.1c00590

**Published:** 2021-03-17

**Authors:** Patrycja Sokołowska, Kajetan Dąbrowa, Sławomir Jarosz

**Affiliations:** Institute of Organic Chemistry, Polish Academy of Sciences, Kasprzaka 44/52, 01-224 Warsaw, Poland

## Abstract

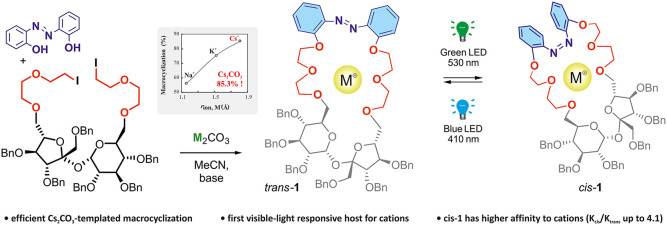

Chiral photoresponsive host **1** was prepared by a high-yield Cs_2_CO_3_-templated macrocyclization. *Trans*-**1** transforms into long-lived *cis*-**1** (25 days) upon irradiation with green light, and the backward transformation is triggered by blue light. Both isomers prefer potassium among alkali metal cations, and *cis*-**1** binds cations stronger than *trans*-**1** (*K_cis_*/*K_trans_* ≤ 4.1). ^1^H NMR titration experiments as well as density functional theory studies reveal that sucrose ring oxygen residues and azobenzene nitrogen atoms in **1** contribute to cation coordination.

One of the most investigated areas in supramolecular chemistry concerns the development of macrocyclic systems that can bind neutral or charged molecules.^1^ In principle, the binding properties of such host–guest arrangements could be adjusted toward a specific guest by changing the geometry of the binding pocket as well as the type and number of binding sites attached to the macrocyclic skeleton. For example, incorporation of amino acids, binaphthyls, and sugars introduces chirality into the binding pocket, allowing discrimination of chiral guests. Carbohydrates are particularly attractive among chiral building blocks due to their availability and diversity of structures. On top of that, insertion of the photochromic moiety allows one to change the shape and geometry of the macrocyclic framework in a dynamic fashion using light as a stimulus. From a variety of available chromophores, azobenzene is particularly attractive due to its robustness, excellent photochromic properties, and established chemistry.^2^ The latter feature greatly facilitates the introduction of this scaffold into a desired location in the host platform. Recently, a variety of macrocyclic compounds bearing the azobenzene unit have been reported.^3^ They found broad applications as host–guest systems,^4^ as molecular machines,^5^ or in self-organization processes.^6^ Despite that, only a limited number of macrocyclic photoresponsive host–guest systems incorporating chiral motifs have been reported.^7^ Moreover, light-controlled chiral recognition was to date reported for only phosphates^8^ and carboxylates^9^ using nonmacrocyclic hosts. One of the main research topics of our team is the development of sucrose-derived macrocyclic systems, including, among others, cation^10a,10b^ and anion receptors,^10c,10d^ cryptands,^10e^ and molecular containers.^10f^ On the contrary, we are involved in the development of light-controlled nonmacrocyclic receptors for anions.^9,11^

Herein, we synthesized the first dynamic chiral macrocyclic host **1** that responds reversibly to visible light and shows enhanced binding affinity toward achiral and chiral cations in the metastable *cis* state. Preparation of host **1** was accomplished in six steps starting from sucrose (Scheme 1 and Table 1).

**Scheme 1:**
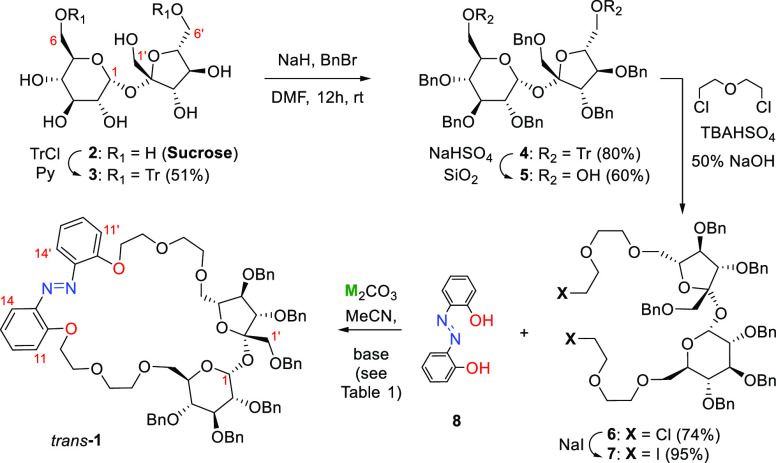
Synthesis of Photoresponsive Chiral Host **1**

**Table 1:** Optimization of the Base-Promoted Macrocyclization between **7** and **8**^a^

entry	base	*T* (°C)	*t* (h)	yield (%)^b^
1	Na_2_CO_3_	50	72	0
2		82	48	56.0
3	K_2_CO_3_	50	48	65.0
4		82	8	75.4
5	Cs_2_CO_2_	50	48	81.5
6		82	6	85.3 (37.0)^c^
7	TBA_2_CO_3_	82	24	trace^d^
8	TBAOMe	82	24	trace^d^

a Reaction conditions: anhydrous MeCN (20 mM, 1:1 **7**:**8** molar ratio), 8 equiv of M_2_CO_3_.

b Total yield for *trans*- and *cis*-**1**.

c With 14 equiv of TBACl added.

d Full consumption of starting materials.

Selective protection of the 6- and 6′-hydroxyl groups of sucrose by trityl block provided 6,6′-di-*O*-tritylsucrose (**3**) in 51% yield. The remaining free hydroxyl groups were then protected as benzyl ethers under standard conditions to afford fully protected sucrose **4** in very good yield (80%). The trityl groups were then selectively removed using heterogeneous silica-supported sodium hydrogen sulfate (NaHSO_4_/SiO_2_),^12^ which provided diol **5** in 60% yield. Hexa-*O*-benzyl sucrose **5**(13) was then reacted with an excess of bis(2-chloroethyl)ether under PTC conditions to provide bis-chloro derivative **6** in 74% yield. To facilitate the subsequent cyclization reaction, the chlorine atoms were replaced with iodine atoms, which afforded bis-iodo polyethyl ether **7** in 96% yield. Finally, key intermediate **7** was subjected to the reaction with 2,2′-dihydroxyazobenzene (**8**) to afford the target 29-member macrocycle **1** in very high yield [85.3% under optimized conditions, which included 6 h and 8 equiv of Cs_2_CO_3_ in refluxing MeCN (see Table 1)]. The reaction virtually does not occur at rt for alkali metal carbonates or even at 50 °C for Na_2_CO_3_ (Table 1, entry 1).

At reflux, the yields of the cyclization are clearly correlated with the size of the alkali metal cation^13^ (see the inset in Scheme 2). The smallest sodium (*r*_ion_ = 1.16 Å), intermediate potassium (*r*_ion_ = 1.52 Å), and the largest cesium (*r*_ion_ = 1.81 Å) provide macrocyclic host **1** in 56%, 75.4%, and 85.3% yields, respectively (entries 2, 4, and 6, respectively). Cs_2_CO_3_ was also effective at 50 °C, providing the cyclic product in 81.5% yield after 48 h (entry 5), whereas K_2_CO_3_ was much less effective at a lower temperature (entry 3). In striking contrast, tetrabutylammonium (TBA) salts of carbonate and methanolate (TBA_2_CO_3_ and TBAOMe, entries 7 and 8, respectively), despite the full consumption of substrate **8**, provide only traces of target macrocyclic host **1** along with unidentified polar products.^14^ Furthermore, addition of an excess of TBACl to Cs_2_CO_3_ markedly decreases the yield of **1** due to the cation-metathesis effect (entry 6 in parentheses). All of these observations indicate that the bulky TBA cation lacks the templating ability.

**Scheme 2:**
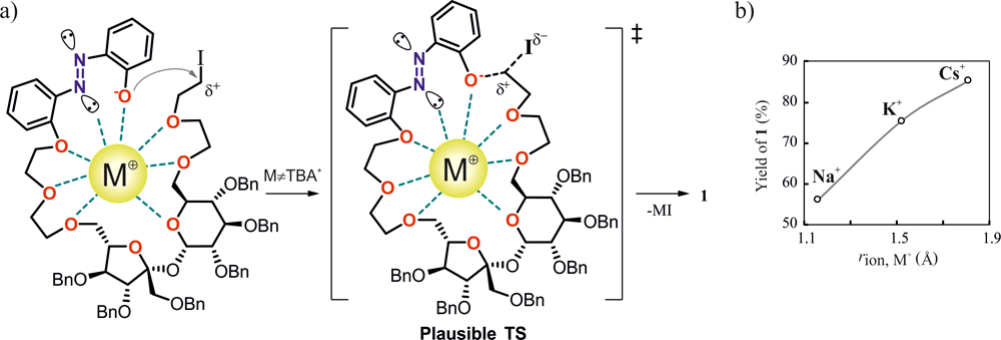
(a) Proposed Mechanism of Alkali Metal Cation-Induced Stabilization of Intermediate Acyclic Polyether Leading to **1** and (b) Dependence of Reaction Yield on the Size of the Cation^13^

The efficient and clean formation of **1** with a highly crowded hexa-*O*-benzyl-sucrose scaffold using alkali metal carbonates suggests the preorganized looplike structure of the linear intermediate that is formed after the first O-alkylation step (Scheme 2). The preorganization facilitates the key ring-closing step over the oligomerization and is probably driven by the size-selective coordination of the alkali metal cation by multiple O and N donor atoms (Scheme 2b).

Recently, we noticed a similar guest templation effect in the synthesis of *C*_2_-symmetric sucrose ureas.^10c^ The presence of chloride anions leads to macrocyclic products in 90% yield, whereas their absence results in a nearly 2-fold decrease in yield (40%). Likewise, the addition of potassium iodine allows the preparation of aza macrocyclic sucroses in good to excellent yields (67–96%).^15^

Nonetheless, the efficient preparation of sucrose-containing macrocycles such as *trans*-**1** is rather surprising, because generally macrocyclic azobenzenes containing sugar or polyoxoethylene moieties are prepared in much lower yields (as shown in Figure 1). For example, the 17-member macrocyclic host with a phenyl thio-α-d-mannopyranoside scaffold, reported recently by the Xie group,^7a,7b,7d^ was prepared in low 35% yield along with the nonmacrocyclic disubstituted byproduct (32%) using a K_2_CO_3_/18-crown-6 system in acetone (Figure 1a). A particularly striking example is the preparation of simple azobenzene crown ethers structurally related to **1** reported by Shinkai et al.^16^ (Figure 1b) and Shiga et al.^17^ (Figure 1c). The azobenzo-crown platform was further extended to a set of nitrogen analogues (Figure 1d) by Luboch and Wagner-Wysiecka,^18^ yet the yields of the *t*-BuOK-mediated macrocyclization were still low to moderate (23–55%). These examples clearly highlight the potential advantage of the utilization of a sucrose scaffold in the construction of cation-templated synthesis of macrocyclic host–guest systems.

**Figure 1:**
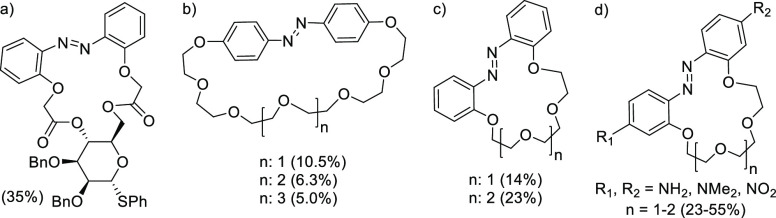
Structures and macrocyclization yield (in parentheses) of reported systems containing an azobenzene switch.^7a,7b,7d,17−19^

Having the macrocyclic host *trans*-**1** in hand, we investigated its photoswitching properties in MeCN (Scheme 3).

**Scheme 3:**
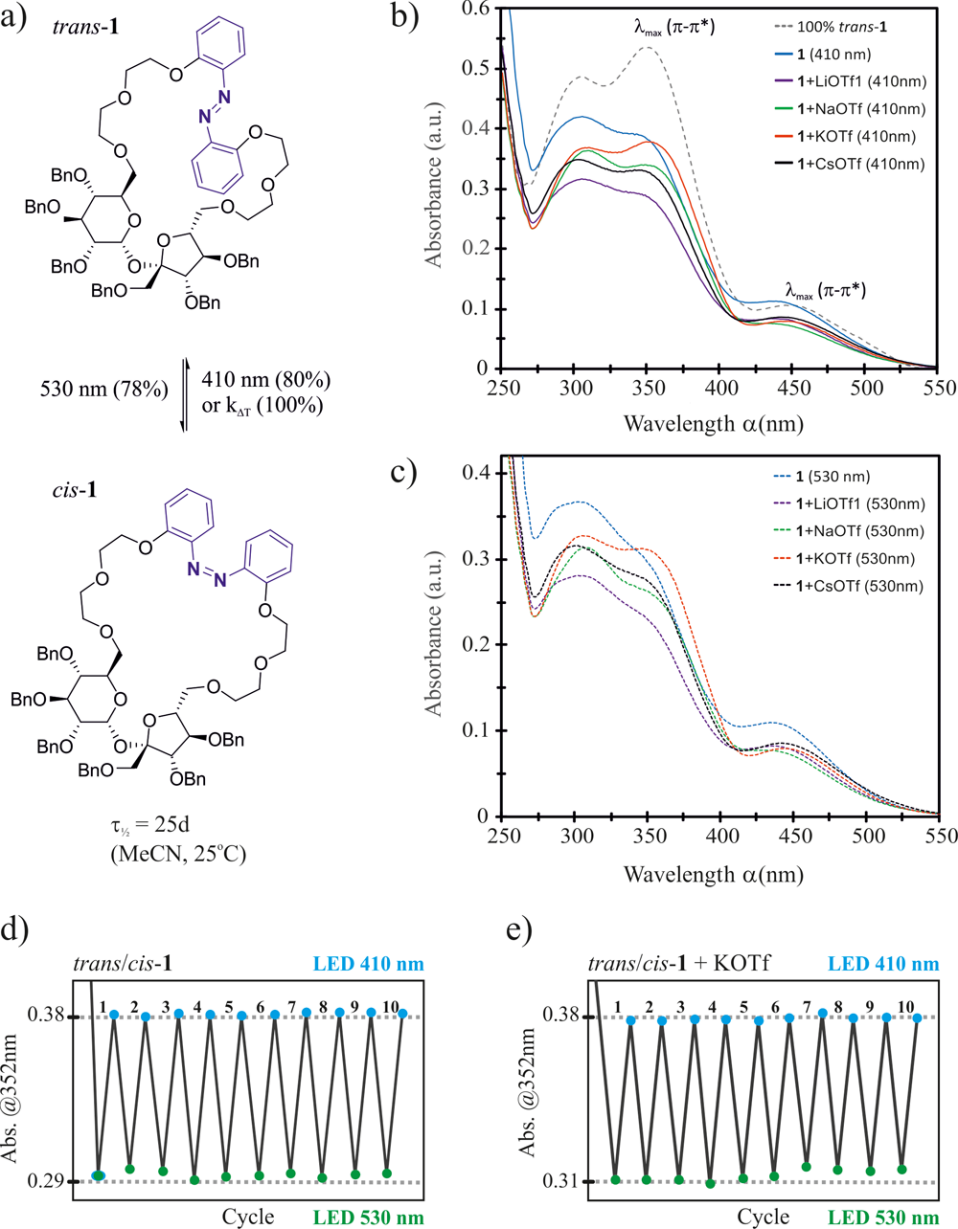
Photoswitching of Host **1** (a–c and d) and Complexes of **1** with 10 equiv of Alkali Metal Triflates (b, c, and e)^[a]^ in MeCN (50 μM) at 298.0 ± 0.1 K Using Green Light (LED 530 nm) and Blue Light (LED 410 nm), Respectively

The absorption spectrum of *trans*-**1** in MeCN shows three maxima (λ) at 308, 350, and 446 nm (Scheme 3b). The latter, relatively intense band is located in the visible region and corresponds to the n−π* transition of the azobenzene chromophore. We envisioned that irradiation of *trans*-**1** with green light (495–570 nm) will allow the *trans* → *cis* isomerization to occur, due to the blue-shifted absorption of the *cis* isomer in this region. *trans*-**1** readily undergoes photoisomerization to produce almost 78% of *cis*-**1** upon irradiation with monochromatic green light at 530 nm [3 W light-emitting diode (LED)] (Scheme 3a). The reverse *cis* → *trans* isomerization using blue light at 410 nm (3 W LED) produced an 80% fraction of *trans*-**1** at PSS_410_.

To the best of our knowledge, this is a first example of the photoresponsive host for cations that can undergo the *trans* ↔ *cis* isomerization without employing UVA light (315–400 nm).

Furthermore, hosts **1** (*cis* or *trans*, Scheme 3b–d) as well as complexes of **1** with alkali metal triflates (Scheme 3b,c,e and Figures S2–S7) showed no signs of photodegradation after several cycles of alternate irradiation with green and blue light. This is in marked contrast to the azobenzene crown ethers reported by Schiga,^17b^ which decompose during photoisomerization (Figure 1c). Further investigation by UV–vis revealed that the *trans*/*cis* ratio of **1** at PSS_530_ is affected by the type and concentration of the added alkali metal triflate, which is attributed to the cation-induced shift of absorption maxima of both isomers (Figures S3–S7). Furthermore, host *cis*-**1** was found to be remarkably stable showing the *t*_1/2_ of 596 h (24.9 days) in MeCN at 298 K. For comparison, the structurally related *cis*-tetra-*o*-OMe-azobenzene derivative reported by the Wooley group^19^ is considerably less stable (*t*_1/2_ ≈ 2–14 days), which suggests that a macrocyclic effect is responsible for the observed high thermal stability of *cis*-**1**. The rotational restrictions, forced by the macrocyclic ring strain, raise the barriers of the thermal activation energy (*E*_a_) and Gibbs free energy (Δ*G*°) to 110.5 and 110.0 kJ mol^–1^, respectively.

To evaluate the solution binding properties of *trans*-**1** and *cis*-**1**, we performed ^1^H NMR titration measurements in CD_3_CN using noncoordinating and highly soluble triflate salts of alkali metal cations (Li^+^, Na^+^, K^+^, and Cs^+^). The corresponding association constants (*K*_a_’s) are listed in Table 2. Both *trans*-**1** and *cis*-**1** show a preference for K^+^ among all alkali cations (except LiOTf), like dibenzo-18-crown-6. *cis*-**1** binds cations 2.1–4.1 times stronger than *trans*-**1** and shows the highest affinity for KOTf (*K*_a_ = 2776 M^–1^), whereas triflates of Li^+^, Na^+^, and Cs^+^ are bound 79.3, 6.2, and 7.5 times weaker, respectively.

**Table 2:** Stability Constants *K*_a_ (M^–1^) for Complexes of Hosts *trans*-**1** and *cis*-**1** with Alkali Metal Cations^a^

cation	*r*_ion_^b^	*trans*-**1**^c^	*cis*-**1**^d^	*K*_*cis*_/*K*_*trans*_
Li^+^	0.90	37	21	0.57
Na^+^	1.16	210	446	2.12
K^+^	1.52	1023	2776	2.71
Cs^+^	1.81	90	370	4.10

a Determined in MeCN-*d*_3_ by ^1^H NMR titrations at 303 K and assuming a 1:1 binding model; HypNMR 2008 for nonlinear curve fitting; anions added as triflate (CF_3_SO_3_^–^) salts; estimated errors of ±10%.

b Taken from ref (13).

c Titration was carried out in the dark using pure *trans*-**1** (see the Supporting Information for details).

d Titration was carried for the *cis*-enriched mixture (see the Supporting Information for details).

*trans*-**1** binds KOTf with a considerably lower affinity (*K*_a_ = 1023 M^–1^), rendering high *cis*/*trans* selectivity (*K*_*cis*_/*K*_*trans*_ = 2.71). An even more pronounced light-controllable change in selectivity is observed for CsOTf (*K*_*cis*_/*K*_*trans*_ = 4.10). Furthermore, *trans*-**1** shows lower binding selectivity than *cis*-**1**; LiOTf, NaOTf, and CsOTf are bound 27.6, 4.9, and 9.1 times weaker than KOTf, respectively. The cation binding selectivity of *trans*-**1** and *cis*-**1** is reflected in the trend and magnitude of ^1^H NMR chemical shift changes observed upon addition of triflate salts (Figure 2 and Figures S3–S26).

**Figure 2:**
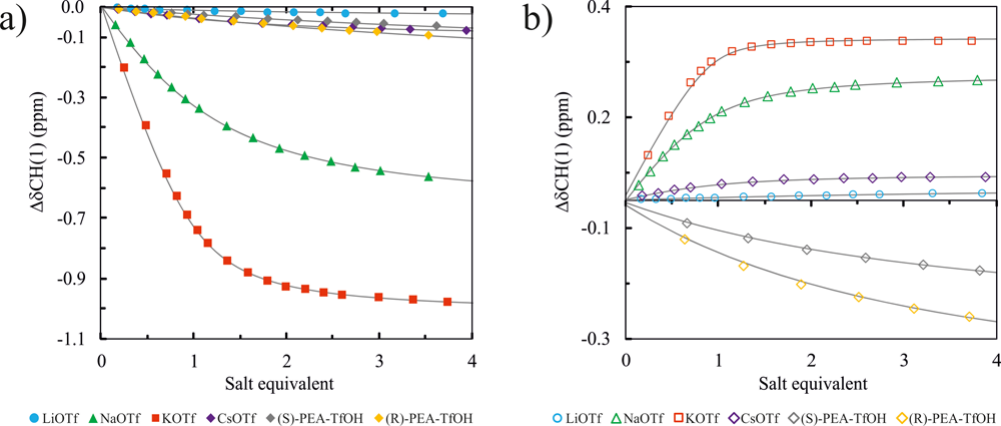
Comparison of the chemical shift changes (Δδ) for anomeric proton CH(1) upon addition of cation triflates to the solution of hosts (a) *trans*-**1** and (b) *cis*-**1** in CD_3_CN. Fitted binding isotherms (gray lines). For the proton label, see Scheme 1.

Analysis of the ^1^H NMR spectra reveals that the addition of both the smallest LiOTf and the largest CsOTf salts has a marginal effect on the chemical shifts of both isomers, suggesting that the binding of the cation is realized outside the macrocyclic cavity, most likely near azobenzene scaffold (see the Supporting Information for details). Interestingly, the *trans* and *cis* isomers exhibit contrasting changes in chemical shifts of the α-glucose anomeric proton CH(1). Namely, near 1.0 ppm upfield and 0.32 ppm downfield a shift of the CH(1) hydrogen signal is observed after the addition of 4 equiv of KOTf to the solutions of *trans*-**1** and *cis*-**1**. At the same time, the aromatic CH resonances of the azobenzene moiety were shifted downfield by ≤0.55 and ≤0.28 ppm for the *trans* and *cis* isomers respectively, exemplifying the indirect involvement in cation binding. Among other factors, the shift behavior of the CH(1) resonance results from (i) the cation-induced conformational change of the binding pocket, (ii) the deshielding effect of the cationic guest localized in the proximity, and (iii) geometry and distance-dependent through-space effects associated with the ring currents of benzyl moieties. For the *cis* isomer, effect (ii) has a decisive impact on the proton shift, whereas for the *trans* isomer, effects (i) and (iii) prevail. On top of that, careful analysis of the ^1^H NMR titration spectra reveals that *cis*-**1** displays a distinct binding behavior for KOTf versus NaOTf. In particular, the CH_sp2_(11) and CH_sp2_(11′) protons (for numbering, see Scheme 1) exhibit upfield and downfield shifts upon addition of NaOTf and KOTf salts, respectively (Figures S23–S26). The plausible explanation is that the larger K^+^ cation can coordinate with lone pairs of the N=N linkage, whereas the smaller Na^+^ interacts with these donor groups to a lesser extent. Furthermore, a specific through-space interaction between *O*-benzyl residues and the azobenzene scaffold might also be responsible for this effect. As depicted in Figure S8, a clear dependence of the stability constants (log *K*_a_) for *trans*-**1** and *cis*-**1** on the alkali cation radius suggests that both isomers possess cavities of similar sizes. This indicates that the increased cation affinity of *cis*-**1** presumably results from the higher number of hydrogen bond interactions and/or a more effective spatial arrangement of hydrogen bond donor atoms. To gain better insights into the binding mode of *trans*-**1** and *cis*-**1** toward alkali metal cations in solution, we performed DFT calculations using B3LYP-D3 combined with the 6-31G(d) basis set and C-PCM model (MeCN; ε = 37.5) to approximate the solvent effects. The energy-minimized conformations of the complexes of *trans*-**1** and *cis*-**1** with K^+^ are demonstrated in Figure 3. The results of DFT calculations are in line with the experimental data showing that *cis*-**1** binds K^+^ stronger than *trans*-**1** by 4 kJ mol^–1^ (see Table S3). For both isomers, the potassium cation is encapsulated in a three-dimensional cryptandlike cage by multiple interactions with the O-donors and lone pairs of the N=N bond.

**Figure 3:**
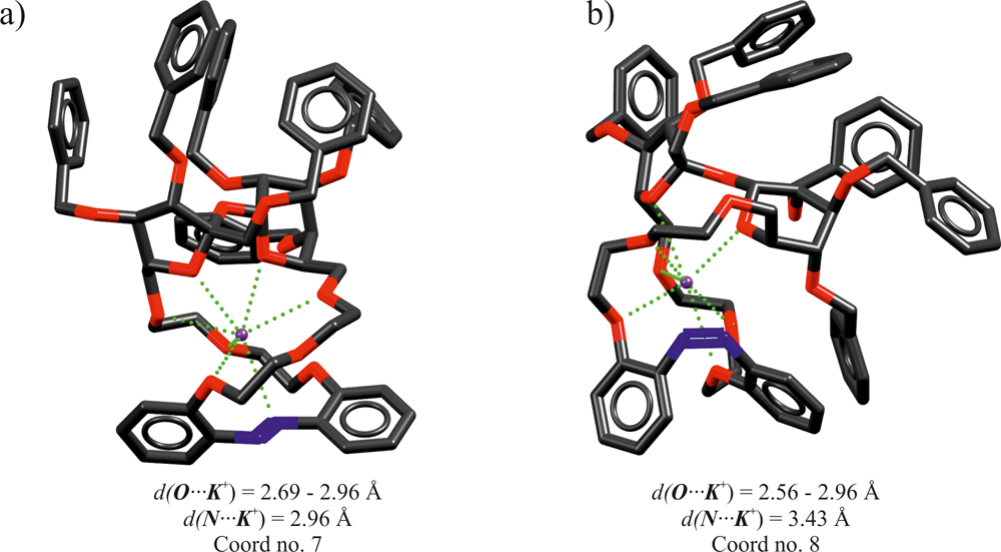
Models of the energy-minimized host–guest complexes (a) [K⊂*trans*-**1**]^+^ and (b) [K⊂*cis*-**1**]^+^, where ⊂ denotes encapsulation.

*cis*-**1** displays a larger number of interactions with potassium (coordination number of 8) compared to *trans*-**1** (coordination number 7). In addition, *cis*-**1** exhibits a particularly short K^+^···O bond length (*d* = 2.56 Å) originating from the glucose ring oxygen atom, which might greatly contribute to stabilization of the complex.

Furthermore, to elucidate the chiral recognition properties of geometrical isomers of **1**, we also tested triflates of *R* and *S* enantiomers of the 2-phenylethylammonium (PEA) cation. These chiral guests were bound with considerably lower affinity than the achiral ones by both *trans*-**1** (*K*_a_ = 19 and 22 M^–1^ for the *R* and *S* enantiomers, respectively) and *cis*-**1** (*K*_a_ = 38 and 42 M^–1^ for the *R* and *S* enantiomers, respectively) hosts, plausibly due to their large size and more dispersed charge of the primary ammonium cation. The discrimination of the PEA enantiomers by *trans*-**1** (*K*_*R*_/*K*_*S*_ = 1.15) and *cis*-**1** (*K*_*R*_/*K*_*S*_ = 1.11) is relatively weak, yet the *cis* isomer exhibits near 2 times better affinity for (*R*)-PEA (*K*_*cis*/*trans*_ = 1.93) and (*S*)-PEA (*K*_*cis*/*trans*_ = 1.97) cations as compared with *trans*-**1**. Both *trans*-**1** an *cis*-**1** have a slight preference for the *R*-PEA enantiomer over the *S*-PEA enantiomer, in contrast to other host–guest systems based on a sucrose scaffold developed in our laboratory.^10b,20^ Only in one case has the 21-membered host, bearing two amide groups, shown the complexing ability with a preference for the *R* enantiomer of α-PEA.^10a^ Notably, *cis*-**1** exhibits a different binding mode for PEA as compared with alkali metal cations; i.e., addition of PEA enantiomers [clearly the effect of (*R*)-PEA is stronger than that of (*S*)-PEA] causes the upfield shift and metal cations the downfield shift of the signal corresponding to the anomeric sugar moiety (H1). This might be attributed to the π–π stacking between the aromatic part of PEA and benzyl groups of the sugar moiety.

In conclusion, we demonstrated the first example of a macrocyclic host system in which cation binding properties could be reversibly controlled by visible light. In contrast to the photoresponsive macrocyclic systems reported to date, the key ring closure step was remarkably efficient and cation-dependent, allowing the preparation of *trans*-**1** in 85.3% yield, while Cs_2_CO_3_ was employed as a dual base and templating agent. *trans*-**1** is converted into long-lived *cis*-**1** (*t*_1/2_ = 25 days) upon irradiation with green light (530 nm), and reverse isomerization is driven by blue light (410 nm). Both isomers have a preference for the potassium cation, and *cis*-**1** exhibits higher binding affinity and selectivity for cations than *trans*-**1**, including chiral phenylethylammonium guests (*K_cis_*/*K_trans_* ≤ 4.1). DFT calculations and ^1^H NMR titrations reveal that, besides the polyether residues, the sucrose ring oxygen and azobenzene nitrogen atoms markedly contribute to cation recognition.

## Supporting Information Available

Materials and methods, synthetic procedures and full characterization of new compounds, spectroscopic data, copies of NMR spectra, and computational and ^1^H NMR titration details (PDF)

## Dedication

The authors dedicate this paper to Professor Janusz Jurczak on the occasion of his 80th birthday.
