# Supplementary material for: Visible-Light Responsive Sucrose-Containing Macrocyclic Host for Cations
Source: Org Lett. 2021 Mar 17;23(7):2687–92. doi: 10.1021/acs.orglett.1c00590 (PMC8041374; doi:10.1021/acs.orglett.1c00590)
Supplement: Supplementary file 1 — ol1c00590_si_001.pdf [file ol1c00590_si_001.pdf]

Supporting Information for

Visible-Light Responsive Sucrose-Containing Macrocyclic Host for Cations

Patrycja Sokołowska, Kajetan Dąbrowa,* and Sławomir Jarosz*

Institute of Organic Chemistry, Polish Academy of Sciences, ul. Kasprzaka 44/52, 01-224 Warsaw, Poland

E-mail address: S.J.: slawomir.jarosz@icho.edu.pl , K.D.: kdabrowa@icho.edu.pl

Contents

1. GENERAL REMARKS 3

1.1. Introduction 3

1.2. Computational details 3

2. SYNTHESIS 4

2.1. Synthesis of 6,6′-di-*O*-tritylsucrose (3) 4

2.2 Synthesis of 1′,2,3,3′,4,4′-hexa-*O*-benzyl-6,6′-di-*O*-tritylsucrose (4) 4

2.3. Synthesis of 1′,2,3,3′,4,4′-hexa-*O*-benzylsucrose (5) 5

2.4. Synthesis of 1′,2,3,3′,4,4′-hexa-*O*-benzyl-6,6′-bis[(2-chloroethoxy)-ethylsucrose (6). 5

2.5. Synthesis of 1′,2,3,3′,4,4′-hexa-*O*-benzyl-6,6′-bis[(2-iodoethoxy)ethylsucrose (7). 6

2.6. General procedure for the synthesis of host 1 7

2.7. Control experiments with TBA salts of carbonate, methoxide, and chloride 7

2.8. Synthesis of (S)-1-phenylethylamine trifluoromethanesulfonate (11). 8

2.9. Synthesis of (R)-1-phenylethylamine trifluoromethanesulfonate (13). 8

2.10. Synthesis of cesium trifluoromethanesulfonate (15). 9

3. PHYSICOCHEMICAL AND PHOTOCHEMICAL PROPERTIES OF *TRANS*-1 AND *CIS*-19

3.1. Characterization of host *trans*-1. 9

3.2. Characterization of host *cis*-1. 11

3.3. Photochemical properties of hosts *trans*-1 and *cis*-1 and their complexes with cations 13

4. TITRATION EXPERIMENTS 15

4.1. General remarks. 15

4.2. Titration spectra 17

5. COPIES OF THE NMR SPECTRA 29

6. CARTESIAN COORDINATES OF CALCULATED STRUCTURES 60

7. REFERENCES 66

1. General remarks

1.1. Introduction

Commercially available reagents were purchased from Sigma-Aldrich, Alfa Aesar or Th.Geyer, and used without purification as received. Hexanes (65-80˚C fraction from petroleum) and EtOAc were purified by distillation. Thin-layer chromatography was carried out on silica gel 60 F254 (Merck). Compounds were purified using automatic flash chromatography on Buchi glass columns packed with silica gel 60 (230-400 mesh, Merck), using Knauer Smartline system with a Buchi fraction collector. The organic solutions were dried over MgSO4 or Na2SO4. The NMR spectra were recorded on Bruker Avance II 400 MHz (at 400 MHz, 376 MHz, and 100 MHz for ^{1}H, ^{19}F, and, ^{13}C NMR spectra, respectively), Varian VNMRS 500 MHz (at 500 MHz and 125 MHz for ^{1}H and ^{13}C NMR spectra, respectively) or Varian VNMRS 600 MHz (at 600 MHz and 150 MHz for ^{1}H and ^{13}C NMR spectra, respectively) spectrometers using solutions in $CDCl_3$, acetone-d_6, CD_3OD or CD_3CN, and TMS or $TBAPF_6$ as the internal standard at 303K. All significant resonances were assigned by COSY (1H-1H), HSQC (1H-^{13}C) and HMBC (^{1}H-^{13}C) correlations. Mass spectra were measured on Synapt G2-S HDMS (Waters Inc) mass spectrometer equipped with an electrospray ion source and q-TOF type mass analyzer. Elemental analyses were obtained with a Perkin–Elmer 2400 CHN analyzer. Absorption spectra were recorded at room temperature unless otherwise stated in MeCN (for UV-spectroscopy, Fluka) on a Jasco J-715 spectropolarimeter with concentrations of 7.0 × 10–5 M in 0.1 cm quartz cell. All spectra were recorded using a 100 nm/min scanning speed, a step size of 0.2 nm, a bandwidth of 1 nm, a response time of 0.5 s, and an accumulation of 5 scans. The baseline of the spectra was corrected by subtracting the spectrum of the pure solvent recorded under the same conditions. Optical rotations were measured with a Jasco P 1020 polarimeter (sodium light) in chloroform at room temperature.

As light source the LED bulbs were used (commercial 3.3W GU10 bulbs containing 25 individual diodes: blue light λ_{max} = 410±5 nm, green light λ_{max} = 530±5 nm) and the sample of host **1** in the UV-cuvettes (quartz glass) was kept at ca. 2.5cm distance from the bulb. No external cooling and light filter were used during experiments.

1.2. Computational details

To account for the solvent effect on the conformations and energies of the studied compounds, a conductor-like polarizable continuum model (C-PCM) in acetonitrile (ε = 37.5) was employed. Firstly, the structures of *trans*-**1** and *cis*-**1** and their complexes with potassium cation were subjected to comprehensive molecular mechanics simulations using the Spartan’18 software package. The obtained structures (up to 200 conformers) were then geometry optimized without any constrains at the semiempirical PM6 level of theory. Energies of these structures were then calculated at DFT/B3LYP-G3/6-31G(d) level of theory. Over a dozen conformers with the lowest energies were then subjected to final calculations using C-PCM solvent approximation.

2. Synthesis

2.1. Synthesis of 6,6′-di-*O*-tritylsucrose (3)

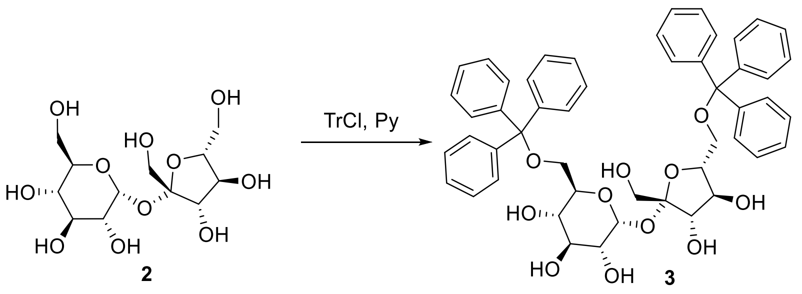

The compound was prepared as previously reported.[1]

Sucrose **2** (10.0 g, 29 mmol) was dissolved in boiling pyridine (170 mL, an oil bath was used for heating) containing DMAP (ca 40 mg). After cooling to room temperature, triphenylmethyl chloride (19.0 g; 68 mmol) was added in one portion and the mixture was stirred at rt for 48 h. After cooling to room temperature, water (150 mL) was added and the products were extracted with ethyl acetate (400 mL). Combined organic solutions were washed with water (2 × 150 mL), brine (150 mL), dried over Na_2SO_4, concentrated, and the resulting residue was purified by column chromatography (hexane/ethyl acetate = 1:2 to 100% ethyl acetate) to afford 6,6′-di-O-tritylsucrose **3** as a white crystalline solid state (12.4 g, 15 mmol, 51%). $[\alpha]_D^{25}$ = +39.5 (*c* 0.5, $CHCl_3$). HRMS (ESI) m/z: $[M + Na]^+$ Calcd for $C_{50}H_{50}O_{11}Na$ 849.3245; Found 849.3287. Anal. Calcd for $C_{50}H_{50}O_{11}$: C, 72.62; H, 6.09. Found: C, 72.36; H, 6.30.

2.2 Synthesis of 1′,2,3,3′,4,4′-hexa-*O*-benzyl-6,6′-di-*O*-tritylsucrose (4)

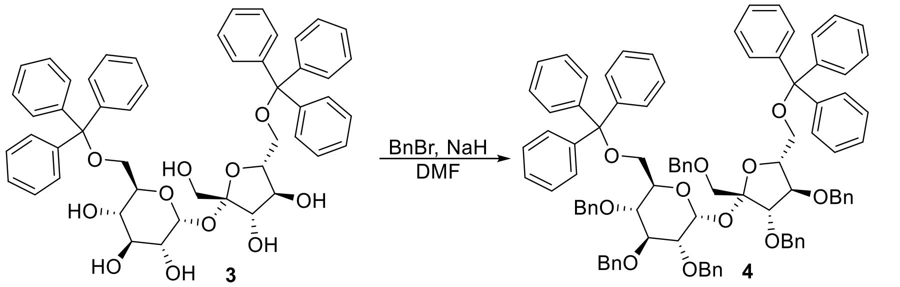

The compound was prepared as previously reported.[1]

To a stirred solution of compound **3** (3.8 g, 4.6 mmol) in DMF (100mL), was added sodium hydride (60% dispersion in mineral oil, 1.45 g, 36 mmol) and the mixture was stirred at room temperature for 30 min. Benzyl bromide (3.7 ml, 31.2 mmol) was added dropwise during 30 min and the mixture was stirred at rt for another 12h. Excess of hydride was decomposed carefully with methanol (5ml) and the mixture was partioned between water (100ml) and ethyl acetate (100ml). The organic phase was dried over Na_2SO_4, concentrated and the resulting residue was purified by column chromatography (hexane/ethyl acetate = 15:1 to 6:1) to afford the pure product 4 as a white amorphous solid (5.03 g, 3.7 mmol, 80%). $[\alpha]_D^{25}$ = +19.8 (*c* 0.7, $CHCl_3$). HRMS (ESI) m/z: $[M + Na]^+$ Calcd for $C_{92}H_{86}O_{11}Na$ 1389.6067; Found 1389.6012. Anal. Calcd for $C_{92}H_{86}O_{11}$: C, 80.79; H, 6.34. Found: C, 80.62; H, 6.43.

2.3. Synthesis of 1′,2,3,3′,4,4′-hexa-*O*-benzylsucrose (5)

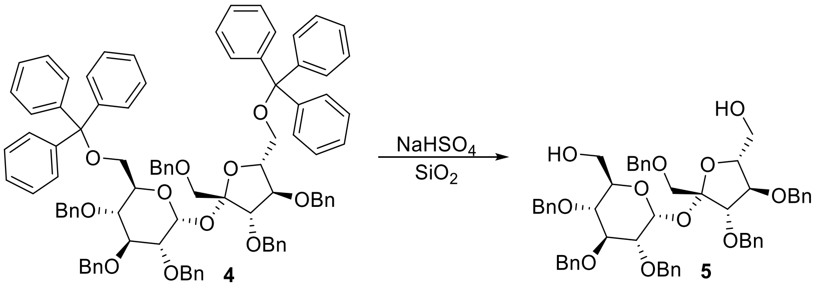

The above prepared 4 (0.5 g, 0.37 mmol) was dissolved in a CH_2Cl_2/MeOH mixture (10:1 v/v; 5 mL) and the solution was stirred at rt for 5 min. NaHSO4 (0.3 g) and SiO_2 (1.2 g) was added and this mixture was stirred at rt until TLC (hexane/ethyl acetate = 9:1) indicated disappearance of the starting material (ca. 20 h). The mixture was filtered and the residue was purified by flash chromatography (hexane/ethyl acetate = 9:1) to afford 6,6′-di-O-tritylsucrose 5 as a colorless oil (194 mg, 0.22 mmol, 60%). $[\alpha]_D^{25}$ = +41.0 (*c* 0.5, $CHCl_3$).

^{1}H NMR ($CDCl_3$, 600 MHz): δ 5.49 (d, 1H, *J*1,2 = 3.6, H-1), 4.86 (dd, 2H, 2 × OC*H*2Ph), 4.76 (d, 1H, *J* = 10.9, OC*H*2Ph), 4.71 (d, 1H, *J* = 11.5, OC*H*2Ph), 4.69 (d, 1H, *J* = 11.6, OC*H*2Ph), 4.67 (d, 1H, *J* = 12.0, OC*H*2Ph), 4.62 (d, 1H, *J* = 11.6, OC*H*2Ph), 4.60 (d, 1H, *J* = 11.1, OC*H*2Ph), 4.57 (d, 1H, *J* = 11.5, OC*H*2Ph), 4.49 (d, 1H, *J* = 11.7, OC*H*2Ph), 4.47 (d, 1H, *J* = 12.1, OC*H*2Ph), 4.42 (d, 1H, *J* = 7.6, H-3’), 4.33 (t, 1H, *J* = 7.9, H-4’), 4.31 (d, 1H, *J* = 12.0, OC*H*2Ph), 4.15 (ddd, 1H, *J* = 10.1, 5.1, 1.9, H-5), 3.99 (t, 1H, *J* = 9.4, H-3), 3.96 (dt, 1H, *J* = 8.1, 2.4, H-5’), 3.82 (m, 2H, H-6, H6’), 3.63 (dd, 1H, *J* = 12.2, 5.2, H-6), 3.59 (dd, 1H, *J* = 12.7, 2.7, H-6’), 3.57 (d, 1H, *J* = 11.0, OC*H*2Ph), 3.50 (dd, 1H, *J* = 9.7, H-2), 3.45 (d, 1H, *J* = 11.0, OC*H*2Ph), 3.43 (m, 1H, H-4) ppm.

^{13}C{1H} NMR ($CDCl_3$, 151 MHz): δ 138.6, 138.3, 138.1, 138.1, 138.0, 137.7 (C_{quat}, 6 × OCH_2*Ph*), 103.9 (C-2'), 90.6 (C-1), 83.5 (C-3'), 81.7 (C-4’), 80.9 (C-3), 79.8 (C-2), 79.5 (C-5’), 77.6 (C-4), 75.5, 74.9, 73.4, 73.3, 73.0 (5×O*C*H_2Ph), 72.9, 72.4, 71.3, 61.9, 60.9 (C-5, C-6’, C-6, C-1’, O*C*H_2Ph) ppm.

HRMS (ESI) m/z [M + Na]$^+$ Calcd for $C_{54}H_{58}O_{11}Na$ 905.3876; Found 905.3906; Anal. Calcd for $C_{54}H_{58}O_{11}$: C, 73.45; H, 6.62. Found: C, 73.04; H, 6.53.

2.4. Synthesis of 1’,2,3,3’,4,4’-hexa-*O*-benzyl-6,6’-bis[(2-chloroethoxy)-ethylsucrose (6).

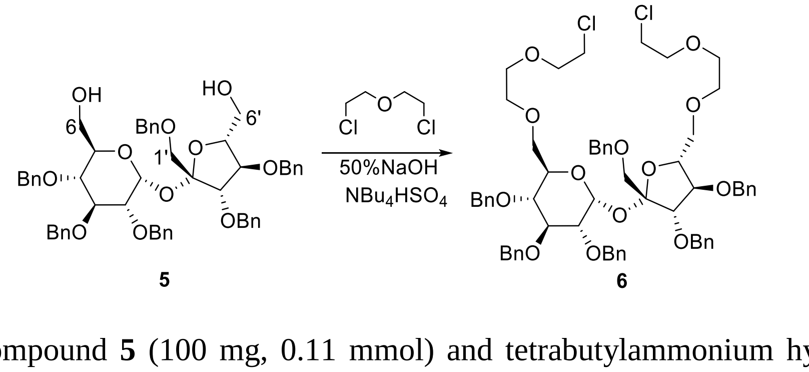

A solution of compound **5** (100 mg, 0.11 mmol) and tetrabutylammonium hydrogensulfate (38.5 g, 0.11 mmol) in bis(2-chloroethyl) ether (284 µL, 2.42 mmol) was vigorously stirred with 50% NaOH solution (423 µL) at room temperature for 3 h. Then CH_2Cl_2 (1.5 mL) and

water (1.5 mL) were added, the organic layer was separated, and the aqueous one extracted with CH_2Cl_2 (2 x 5 mL). The combined organic solutions were washed with water (2 x 5 mL), dried, and concentrated under high vacuum to remove the excess of bis(2-chloroethyl)ether. The crude material was purified by column chromatography (hexane/ethyl acetate = 80:20) to afford the title product **6** (92 mg, 0.08 mmol, 74%) as an oil. $[\alpha]_D^{25}$ = +29.9 (*c* 0.6, $CHCl_3$).

^{1}H NMR ($CDCl_3$, 600MHz) δ 5.66 (d, 1H, $J_{1,2}$ = 3.6, H-1), 4.90 (d, 1H, *J* = 10.9, OC<u>*H*</u>2Ph), 4.86 (d, 1H, *J* = 11.0, OC<u>*H*</u>2Ph), 4.76 (d, 1H, *J* = 10.9, OC<u>*H*</u>2Ph), 4.67 (d, 1H, *J* = 11.4, OC<u>*H*</u>2Ph), 4,64 (dd, 2H, OC<u>*H*</u>2Ph), 4.58-4.54 (m, 4H, OC<u>*H*</u>2Ph), 4.52(d, 1H, *J* = 11.4, OC<u>*H*</u>2Ph), 4.44-4.40 (m, 2H, OC<u>*H*</u>2Ph, H-3'), 4.10 (m, 2H, H-4', H-5'), 4.04 (ddd, 1H, *J* = 10.2, 3.3, 1.9, H-5), 3.94 (t, *J* = 9.3, H-3), 3.75 (d, 1H, *J* = 11.0, H-1'a), 3.72-3.45 (m, 21H, -OC<u>*H*2</u>-, H-4, H-2, 2×C<u>*H*2</u>-Cl), 3.42 (dd, 1H, *J* = 10.8, 1.8 Hz, H-6a) ppm.

^{13}C{1H} NMR ($CDCl_3$, 151 MHz) δ 138.9, 138.8, 138.4, 138.3, 138.3, 137.9 (C_{quat}, 6 × OCH2<u>*Ph*</u>), 104.7 (C-2'), 90,2 (C-1), 83.9 (C-3'), 82.6 (C-4'), 81.9 (C-3), 79.8 (C-2), 79.8 (C-5'), 77.5 (C-4), 75.5, 74.8, 73.4, 72.9, 72.3 (5 × O<u>C</u>H2Ph), 72.7 (C6'), 72.4, 71.4, 71.3, 71.0, 70.8, 70.5, 70.5 (C-7, C-8, C-9, C-7', C-8', C9', C1'), 70.6 (C-5), 69.7 (C-6), 42.8, 42.7 (2 × <u>*C*</u>H2Cl) ppm.

HRMS (ESI) m/z $[M + Na]^+$ Calcd for $C_{62}H_{72}O_{13}Cl_2Na$ 1117.4248; Found 1117.4211; Anal. Calcd for $C_{62}H_{72}O_{13}Cl_2$: C, 67.94; H, 6.62; Cl, 6.47. Found: C, 67.94; H, 6.85; Cl, 6.43.

2.5. Synthesis of 1',2,3,3',4,4'-hexa-*O*-benzyl-6,6'-bis[(2-iodoethoxy)ethylsucrose (7).

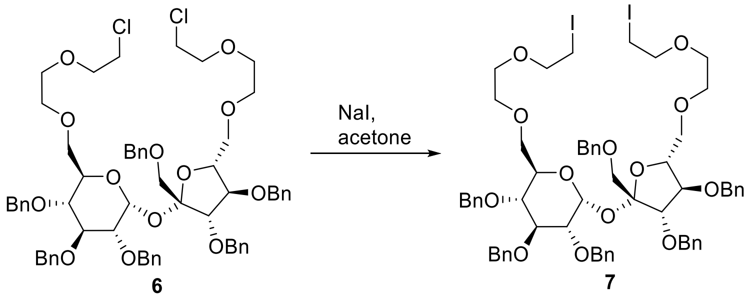

A solution of the *bis*-chloro derivative **6** (813mg, 0.74 mmol) in dry acetone (16 mL) containing dry sodium iodide (444.7mg, 2.97 mmol) was stirred and boiled under reflux for 24 h (an oil bath). After cooling to rt., the precipitate was filtered off and washed with acetone. The combined acetone solutions were concentrated, and the residue was dissolved in CH_2Cl_2 (10 mL). The organic phase was washed with water and dried to give **7** (900 mg, 0.73 mmol, 95 %) as an oil. $[\alpha]_D^{25}$ = +25.5 (*c* 0.6, $CHCl_3$).

^{1}H NMR ($CDCl_3$, 600MHz) δ 5.65 (d, 1H, $J_{1,2}$ = 3.6, H-1), 4.90 (d, 1H, *J* = 10.9, OC<u>*H*</u>2Ph), 4.86 (d, 1H, *J* = 11.0, OC<u>*H*</u>2Ph), 4.76 (d, 1H, *J* = 10.9, OC<u>*H*</u>2Ph), 4.68 (d, 1H, *J* = 11.4, OC<u>*H*</u>2Ph), 4,64 (dd, 2H, OC<u>*H*</u>2Ph), 4.58-4.54 (m, 4H, OC<u>*H*</u>2Ph), 4.43-4.41 (m, 2H, OC<u>*H*</u>2Ph, H-3), 3.75 (d, 1H, *J* = 11.0, H-1'a), 3.71-3.47 (m, 17H, -OC<u>*H*2</u>-, H-4, H-2), 3.42 (dd, 1H, *J* = 10.8, 1.8 Hz, H-6a), 3.19 – 3.16 (m, 2H, C<u>*H*2</u>I), 3.15 (t, *J* = 6.9 Hz, 2H, C<u>*H*2</u>I) ppm.

^{13}C{1H} NMR ($CDCl_3$, 151 MHz) δ: 138.9, 138.8, 138.4, 138.3, 138.3, 137.9 (C_{quat}, 6 × OCH2<u>*Ph*</u>), 104.8 (C-2'), 90,3 (C-1), 83.9 (C-3'), 82.7 (C-4'), 81.9 (C-3), 79.8 (C-2), 79.8 (C-

5'), 77.5 (C-4), 75.5, 74.9, 73.4, 72.9, 72.3 (5 × O<u>C</u>H_2Ph), 72.7 (C6'), 72.4, 72.0, 71.9, 70.8, 70.8, 70.1, 70.1 (C-7, C-8, C-9, C-7', C-8', C9, C1), 70.6 (C-5), 69.7 (C-6), 3.1, 2.9 (2 × <u>C</u>H_2I) ppm.

HRMS (ESI) m/z $[M + Na]^+$ Calcd for $C_{62}H_{72}O_{13}I_2Na$ 1301.2960; Found 1301.2955; Anal. Calcd for $C_{62}H_{72}O_{13}I_2$: C, 58.22; H, 5.67; I, 19.84. Found: C, 58.12; H, 5.67; I, 19.79.

2.6. General procedure for the synthesis of host 1

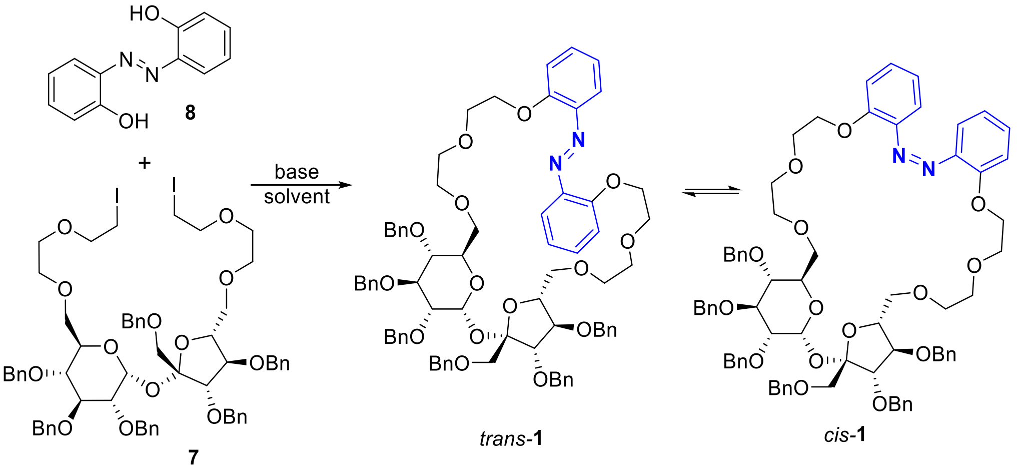

To a solution of **7** (120 mg, 0.09 mmol) and **8** (20 mg, 0.09 mmol in dry acetonitrile (5mL), powdered cesium carbonate (244 mg, 0.75 mmol) was added and the mixture was heated to 82°C using an oil bath and stirred for 6 hours under argon atmosphere. After cooling to rt., water (10 mL) was added and the products were extracted with ethyl acetate (2 × 10 mL). The organic phase was dried over Na_2SO_4, concentrated and the resulting residue was purified by column chromatography (hexane/ethyl acetate = 4:1 to 1:1 v/v) to afford pure compounds *trans*-**1** and *cis*-**1**$^\$$ as color glaze (99 mg, 0.08 mmol, 85.3 %).

$ T*rans*-**1** spontaneously isomerize to *cis*-**1** during workup and column chromatography under ambient light. The procedure for preparation of pure *trans*-**1** is given in Section 3.1.

2.7. Control experiments with TBA salts of carbonate, methoxide, and chloride

<u>Macrocyclisation using di-tetrabutylammonium carbonate (TBA_2CO_3):</u>

The tetrabutylammonium carbonate (TBA_2CO_3) was prepared by the reported procedure.[2]
To a solution of **7** (52 mg, 0.041 mmol) and **8** (8.7 mg, 0.041 mmol) in MeCN (3mL) was added TBA_2CO_3 (177 mg, 0.32 mmol) and the mixture was heated to 82°C using an oil bath and stirred for 24 hours under argon atmosphere. After cooling to rt. water (10 mL) was added and the solution was extracted with ethyl acetate (2 × 10 mL), dried over anhydrous Na_2SO_4 and con-centrated. Analysis of the residue reveals only traces of product **1** along with a substantial amount of unidentified polar residues, probably oligomers derived from **7** and **8**.

<u>Macrocyclisation using tetrabutylammonium methoxide (TBAOMe):</u>

To a solution of **7** (54 mg, 0.042 mmol) and **8** (9 mg, 0.042 mmol) in MeCN (3mL) was added TBAOMe$^\$$ (92.4 mg, 0.34 mmol) and the reaction mixture was stirred at 82°C for 24 hours

under argon atmosphere. After cooling to rt. water (10 mL) was added and the solution was extracted with ethyl acetate (2 × 10 mL), dried over anhydrous Na_2SO_4 and concentrated. Analysis of the residue reveals only traces of product **1** along with some amount of terminal olefin derived by methoxide E2-type elimination of α,ω-diiodo substrate **7**.

$ Neat TBAOMe was prepared by low-temperature evaporation of solvent under reduced pressure from the commercially available 20% wt TBAOMe in MeOH and was used immediately in the macrocyclisation reaction.

Additional ^{1}H NMR experiment reveals that host 1 is stable to TBAOMe, at least for 48 in refluxing MeCN-d_3.

Macrocyclisation using tetrabutylammonium chloride (TBACl) and cesium carbonate (Cs_2CO_3):

To a solution of **7** (50 mg, 0.039 mmol) and **8** (8.4 mg, 0.039 mmol) in acetonitrile (3mL) were added sequentially Cs_2CO_3 (85.5 mg, 0.31 mmol) and TBACl (201 mg, 0.72 mmol) and the mixture was heated to 82°C using an oil bath and stirred for 6 hour under argon atmosphere. After cooling to rt. water (10 mL) was added and the solution was extracted with ethyl acetate (2 × 10 mL), dried over anhydrous Na_2SO_4, concentrated, and the resulting residue was purified by column chromatography (hexane/ethyl acetate = 4:1 to 1:1 v/v) to afford trans/cis-mixture of **1** as color glaze (18 mg, 0.015 mmol, 37 %).

2.8. Synthesis of (S)-1-phenylethylamine trifluoromethanesulfonate (11).

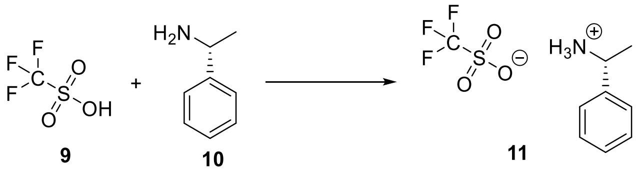

A solution of trifluoromethanesulfonic acid (192 µl, 2.2 mmol) in diethyl ether (20 ml) was vigorously stirred under argon atmosphere at room temperature. Then (S)-(-)-1-Phenylethylamine (280 µl, 2.2 mmol) was added dropwise in 10 min. The white solid precipitate was filtered, and the cake was washed with a minimal amount of cold diethyl ether and then dried in a vacuum oven overnight to afford 561 mg (93%) of white solid product. ^{1}H NMR (CD_3CN, 400MHz) δ 7.57 – 7.29 (m, 5H), 6.84 (s, 2H), 4.53 (q, J = 6.9 Hz, 1H), 1.63 (d, J = 6.9 Hz, 3H). ^{13}C{1H} NMR (CD_3CN, 101 MHz) δ: 138.39, 130.27, 130.09, 127.94, 53.26, 20.36, 20.26 ppm. ^{19}F NMR (CD_3CN, 376 MHz) δ: -79.35 ppm. Anal. calcd for $C_9F_3H_{12}NO_3S$: C, 39.85; H, 4.46; N, 5.16; found: C, 39.85; H, 4.40; N, 5.26.

2.9. Synthesis of (R)-1-phenylethylamine trifluoromethanesulfonate (13).

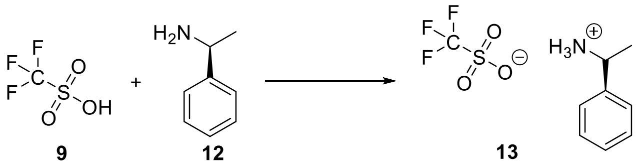

A solution of trifluoromethanesulfonic acid (192 µl, 2.2 mmol) in diethyl ether (20 ml) was vigorously stirred under argon atmosphere at room temperature. Then (S)-(-)-1-Phenylethylamine (280 µl, 2.2 mmol) was added dropwise in 10 min. The white solid precipitate was filtered, and the cake was washed with a minimal amount of cold diethyl ether and then dried in a vacuum oven overnight to afford 556 mg (90%) of white solid product. ^{1}H NMR (CD_3CN, 400MHz) δ 7.57 – 7.29 (m, 5H), 6.84 (s, 2H), 4.53 (q, J = 6.9 Hz, 1H), 1.63 (d, J = 6.9 Hz, 3H). ^{13}C{1H} NMR (CD_3CN, 101 MHz) δ: 138.39, 130.27, 130.09, 127.94, 53.26, 20.36, 20.26 ppm. ^{19}F NMR (CD_3CN, 376 MHz) δ: -79.36 ppm. Anal. calcd for $C_9F_3H_{12}NO_3S$: C, 39.85; H, 4.46; N, 5.16; found: C, 39.86; H, 4.39; N, 5.27.

2.10. Synthesis of cesium trifluoromethanesulfonate (15).

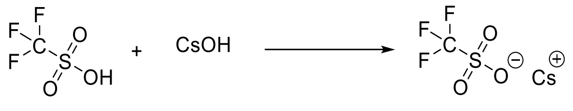

A solution of 10 g (66.6 mmol) of cesium hydroxide in 20 mL of water was added dropwise carefully *via* a dropping funnel to 6.3 ml, 10g (66.6 mmol) of triflic acid chilled in an ice bath. The solution was stirred at 0°C for 20 min, then it was concentrated to dryness using a rotary evaporator, and MeCN (~ 50mL) was added to the residual solid and the procedure was repeated. The white solid was dissolved while hot in the minimum amount of MeCN, and then it was precipitated by the addition of Et_2O (100 mL). The solid was filtered on a glass funnel G4, washed with Et_2O (~50 mL), and dried in a vacuum oven overnight to afford 14.9 g of pure cesium triflate (79%). ^{19}F NMR (CD_3CN, 376 MHz) δ: -79.65 ppm. Anal. calcd for $CsCF_3SO_3$: C, 4.26; S, 11.37; found: C, 4.25; S, 11.37.

3. Physicochemical and photochemical properties of *trans*-1 and *cis*-1

3.1. Characterization of host *trans*-1.

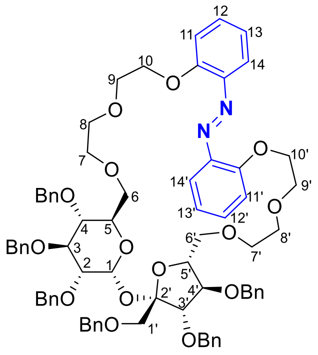

***trans*-1**

Host *trans*-**1** is the thermodynamically stable isomer, albeit its broad absorption in the visible region of the light (400-600 nm) induces partial *trans*-to-*cis* isomerization even by ambient light. To obtain pure *trans*-**1**, the compound was kept in an amber glass container at high temperature (up to ~70°C for a solution of **1** in MeCN) for an appropriate time (usually 24-48h).

Afterwards, the pure *trans*-**1** was covered with aluminum foil to prevent incidental light exposure. Solutions of *trans*-**1** in non-amber glass for the ^{1}H NMR experiments were handled under red-light conditions to prevent any isomerization.

Trans-enriched mixture of **1** was prepared upon irradiation with blue light (410 nm, LED 3W) for varying amounts of time, depending on the concentration of the solution used (~2-4 min for 1-6·10^{-5}M and up to 20 min for ~10^{-2}M). After irradiation, the *trans*-enriched solution of host **1** was covered with aluminum foil to ensure no changes in the *trans*/*cis* ratio due to incident light.

^{1}H NMR (CD_3CN, 600MHz) δ: 7.38 (ddd, 1H, *J* = 8.4, 7.3, 1.8, H-14), 7.37 (ddd, 1H, *J* = 8.3, 7.3, 1.7, H-14'), 7.32 (m, 2H, H-11, H-11'), 7.12 (dd, 1H, *J* = 8.3, 1.2, H-13), 7.09 (dd, 1H, *J* = 8.4, 1.1, H-13'), 7.0 (dddd, 2H, *J* = 7.9, 7.3, 1.7, 1.1, H-12, H-12'), 5.49 (d, 1H. *J* = 3.5, H-1), 4.77 (d, 1H, *J* = 11.2, (C-3)OC$\underline{H}_2$Ph), 4.71 (d, 1H, *J* = 11.1, (C-4)OC$\underline{H}_2$Ph), 4.66 (d, 1H, *J* = 11.2, (C-3)OC$\underline{H}_2$Ph), 4.69-4.58 (m, 3H, (C-3')OC$\underline{H}_2$Ph, (C-4')OC$\underline{H}_2$Ph, (C-2)OC$\underline{H}_2$Ph, 4.54 (d, 1H, *J* = 11.9, (C-4')OC$\underline{H}_2$Ph), 4.51-4.46 (m, 4H, (C-4)OC$\underline{H}_2$Ph, (C-2)OC$\underline{H}_2$Ph, (C-3')OC$\underline{H}_2$Ph, (C-1')OC$\underline{H}_2$Ph), 4.39 (d, 1H, *J* = 11.9, (C-1')OC$\underline{H}_2$Ph), 4.28 (d, 1H, *J* = 7.8, H-3'), 4.26-4.22 (m, 4H, 2×H-10, 2×H-10'), 3.98 (t, 1H, *J* = 7.9, H-4'), 3.82-3.72 (m, 6H, H-5, H-5', 2×H-9, 2×H-9'), 3.71 (t, 1H, *J* = 9.3, H-3), 3.57-3.50 (m, 6H, H-6', H-1', 2×H-8, 2×H-8'), 3.48 (dd, 1H, *J* =11.0, 3.3, H-6'), 3.43-3.35 (m, 5H, H-1', 2×H-7', H-4, H-7), 3.34 (dd, 1H, *J* = 9.7, 3.3, H-2), 3.29 (dd, 1H, *J* = 11.2, 3.7, H-6), 3.25-3.21 (m, 1H, H-7), 3.19 (dd, 1H, *J* = 11.2, 1.7, H-6) ppm.

^{13}C{1H} NMR (CD_3CN, 151 MHz) δ: 155.40, 155.33 (C_{quat}, 2× N=N$\underline{Ph}$), 143.88, 143.85 (C_{quat}, 2× O$\underline{Ph}$), 139.24, 139.04, 138.66, 138.54, 138.49, 138.30 (C_{quat}, 6× $OCH_2\underline{Ph}$), 131.93, 131.92 (C-14, C-14'), 128.34, 128.29, 128.28, 128.23, 128.21, 128.18, 127.98, 127.91, 127.86, 127.81, 127.71, 127.67, 127.63, 127.56, 127.53, 127.50, 127.40, 127.34 (18×$OCH_2\underline{Ph}$), 120.97, 120.95 (C-12, C-12'), 117.96, 117.92 (C-11, C-11'), 114.79 (C-13'), 114.72 (C-13), 103.92 (C-2'), 89.15 (C-1), 83.25 (C-3'), 81.60 (C-3), 81.44 (C-4'), 79.90 (C-2), 79.37 (C-5'), 77.49 (C-4), 74.78 ((C-3)O$\underline{C}H_2$Ph), 74.24 ((C-4)O$\underline{C}H_2$Ph), 72.93 ((C-1')O$\underline{C}H_2$Ph), 72.32 ((C-3')O$\underline{C}H_2$Ph), 72.10 ((C-4')O$\underline{C}H_2$Ph), 72.03 ((C-2)O$\underline{C}H_2$Ph), 71.77 (C-6'), 71.74 (C-1'), 70.68 (C-5), 70.62 (C-8), 70.60 (C-7), 70.38 (C-7'), 70.28 (C-8'), 69.56 (C10), 69.53 (C6), 69.41 (C-10'), 69.40 (C-9), 69.33 (C-9') ppm. HRMS (ESI) m/z $[M + Na]^+$ Calcd for $C_{74}H_{80}N_2O_{15}Na$ 1259.5456; Found 1259.5486; Anal. Calcd for $C_{74}H_{80}N_2O_{15}$: C, 71.83; H, 6.52; N, 2.26. Found: C, 71.66; H, 6.52; N, 2.26.

3.2. Characterization of host *cis*-1.

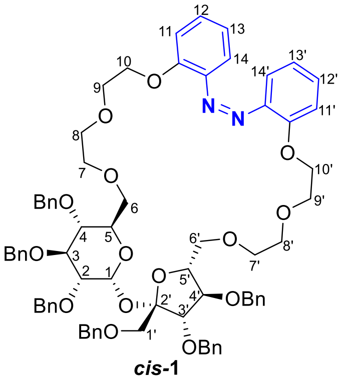

***cis*-1**

Host *cis*-**1** was prepared as a *cis*-enriched mixture upon irradiation of the host solution in MeCN with green light (530 nm, LED 3W) for varying amounts of time, depending on the concentration of the solution used (~1-5 min for $1\text{-}6{\cdot}10^{-5}$M and up to 30 min for ~10^{-2}M). After irradiation, the cis-enriched solution of host **1** was covered with aluminum foil to ensure no changes in the trans/cis ratio due to incident light. Due to the relatively high thermal stability of *cis*-isomer (see Table S1), it was assumed that the concentration of *cis*-**1** was kept constant during the experiments in MeCN at rt (the longest experiments took up to ~3h for the NMR measurements).

^{1}H NMR (CD_3CN, 600MHz) δ: 7.09 (m, 2H, H-14, H-14’), 6.87 (ddd, 2H, *J* = 8.4, 6.2, 1.1, H-13, H-13’), 6.76 (td, 1H, *J* = 7.6, 1.1, H-12), 6.73 (td, 1H, *J* = 7.6, 1.1, H-12’), 6.69 (dd, 1H, *J* = 7.8, 1.7, H-11), 6.65 (dd, 1H, *J* = 7.8, 1.7, H-11’), 5.62 (d, 1H. *J* = 3.5, H-1), 4.80 (d, 1H, *J* = 11.2, (C-3)OC<u>H</u>$_2$Ph), 4.76 (d, 1H, *J* = 11.1, (C-4)OC<u>H</u>$_2$Ph), 4.68 (d, 1H, *J* = 11.2, (C-3)OC<u>H</u>$_2$Ph), 4.68 (d, 1H, *J* = 11.9, (C-4’)OC<u>H</u>$_2$Ph, 4.65-4.62 (m, 2H, (C-3’)OC<u>H</u>$_2$Ph, (C-2)OC<u>H</u>$_2$Ph), 4.61 (d, 1H, *J* =11.8, (C-4’)OC<u>H</u>$_2$Ph), 4.55 (d, 1H, *J* =11.3, (C-4)OC<u>H</u>$_2$Ph), 4.54-4.49 (m, 3H, (C-3’)OC<u>H</u>$_2$Ph, (C-2)OC<u>H</u>$_2$Ph, (C-1’)OC<u>H</u>$_2$Ph), 4.41 (d, 1H, *J* = 11.9, (C-1’)OC<u>H</u>$_2$Ph), 4.33 (d, 1H, *J* = 7.8, H-3’), 4.12 (t, 1H, *J* = 7.9, H-4’), 4.0-3.85 (m, 6H, 2×H-10, 2×H-10’, H-5, H-5’), 3.77 (t, 1H, *J* = 9.3, H-3), 3.72 (dd, 1H, H-6’), 3.68-3.62 (m, 5H, 2×H-9, 2×H-9’, H-6’), 3.62-3.50 (m, 8H, 2×H-8, 2×H-8’, 2×H-7’, H-7, H-1’), 3.50-3.45 (m, 2H, H-4. H-1’), 3.45-3.36 (m, 4H, 2×H-6, H-7, H-2) ppm.

^{13}C{1H} NMR (CD_3CN, 151 MHz) δ: 148.70, 148.27 (C_{quat}, 2× N=N<u>*Ph*</u>), 143.78, 143.78 (C_{quat}, 2× O<u>*Ph*</u>), 139.23, 139.00, 138.66, 138.62, 138.52, 138.33 (C_{quat}, 6× OCH$_2$<u>*Ph*</u>), 128.61, 128.60 (C-14, C-14’), 128.61, 128.60, 128.34, 128.32, 128.28, 128.26, 128.23, 128.19, 128.01, 127.93, 127.90, 127.82, 127.74, 127.71, 127.64, 127.59, 127.56, 127.50, 127.44, 127.35 (18×OCH$_2$<u>*Ph*</u>), 120.10 (C-12), 120.00 (C-12’), 119.81 (C-11), 119.58 (C-11’), 113.32, 113.25 (C-13, C-13’), 104.02 (C-2’), 89.20 (C-1), 83.30 (C-3’), 81.64 (C-3), 81.39 (C-4’), 79.95 (C-2), 79.51 (C-5’), 77.66 (C-4), 74.80 ((C-3)O<u>C</u>H$_2$Ph), 74.29 ((C-4)O<u>C</u>H$_2$Ph), 72.95 ((C-1’)O<u>C</u>H$_2$Ph, 72.37, 72.14, 72.09, ((C-3’)O<u>C</u>H$_2$Ph, (C-2)O<u>C</u>H$_2$Ph, (C-4’)O<u>C</u>H$_2$Ph), 71.81 (C-1’), 71.60 (C-6’), 70.81 (C-5), 70.65 (C-7), 70.44, 70.44, 70.25, (C-8, C-8’, C-7’), 69.67 (C-6), 69.14, 69.12 (C-9, C-9’), 68.11, 68.11 (C-10, C-10’) ppm.

HRMS (ESI) m/z $[M + Na]^+$ Calcd for $C_{74}H_{80}N_2O_{15}Na$ 1259.5456; Found 1259.5486; Anal. Calcd for $C_{74}H_{80}N_2O_{15}$: C, 71.83; H, 6.52; N, 2.26. Found: C, 71.66; H, 6.52; N, 2.26.

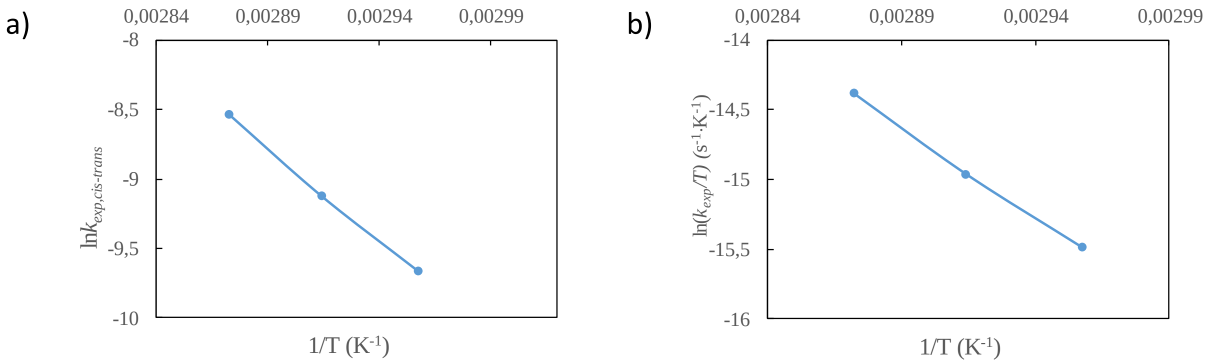

Fig. S1. Arrhenius (a) and van't Hoff (b) plots of thermal *cis*→*trans* isomerization of **1**.

Table S1. Kinetic and thermodynamic data for thermal isomerization of *cis*-**1**[a]

$k_{cis\rightarrow trans}\cdot 10^{-7}(s^{-1})$	$\tau_{1/2}$ (h)	E_a	$\Delta H^{\#}$	$T\Delta S^{\#}$	ΔG[b]
3.23	596	110.5	107.7	-2.4	110.0

[a] Corresponding values at 298K were measured in MeCN and determined using Arrhenius and Eyring equations; $c_{host} = 5\cdot 10^{-5}$ M; the values for E_a, $\Delta H^{\#}$, and $\Delta G^{\#}$ are given in $kJ\cdot mol^{-1}$, and for $T\Delta S\#$ in $J\cdot mol^{-1}$. [b] Calculated using equation: $\Delta H - T\Delta S$.

3.3. Photochemical properties of hosts *trans*-1 and *cis*-1 and their complexes with cations

Host *cis*-**1** was prepared

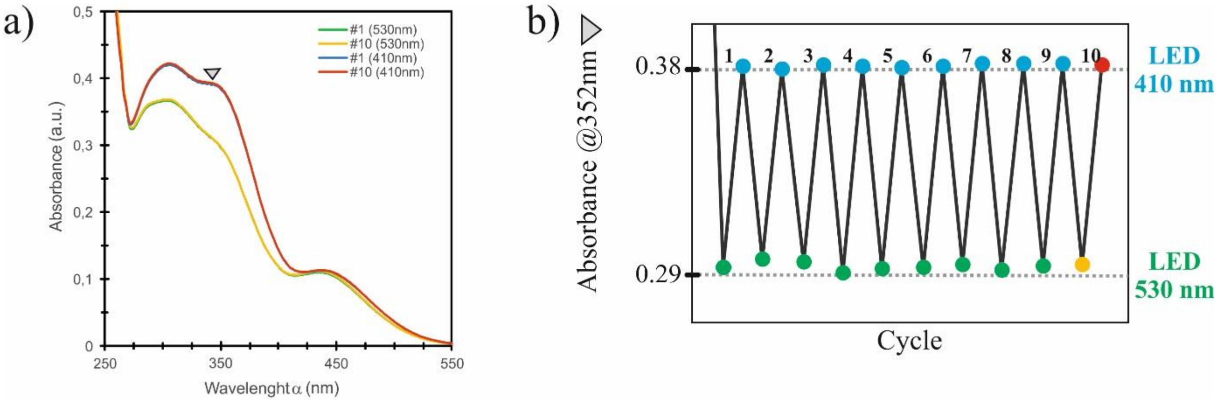

Fig. S2. Photoswitching between *trans*-1 and *cis*-1 in MeCN at 298.0±0.1K using green light (530 nm) and blue light (410 nm), respectively.

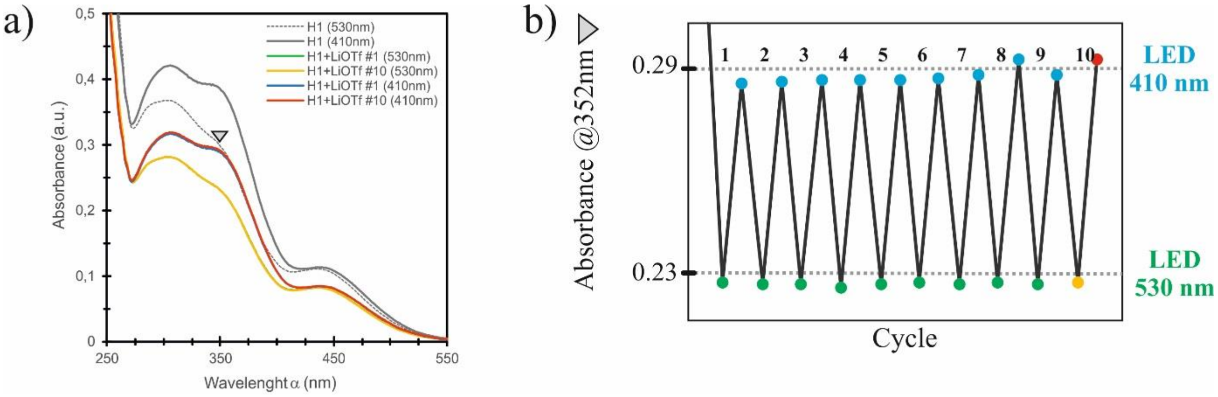

Fig. S3. Photoswitching between complexes of *trans*-1 and *cis*-1 with LiOTf (100 equiv) in MeCN at 298.0±0.1K using green light (530 nm) and blue light (410 nm), respectively.

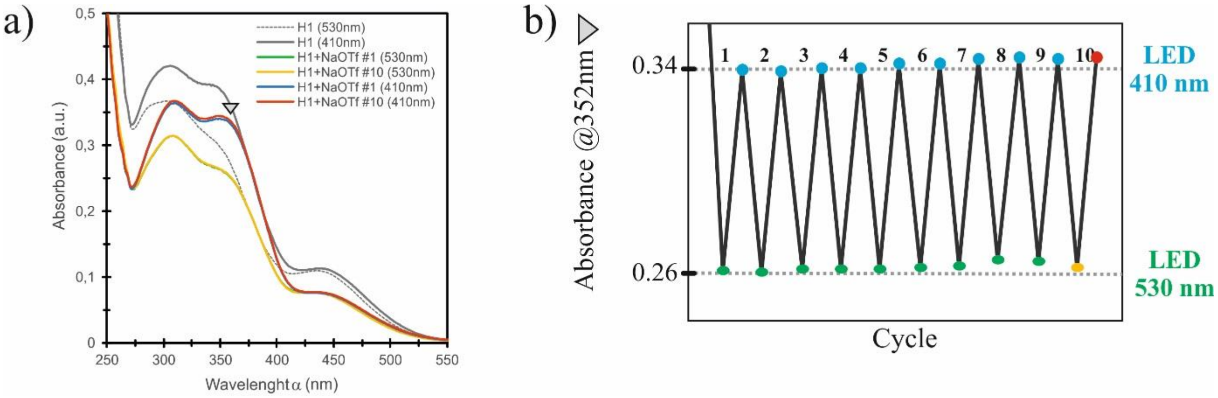

Fig. S4. Photoswitching between complexes of *trans*-1 and *cis*-1 with NaOTf (100 equiv) in MeCN at 298.0±0.1K using green light (530 nm) and blue light (410 nm), respectively.

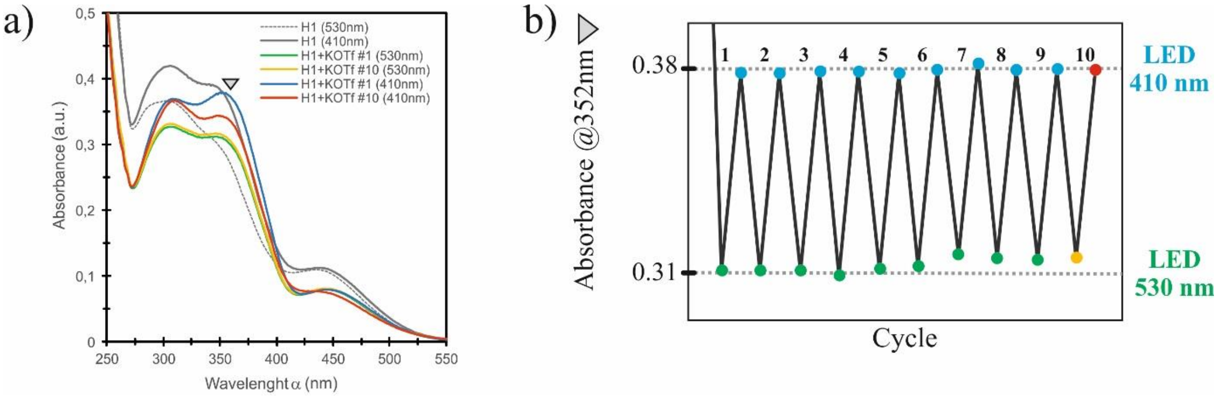

Fig. S5. Photoswitching between complexes of *trans*-1 and *cis*-1 with KOTf (10 equiv) in MeCN at 298.0±0.1K using green light (530 nm) and blue light (410 nm), respectively.

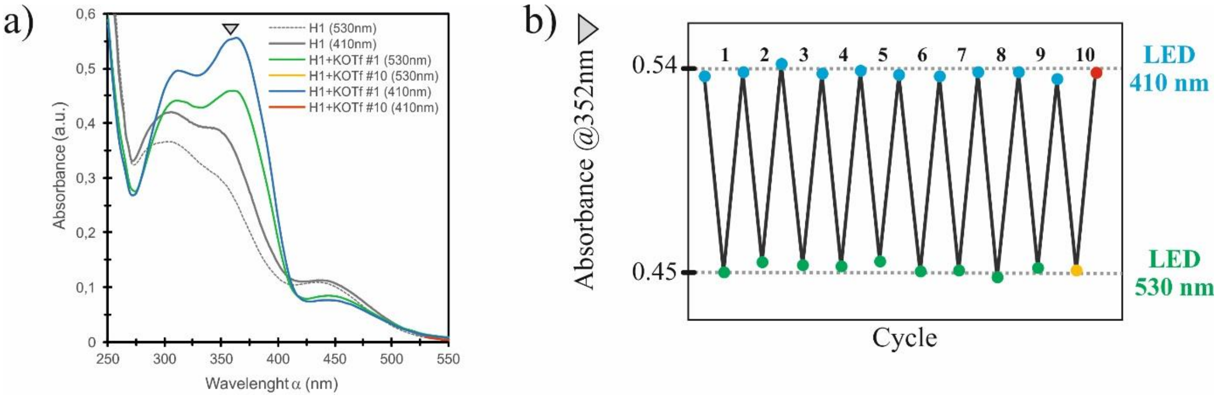

Fig. S6. Absorption spectra and photoswitching of a solution of *trans/cis*-1 with KOTf (100 equiv) in MeCN at 298.0±0.1K using green light (530 nm) and blue light (410 nm), respectively.

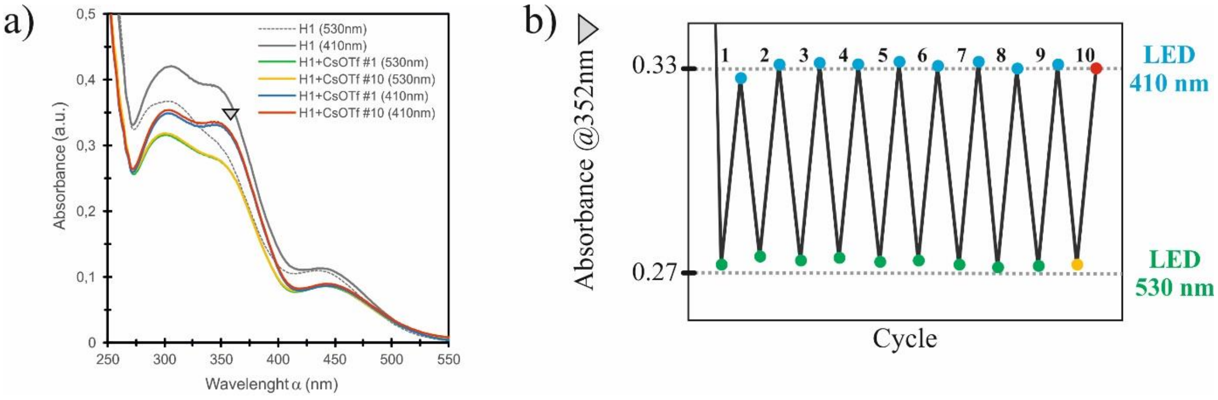

Fig. S7. Photoswitching between complexes of *trans*-1 and *cis*-1 with CsOTf (100 equiv) in MeCN at 298.0±0.1K using green light (530 nm) and blue light (410 nm), respectively.

4. Titration experiments

4.1. General remarks.

Commercially available triflate (TfO) salts (LiOTf, NaOTf, and KOTf) were used as received. CsOTf, (S)-1-PEA, and (R)-PEA salts were prepared as described in Sections 2.11, 2.9, and 2.10, respectively. The titration experiments were conducted as described previously.[3] A simultaneous nonlinear curve fitting for all protons belonging to the receptor and TBA salts, and assuming 1:1 binding model for both *trans*-**1** and *cis*-**1**, was carried out with the HypNMR 2008 software.[4] This procedure provides more reliable K_a's (so-called global association constant) than fitting of the experimental data to a single chemical signal changes.[5] Determination of K_a's for *cis*-**1** was carried out using fully relaxed values of K_a's for *trans*-**1** which were generally similar, i.e. within experimental error, to those obtained during titration of pure *trans*-**1** in the dark. However, when the fitting algorithm failed to converge, fixed values of K_a's for *trans*-**1** were instead used to determine K_a for *cis*-**1**. The details of the titration experiments are given in Table S2.

Table S2. Titration details, global stability constants K_a (M^{-1}), and selected maximum signal shifts ($\Delta\delta_{max}$) of anomeric CH(1) proton for *trans*-**1** and *cis*-**1** with various cations in MeCN-d_3 at 303 K[a]

Host	Guest[b]	c_{Host} (M)	c_{Guest} (M)	K_a (M^{-1})	K_{cis}/K_{trans}	$\Delta\delta_{max,CH(1)}$ (ppm)
trans-**1**[c]	Li^+	0.0089	0.3461	36.8±0.7	0.57±0.05	-0.05
cis-**1**[d]		0.0101	0.4134	20.8±3.4		0.03
trans-**1**[c]	Na^+	0.0089	0.3441	210.5±1.8	2.12±0.03	-0.68
cis-**1**[d]		0.0101	0.3386	446.6±6.8		0.24
trans-**1**[c]	K^+	0.0089	0.2627	1023.3±12.5	2.71±0.06	-1.02
cis-**1**[d]		0.0089	0.2629	2776.5±86.3		0.29
trans-**1**[c]	Cs^+	0.0101	0.4551	90.4±1.0	4.10±0.05	-0.11
cis-**1**[d]		0.0101	0.4480	370.3±5.0		0.05
trans-**1**[c]	(S)-$PEAH^+$	0.0089	0.2149	19.4±0.6	2.00±0.04	-0.18
cis-**1**[d]		0.0089	0.7398	38.8±0.2		-0.24
trans-**1**[c]	(R)-$PEAH^+$	0.0089	0.2096	21.8±0.6	1.95±0.05	-0.24
cis-**1**[d]		0.0092	0.7398	42.5±1.0		-0.38

[a] Determined by ^1H NMR titration experiments and nonlinear curve fitting using HypNMR 2008 software;[4c] for host *cis*-**1** total host concentration is given for a mixture of *trans*- and *cis*-isomers; [b] cations added as triflate (TfO) salts; [c] titration carried out in dark; [d] titration conducted immediately after irradiation with green light (LED 3W, λ = 530 nm).

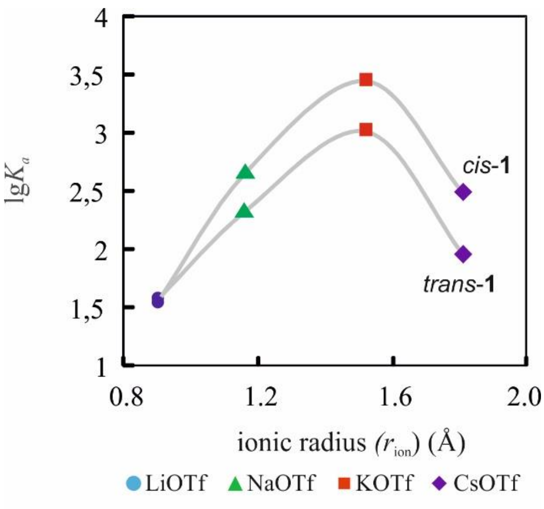

Fig. S8. Dependence of the stability of the complex (lgK_a) on the ionic radius (r_{ion}) of alkali metal cations (added as trifalte salt).

4.2. Titration spectra

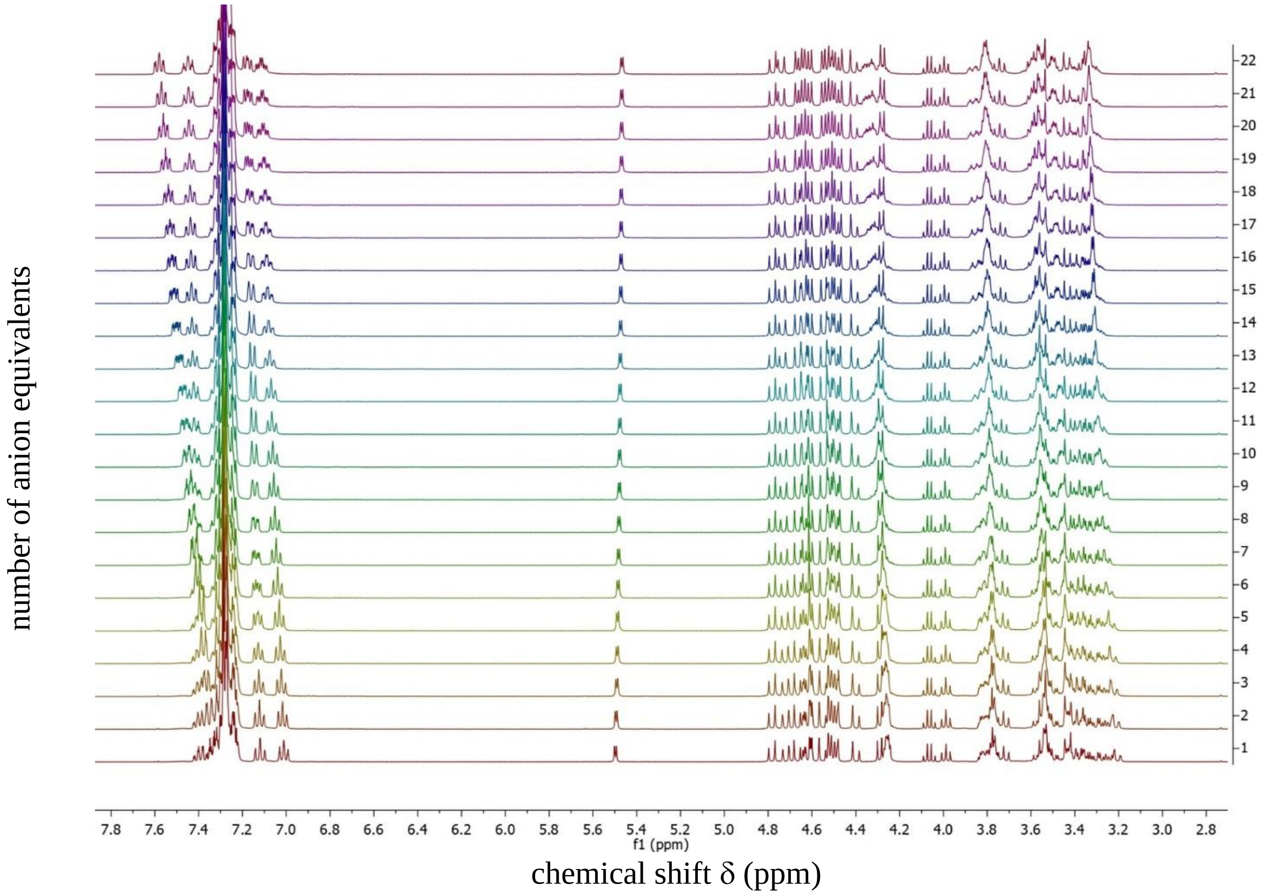

Fig. S9. Stacked plot from ^{1}H NMR titration of *trans*-**1** with increasing amount of LiOTf (from bottom-to-top: 0.00, 0.16, 0.31, 0.46, 0.61, 0.91, 1.21, 1.50, 1.79, 2.07, 2.35, 2.62, 3.16, 3.68, 4.18, 4.67, 5.15, 5.61, 6.51, 7.35, 8.15, 9.28)

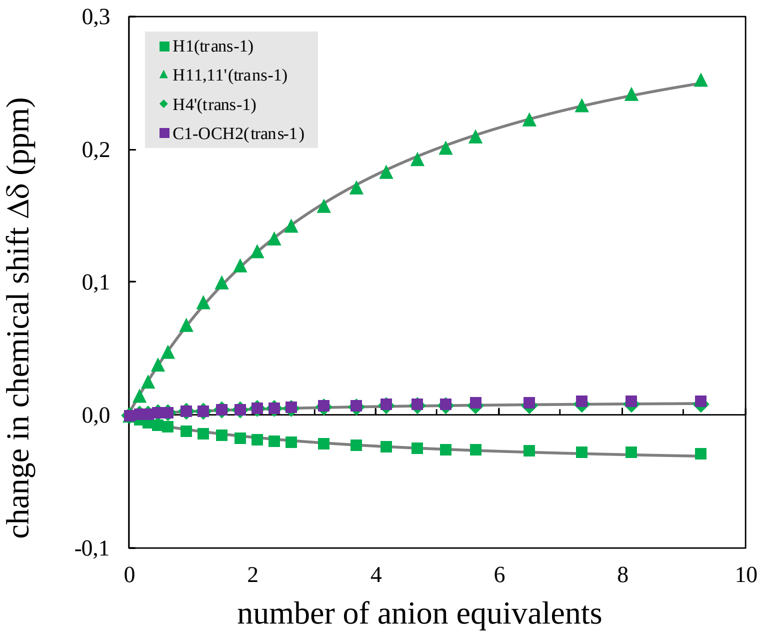

Fig. S10. Experimental chemical shift changes (symbols) and calculated binding isotherms (gray lines) for titration of *trans*-**1** with LiOTf assuming 1:1 binding model; for proton labels see Section 2.7.

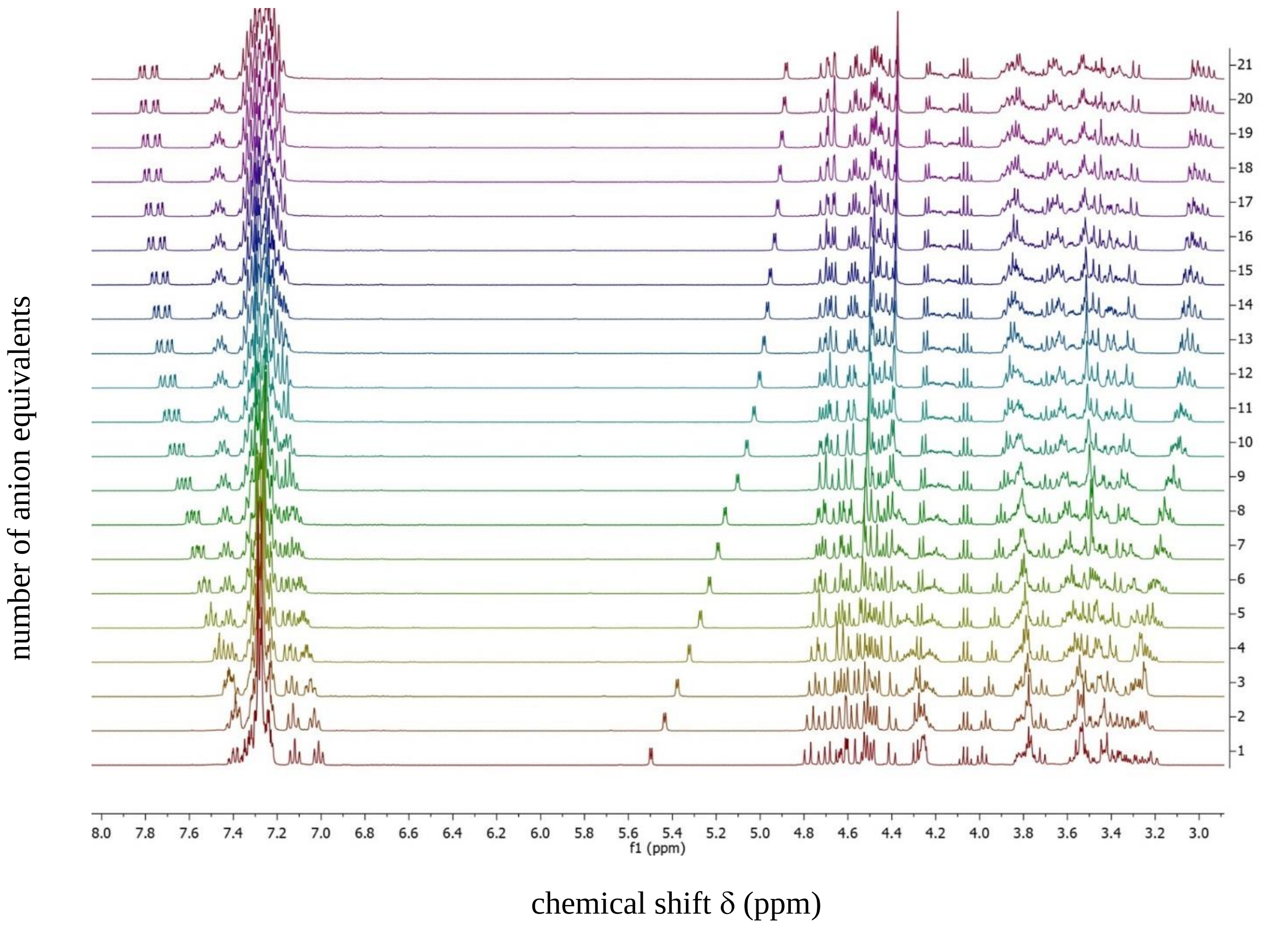

Fig. S11. Stacked plot from ^{1}H NMR titration of *trans*-**1** with increasing amount of NaOTf (from bottom-to-top: 0.00, 0.15, 0.31, 0.46, 0.61, 0.76, 0.91, 1.06, 1.35, 1.64, 1.92, 2.20, 2.47, 2.74, 3.01, 3.53, 4.03, 4.52, 5.00, 5.92, 6.79).

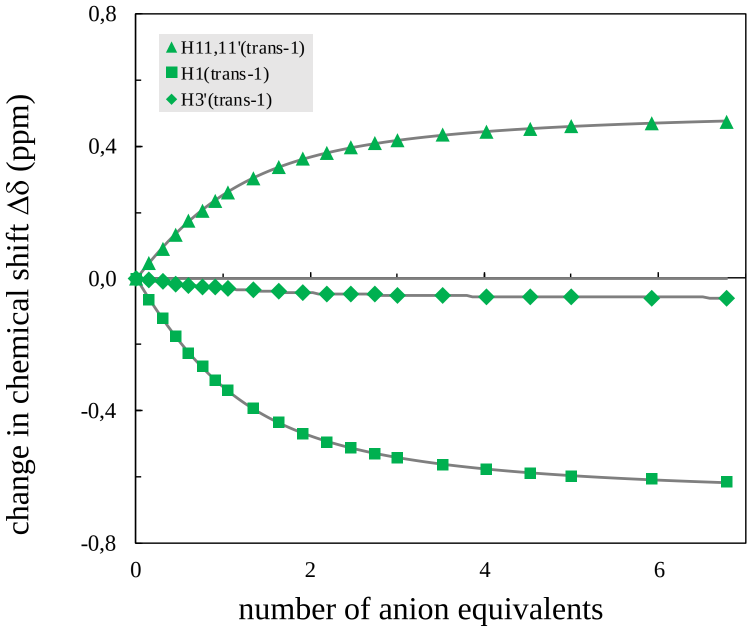

Fig. S12. Experimental chemical shift changes (symbols) and calculated binding isotherms (gray lines) for titration of *trans*-**1** with NaOTf assuming 1:1 binding model; for proton labels see Section 2.7.

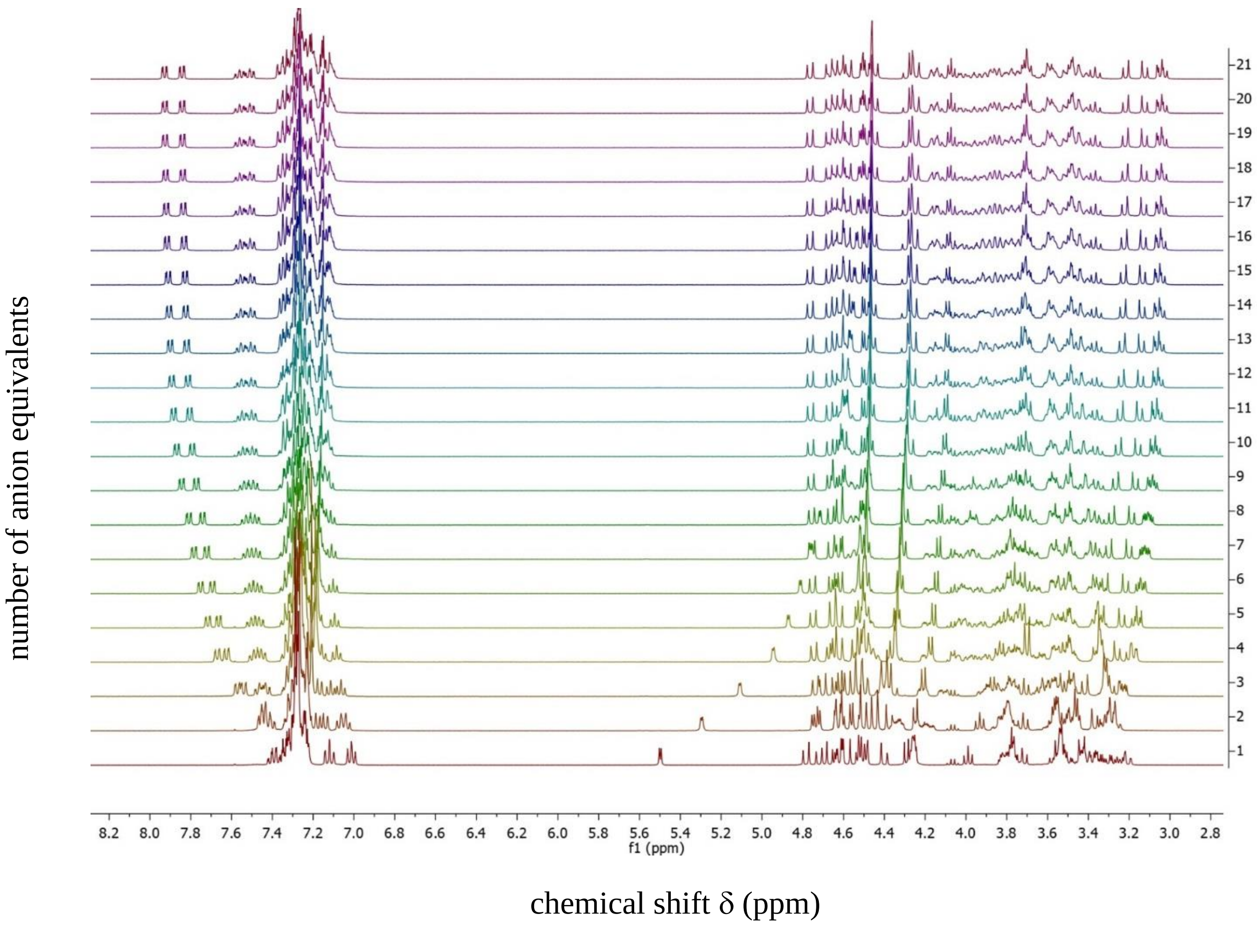

Fig. **S13.** Stacked plot from ^{1}H NMR titration of *trans*-**1** with increasing amount of KOTf (from bottom-to-top: 0.00, 0.23, 0.47, 0.69, 0.81, 0.92, 1.03, 1.14, 1.35, 1.57, 1.78, 1.99, 2.19, 2.39, 2.59, 2.98, 3.35, 3.72, 4.08, 4.42, 4.76).

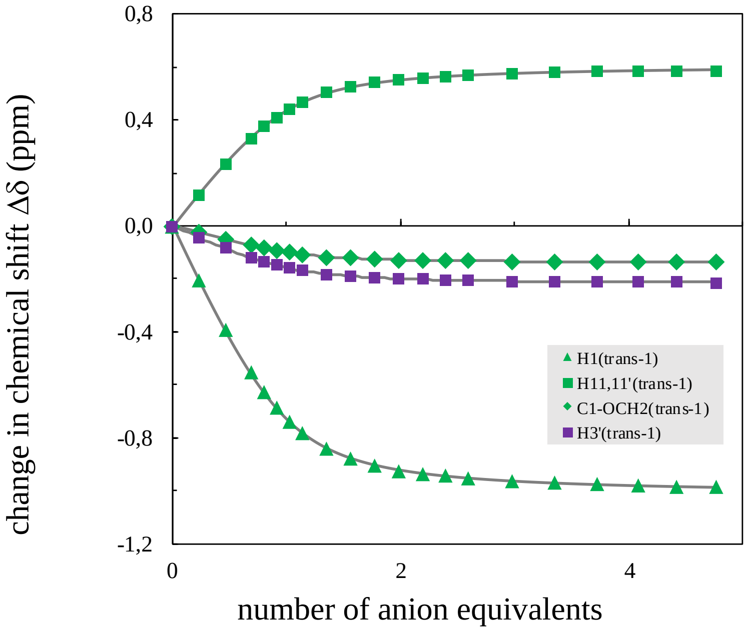

Fig. S14. Experimental chemical shift changes (symbols) and calculated binding isotherms (gray lines) for titration of *trans*-**1** with KOTf assuming 1:1 binding model; for proton labels see Section 2.7.

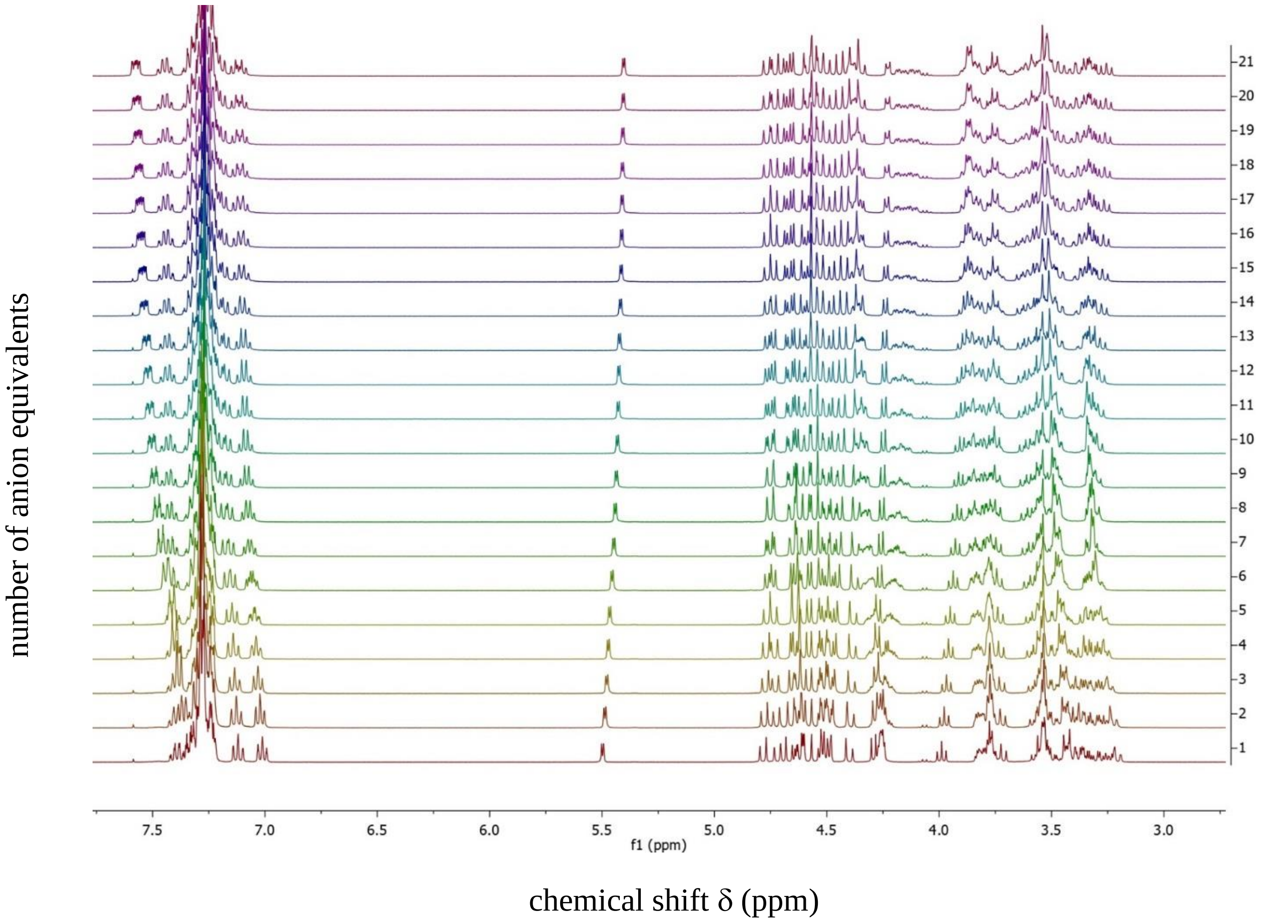

Fig. **S15.** Stacked plot from ^{1}H NMR titration of *trans*-**1** with increasing amount of CsOTf (from bottom-to-top: 0.00, 0.18, 0.36, 0.53, 0.71, 1.05, 1.39, 1.73, 2.06, 2.39, 2.71, 3.02, 3.33, 3.94, 4.53, 5.10, 5.66, 6.20, 6.73, 7.75, 8.71).

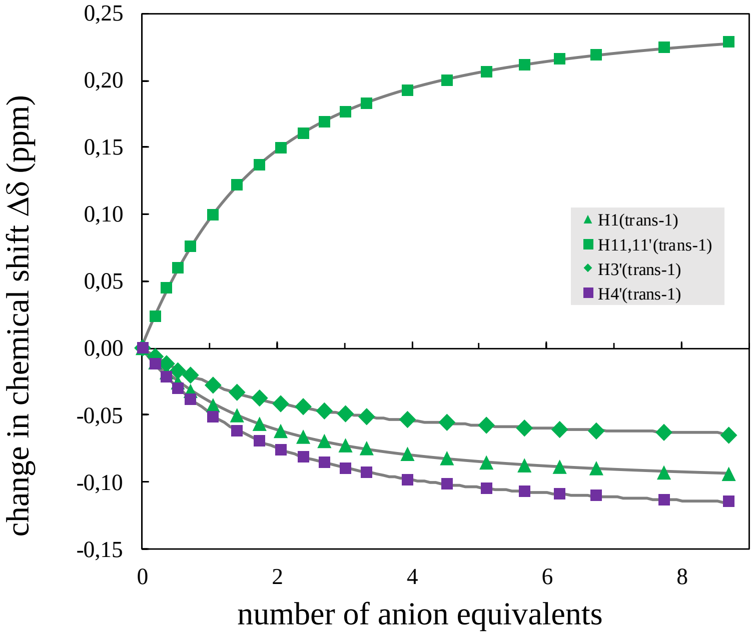

Fig. S16. Experimental chemical shift changes (symbols) and calculated binding isotherms (gray lines) for titration of *trans*-**1** with CsOTf assuming 1:1 binding model; for proton labels see Section 2.7.

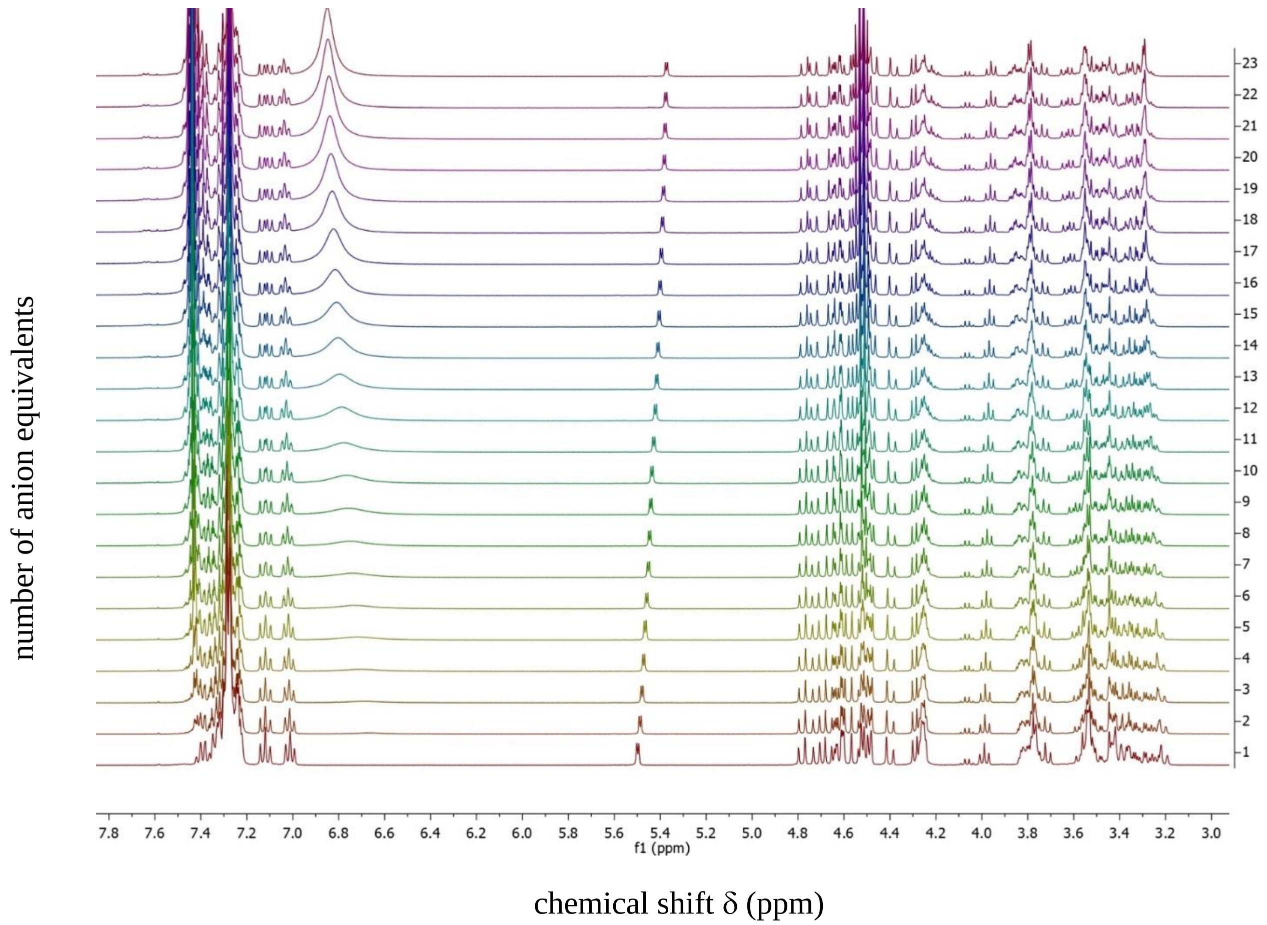

Fig. S17. Stacked plot from ^{1}H NMR titration of *trans*-**1** with increasing amount of (S)-PEA-TfOH (from bottom-to-top: 0.00, 0.19, 0.38, 0.57, 0.75, 0.93, 1.11, 1.45, 1.79, 2.12, 2.44, 2.74, 3.04, 3.62, 4.16, 4.68, 5.17, 5.64, 6.30, 6.91, 7.48, 8.02).

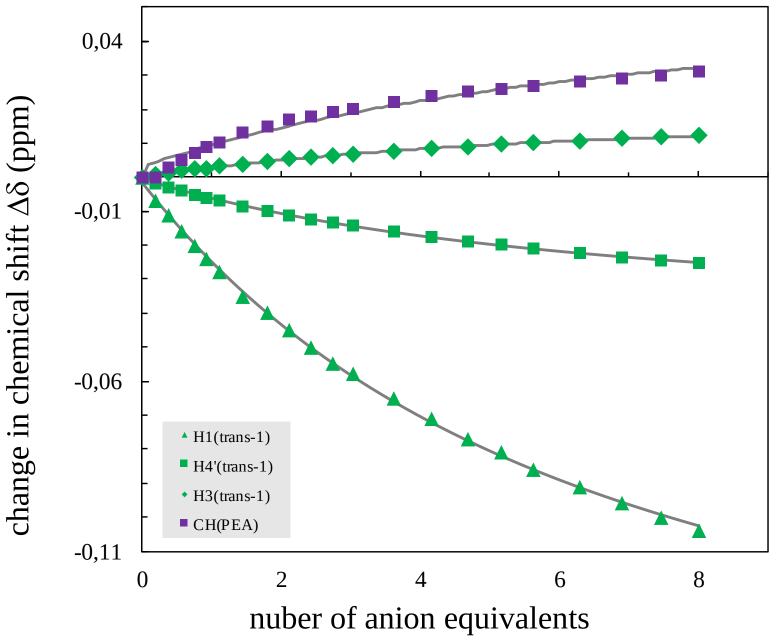

Fig. S18. Experimental chemical shift changes (symbols) and calculated binding isotherms (gray lines) for titration of *trans*-**1** with (S)-PEA-TfOH assuming 1:1 binding model; for proton labels see section 2.7.

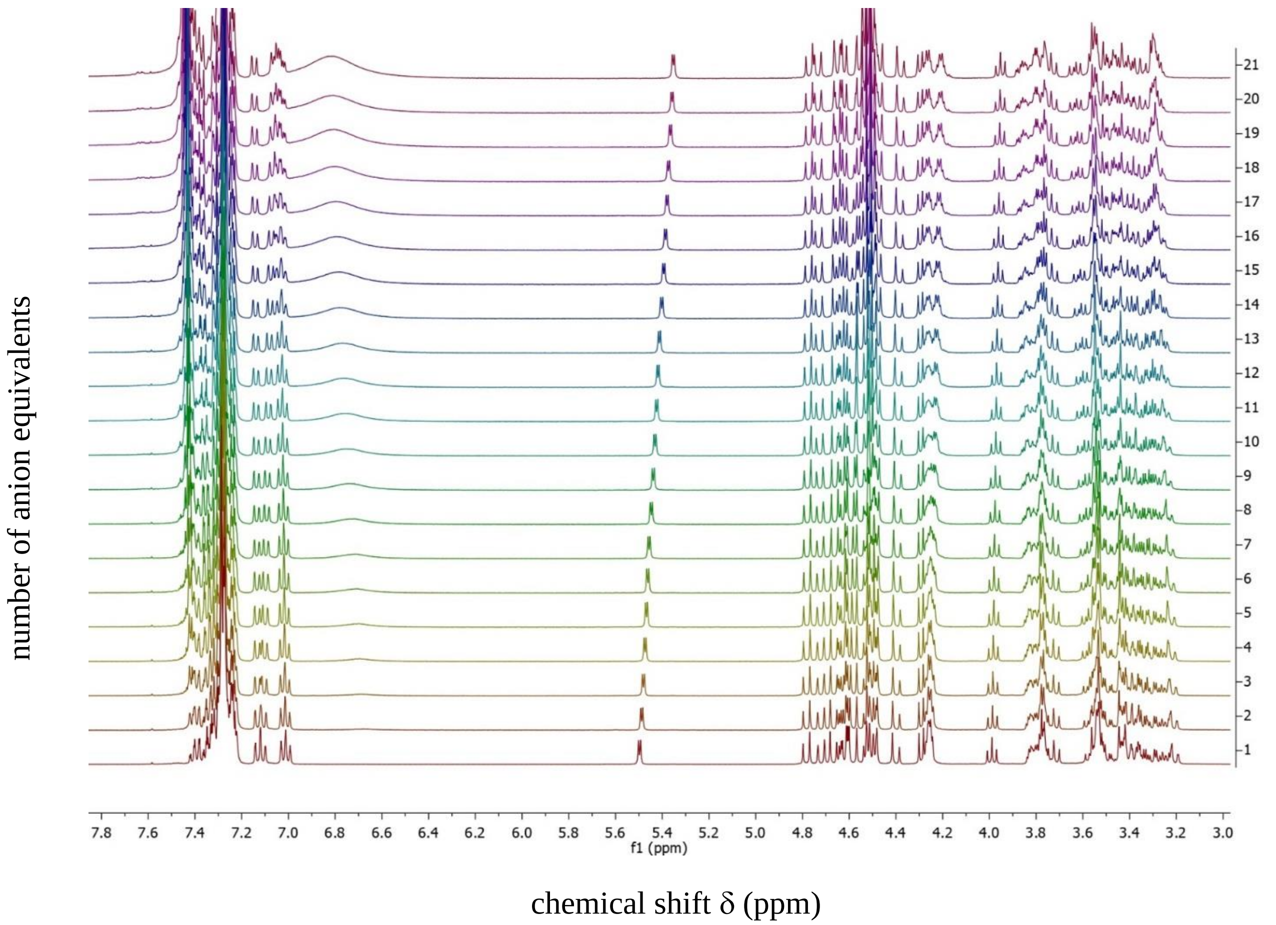

Fig. S19. Stacked plot from ^{1}H NMR titration of *trans*-**1** with increasing amount of (R)-PEA-TfOH (from bottom-to-top: 0.00, 0.19, 0.37, 0.55, 0.73, 0.91, 1.08, 1.42, 1.75, 2.07, 2.38, 2.68, 2.97, 3.53, 4.06, 4.57, 5.04, 5.50, 6.14, 6.74, 7.30).

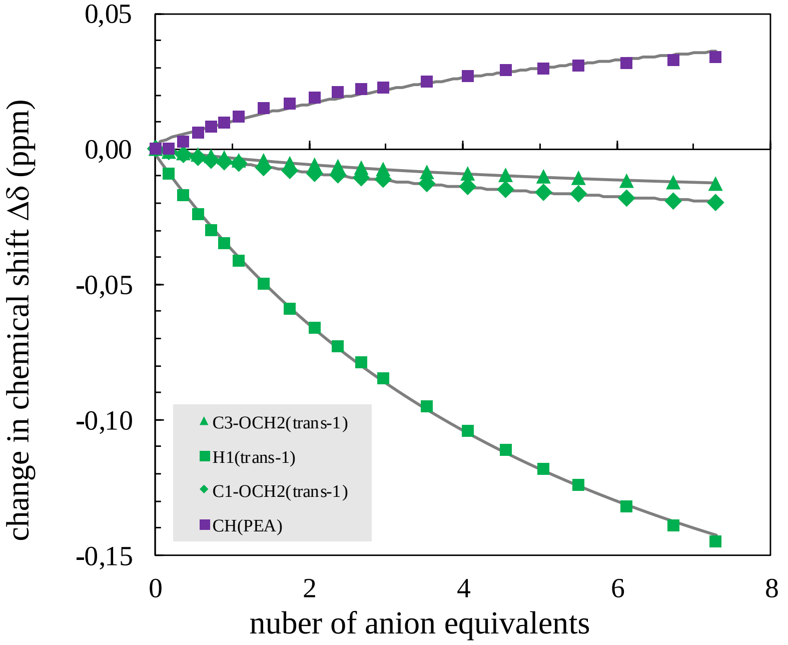

Fig. S20. Experimental chemical shift changes (symbols) and calculated binding isotherms (gray lines) for titration of *trans*-**1** with (R)-PEA-TfOH assuming 1:1 binding model; for proton labels see section 2.7.

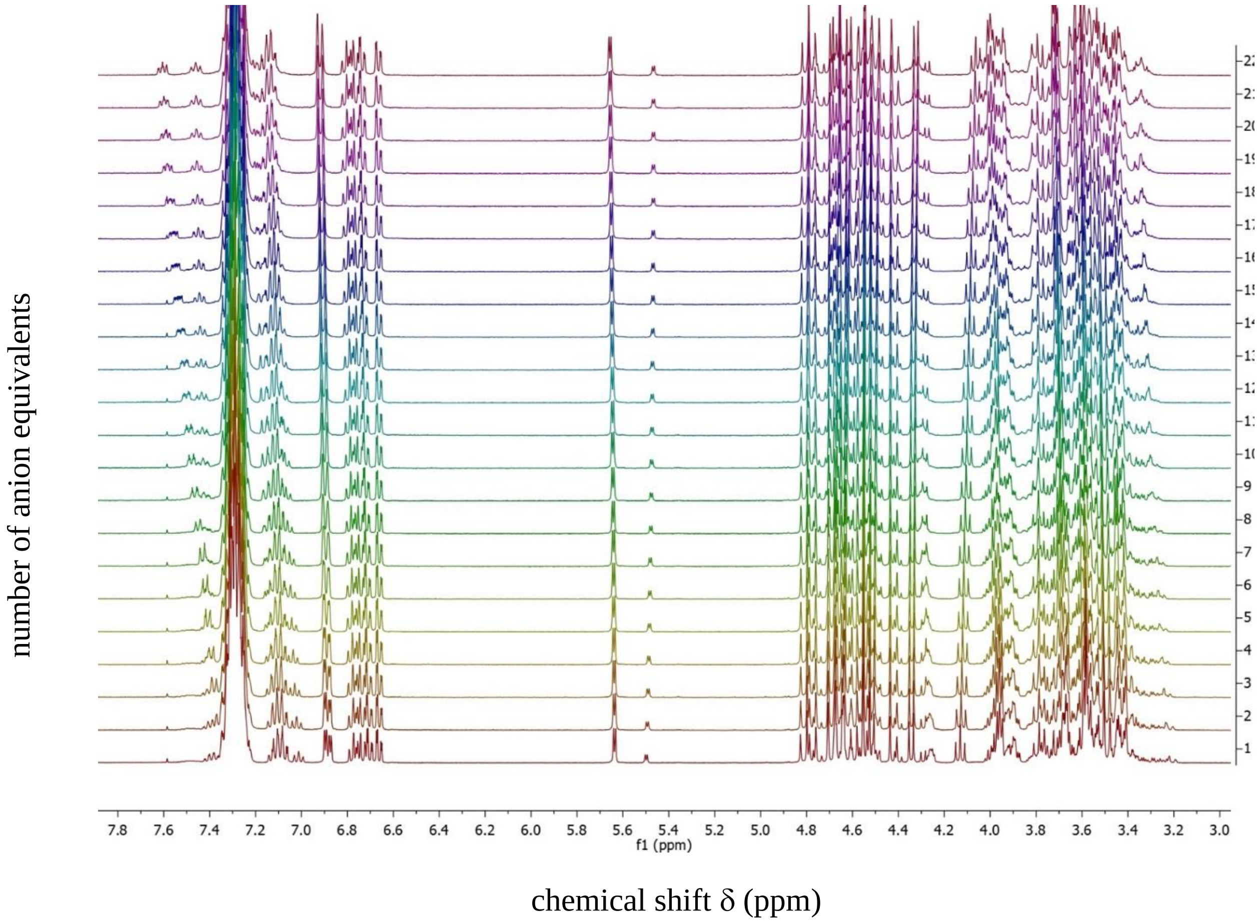

Fig. **S21.** Stacked plot from ^{1}H NMR titration of *cis*-**1** with increasing amount of LiOTf (from bottom-to-top: 0.00, 0.16, 0.32, 0.48, 0.64, 0.80, 0.96, 1.27, 1.57, 1.87, 2.16, 2.46, 2.74, 3.30, 3.85, 4.37, 4.89, 5.87, 6.80, 7.69, 8.53, 9.32).

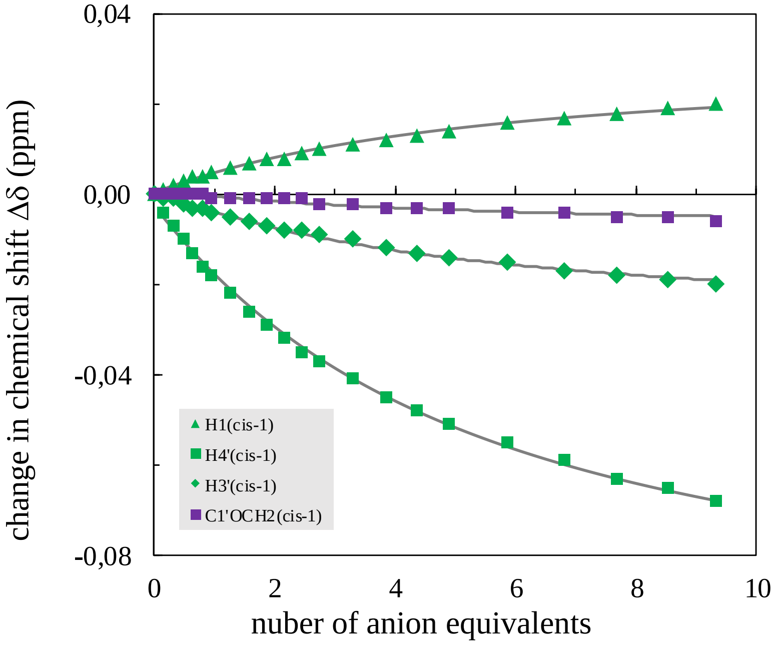

Fig. S22. Experimental chemical shift changes (symbols) and calculated binding isotherms (gray lines) for titration of *cis*-**1** with LiOTf assuming 1:1 binding model; for proton labels see Section 2.8.

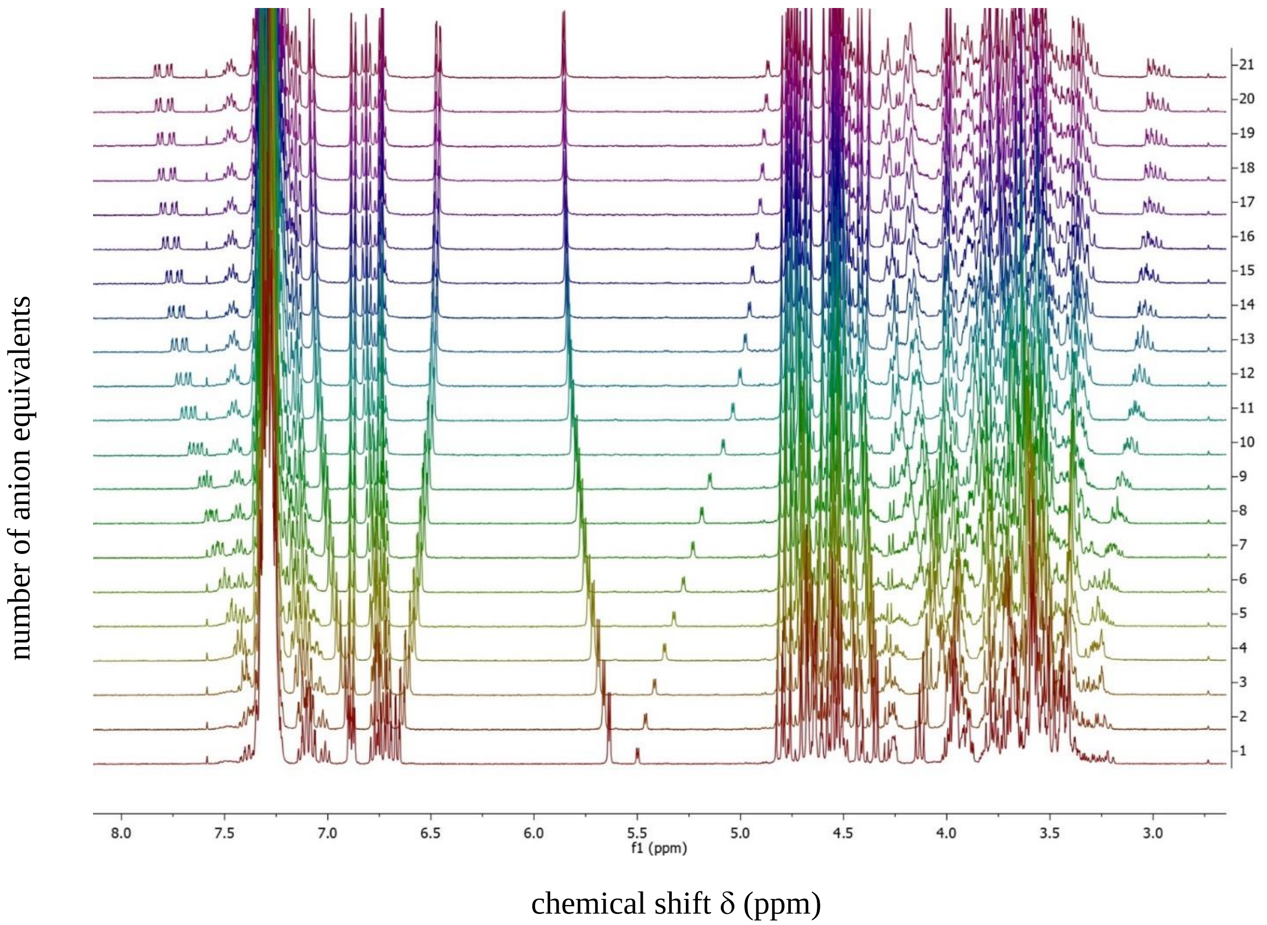

Fig. S23. Stacked plot from ^{1}H NMR titration of *cis*-**1** with increasing amount of NaOTf (from bottom-to-top: 0.00, 0.13, 0.27, 0.40, 0.53, 0.66, 0.78, 0.91, 1.04, 1.29, 1.53, 1.77, 2.01, 2.25, 2.48, 2.93, 3.37, 3.79, 4.21, 5.00, 5.76).

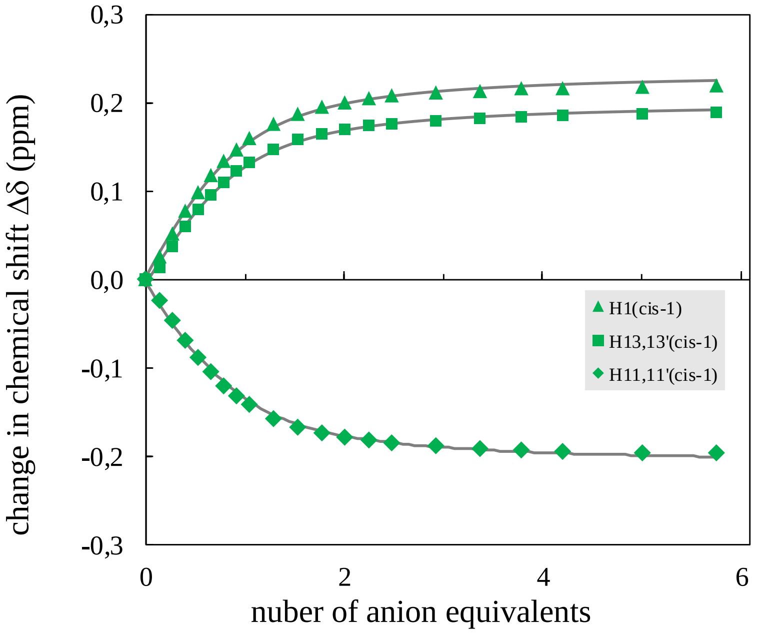

Fig. S24. Experimental chemical shift changes (symbols) and calculated binding isotherms (gray lines) for titration of *cis*-**1** with NaOTf assuming 1:1 binding model; for proton labels see Section 2.8.

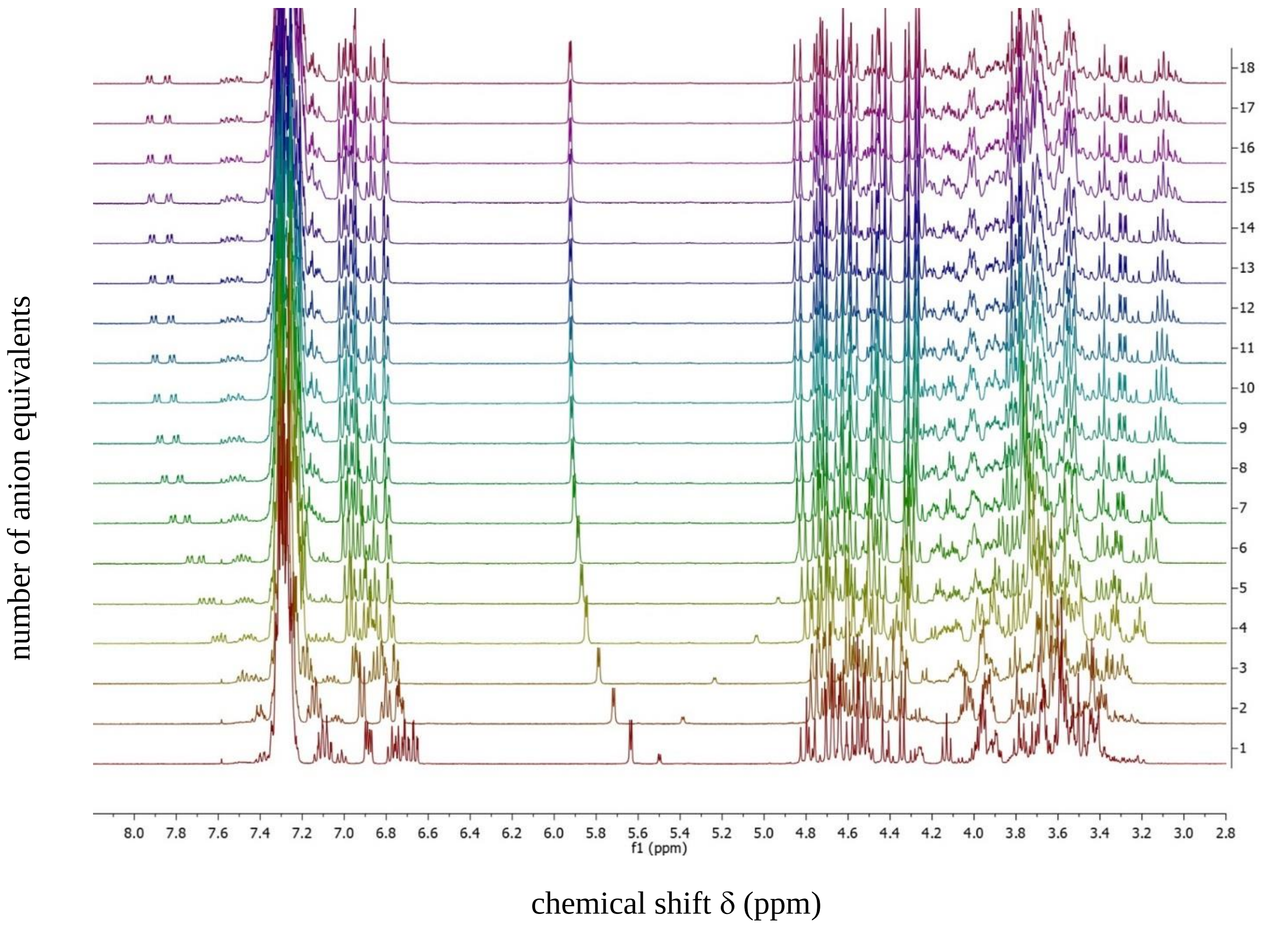

Fig. S25. Stacked plot from ^{1}H NMR titration of *cis*-**1** with increasing amount of KOTf (from bottom-to-top: 0.00, 0.24, 0.47, 0.69, 0.81, 0.92, 1.14, 1.36, 1.57, 1.78, 1.99, 2.20, 2.40, 2.60, 2.99, 3.36, 3.73, 4.09).

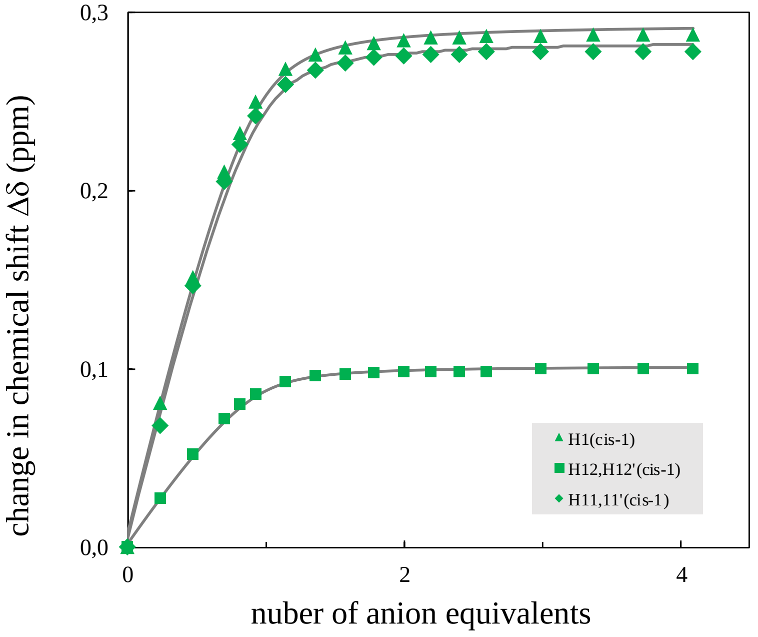

Fig. S26. Experimental chemical shift changes (symbols) and calculated binding isotherms (gray lines) for titration of *cis*-**1** with KOTf assuming 1:1 binding model; for proton labels see Section 2.8.

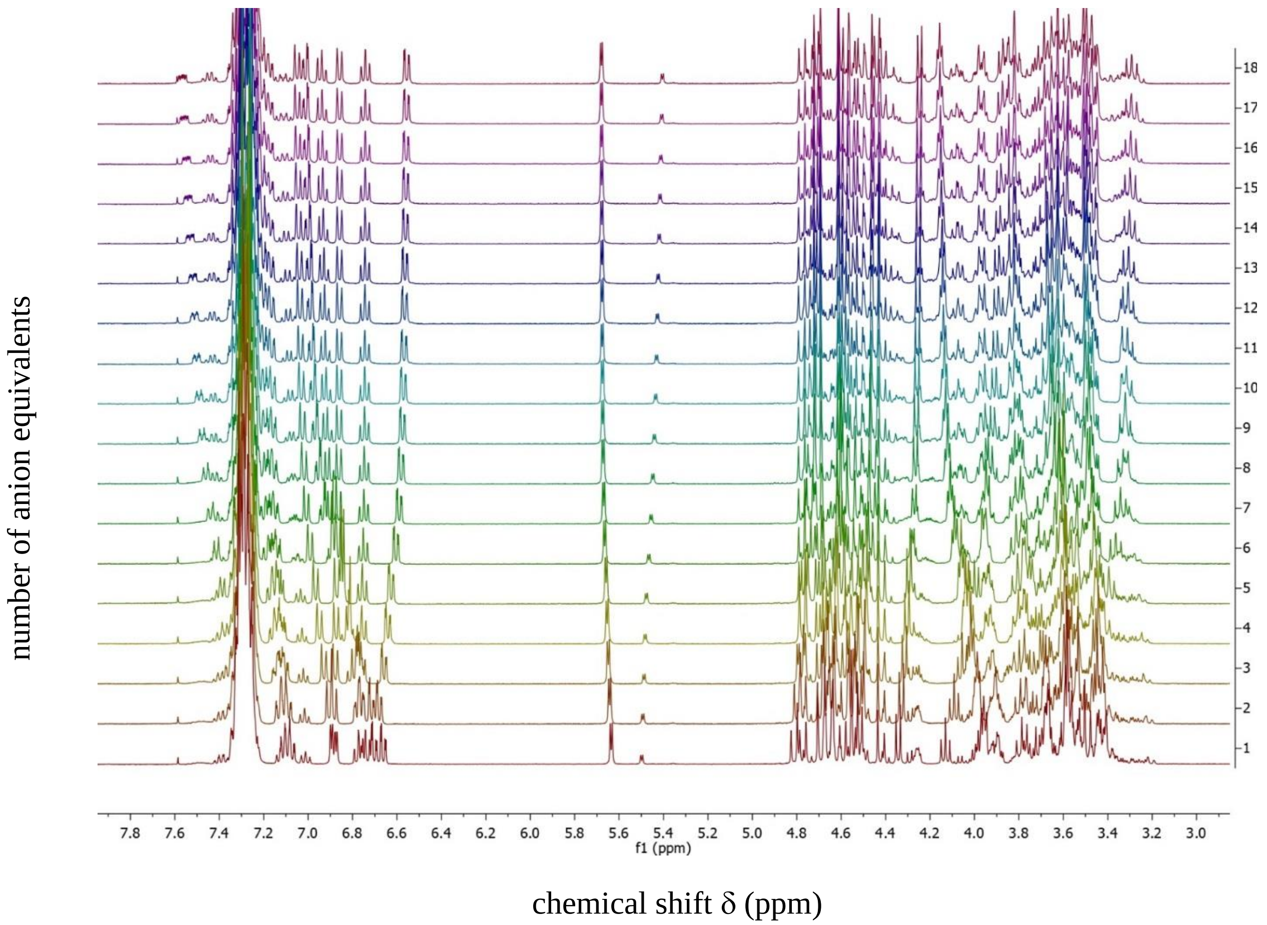

Fig. S27. Stacked plot from ^{1}H NMR titration of *cis*-**1** with increasing amount of CsOTf (from bottom-to-top: 0.00, 0.18, 0.35, 0.53, 0.70, 1.04, 1.37, 1.70, 2.03, 2.35, 2.66, 2.97, 3.28, 3.88, 4.46, 5.03, 6.11, 7.13).

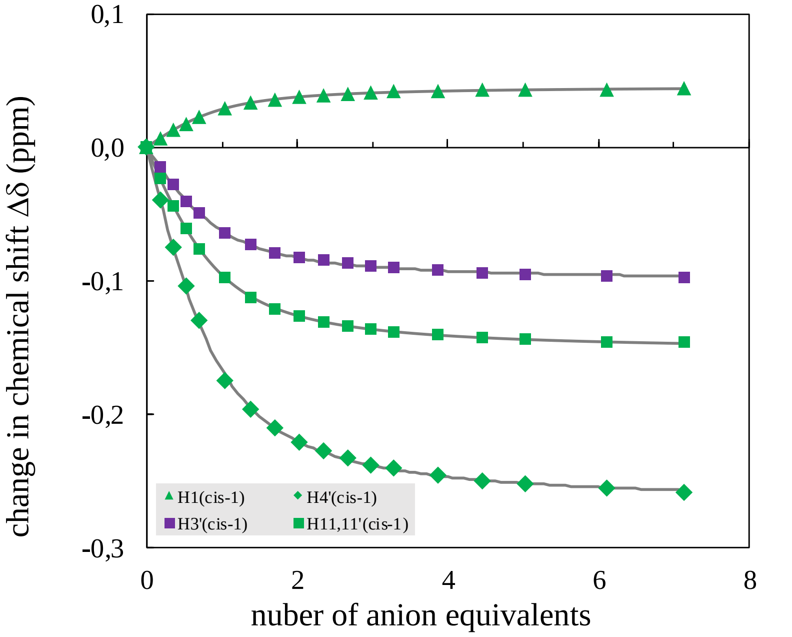

Fig. S28. Experimental chemical shift changes (symbols) and calculated binding isotherms (gray lines) for titration of *cis*-**1** with CsOTf assuming 1:1 binding model; for proton labels see Section 2.8.

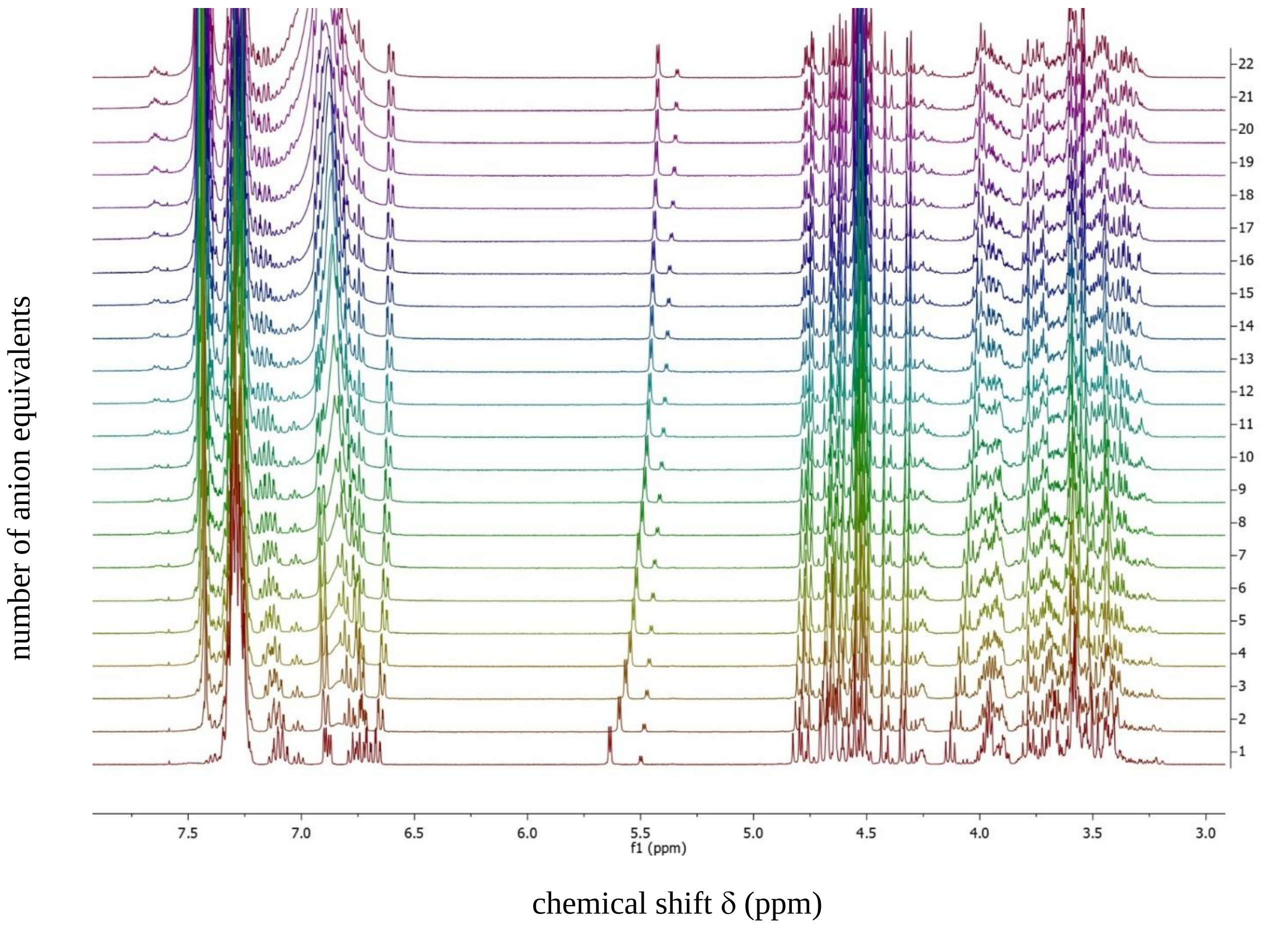

Fig. S29. Stacked plot from ^{1}H NMR titration of *cis*-**1** with increasing amount of (S)-PEA-TfOH (from bottom-to-top: 0.00, 0.66, 1.31, 1.96, 2.59, 3.21, 3.82, 5.02, 6.18, 7.31, 8.40, 9.47, 10.50, 11.51, 12.49, 13.44, 15.27, 17.00, 18.65, 20.22, 21.72, 23.15).

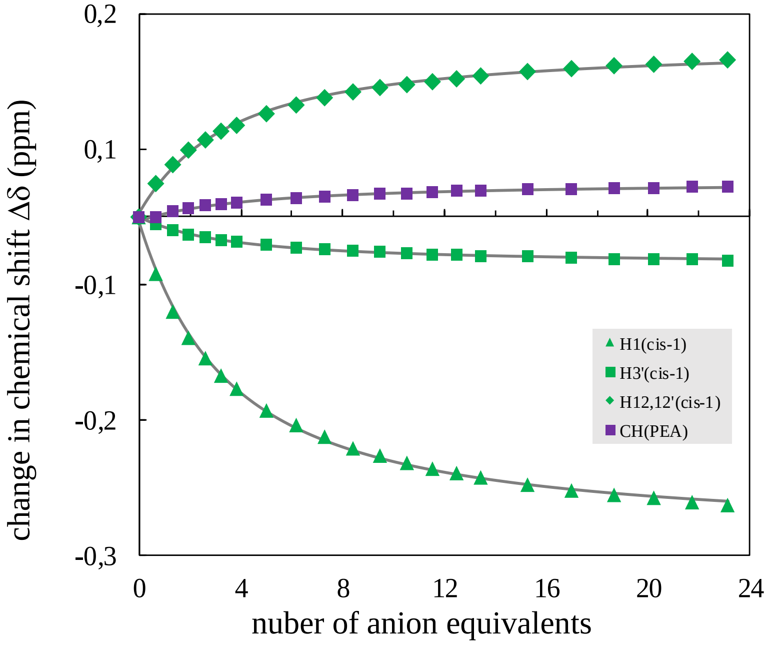

Fig. S30. Experimental chemical shift changes (symbols) and calculated binding isotherms (gray lines) for titration of *cis*-**1** with (S)-PEA-TfOH assuming 1:1 binding model; for proton labels see section 2.8.

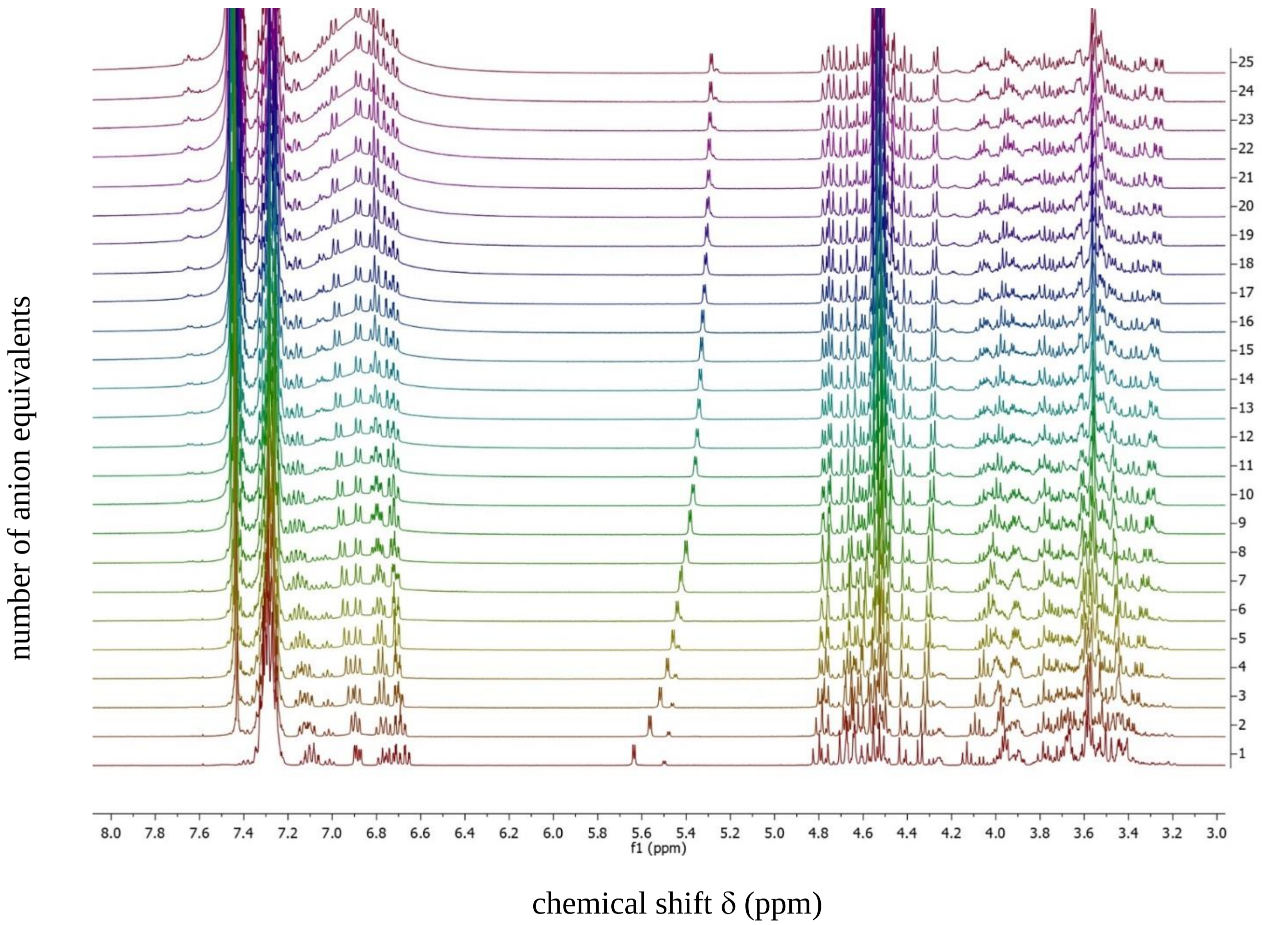

Fig. S31. Stacked plot from ^{1}H NMR titration of *cis*-**1** with increasing amount of (R)-PEA-TfOH (from bottom-to-top: 0.00, 0.64, 1.27, 1.89, 2.51, 3.11, 3.70, 4.86, 5.99, 7.08, 8.14, 9.17, 10.18, 11.15, 12.10, 13.02, 14.79, 16.48, 18.08, 19.60, 21.05, 22.43, 24.39, 26.22, 27.93).

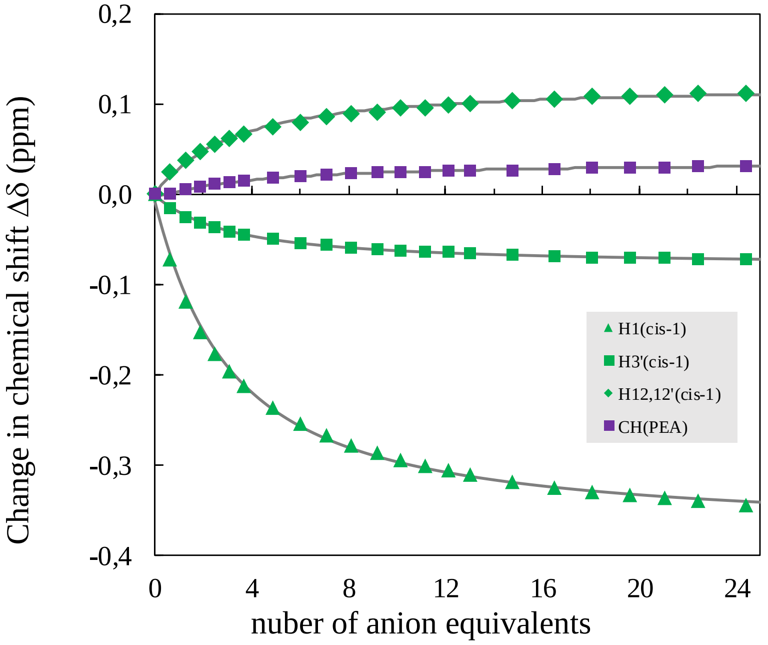

Fig. S32. Experimental chemical shift changes (symbols) and calculated binding isotherms (gray lines) for titration of *cis*-**1** with (R)-PEA-TfOH assuming 1:1 binding model; for proton labels see section 2.8.

5. Copies of the NMR spectra

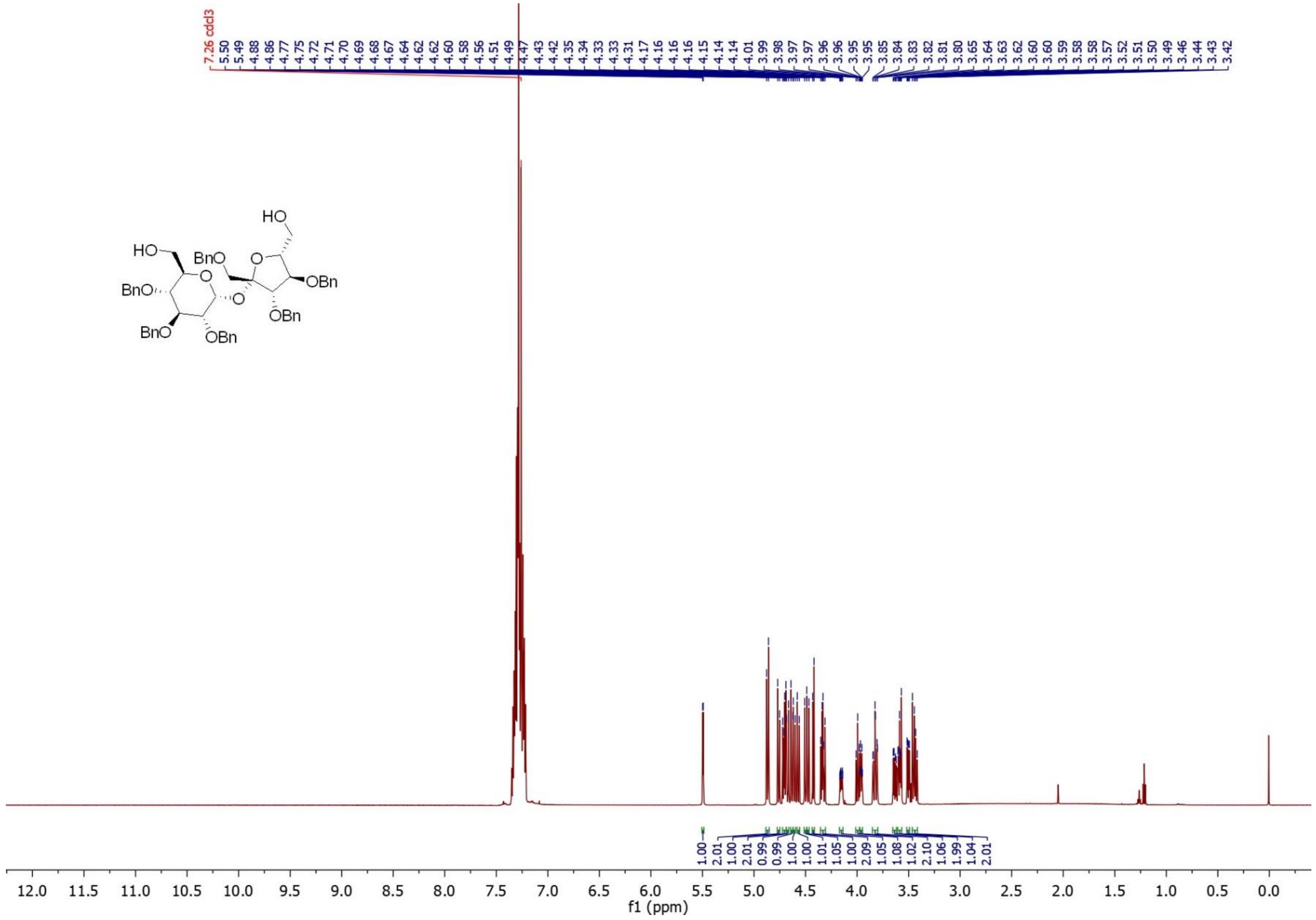

Figure S33. ^{1}H NMR (600 MHz, $CDCl_3$) spectrum of compound **5**.

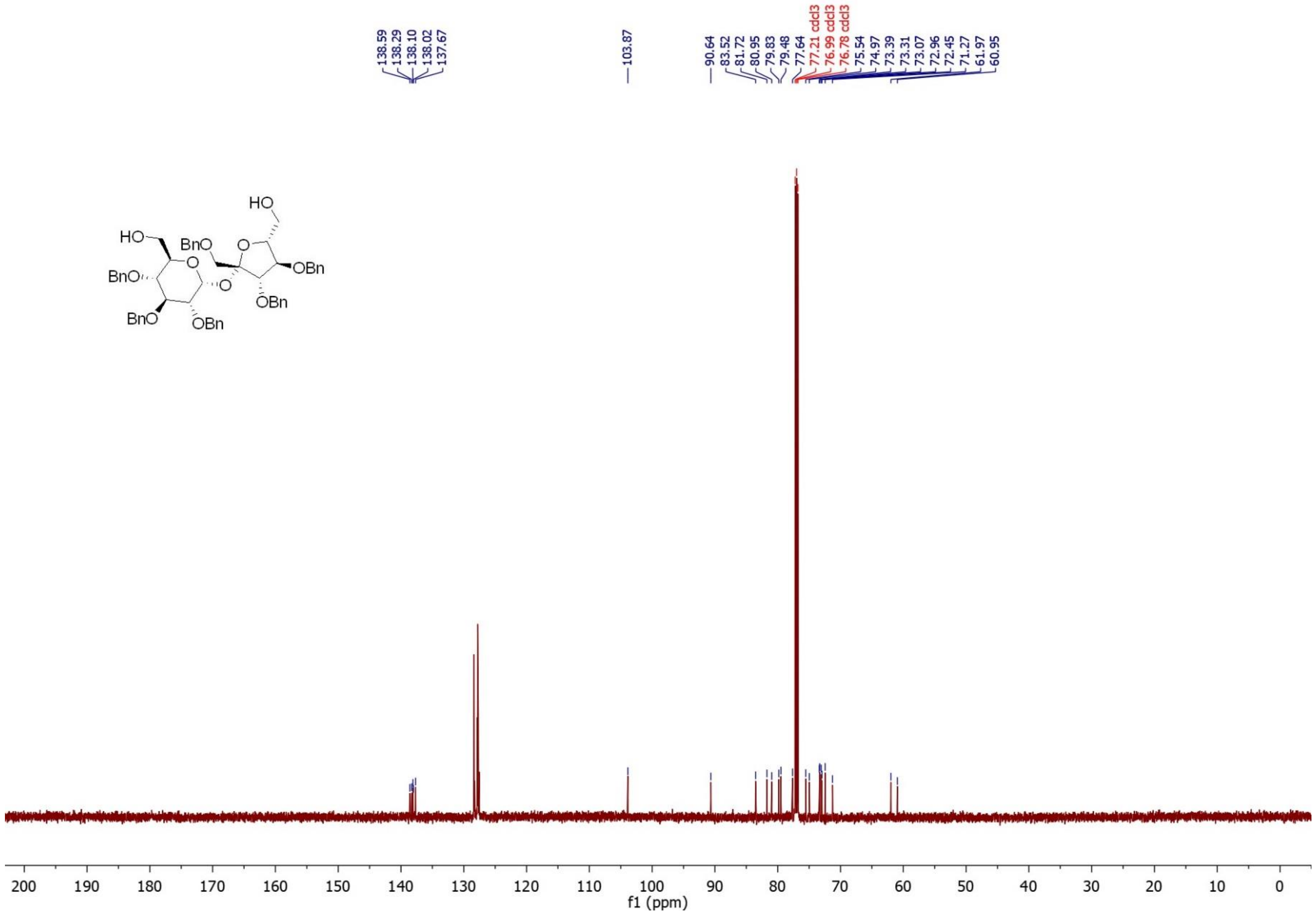

Figure S34. ^{13}C NMR (151 MHz, $CDCl_3$) spectrum of compound **5**.

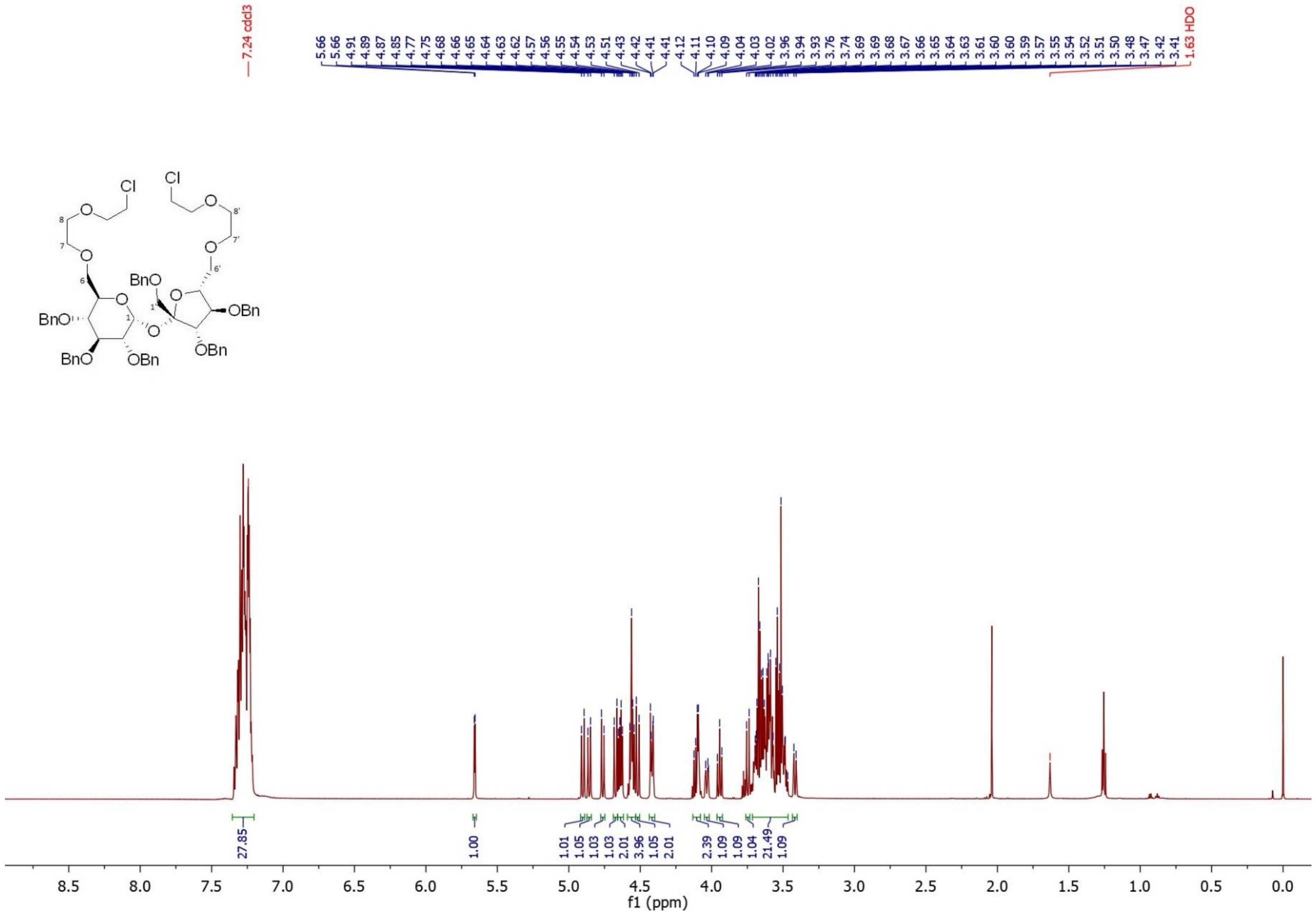

Figure S35. ^{1}H NMR (600 MHz, $CDCl_3$) spectrum of compound **6**.

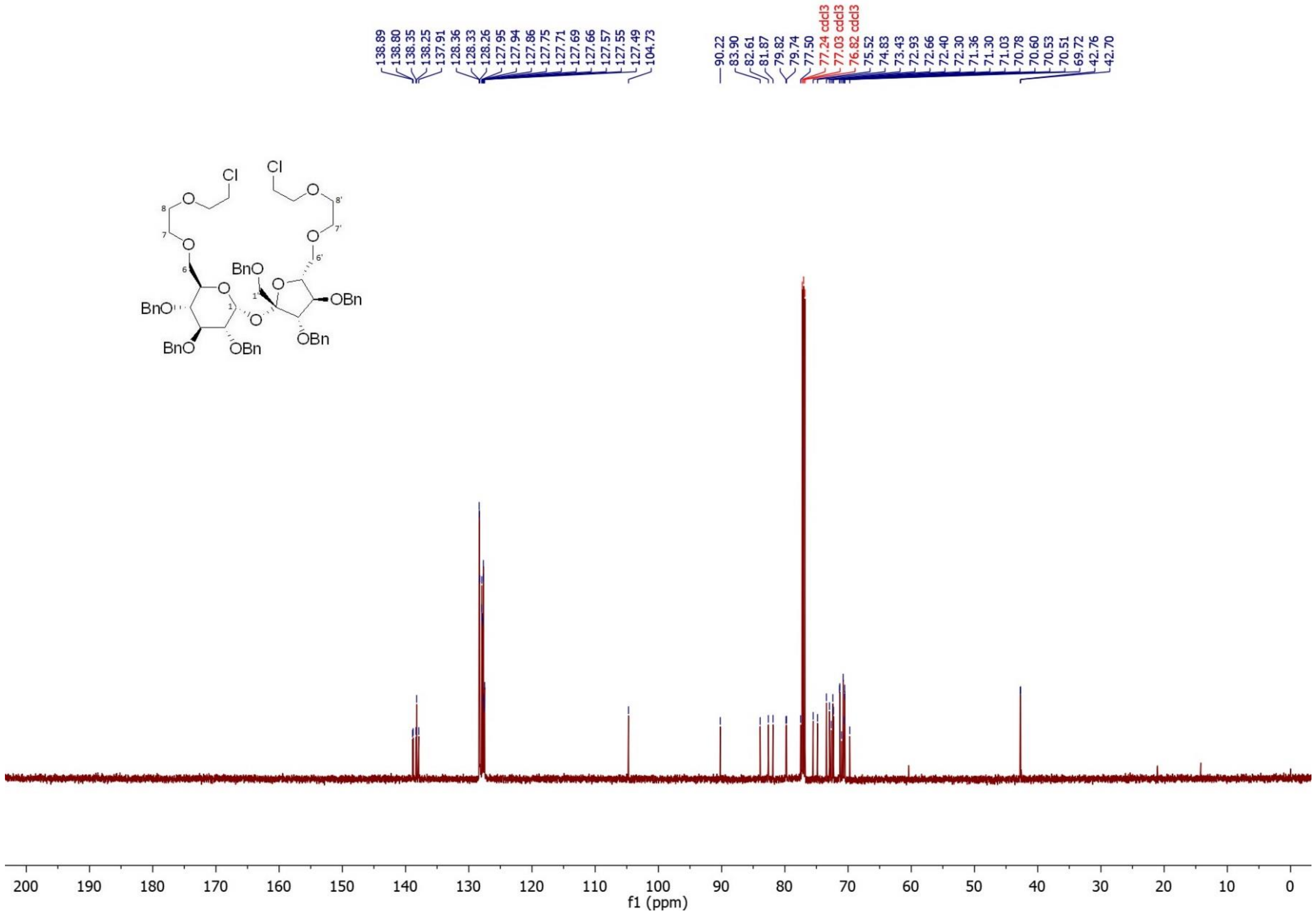

Figure S36. ^{13}C NMR (151 MHz, $CDCl_3$) spectrum of compound **6**.

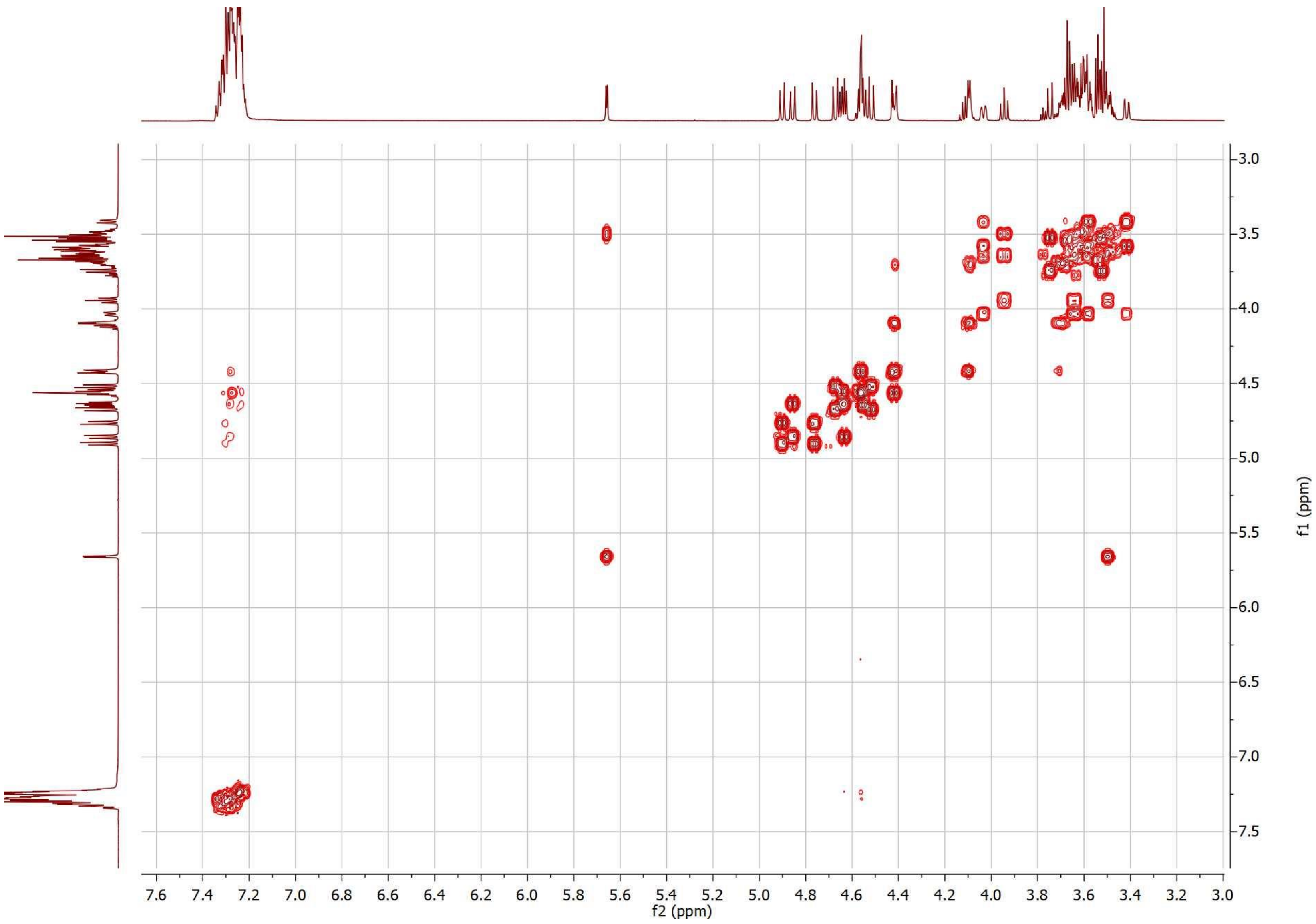

Figure S37. gCOSY spectrum of compound **6**.

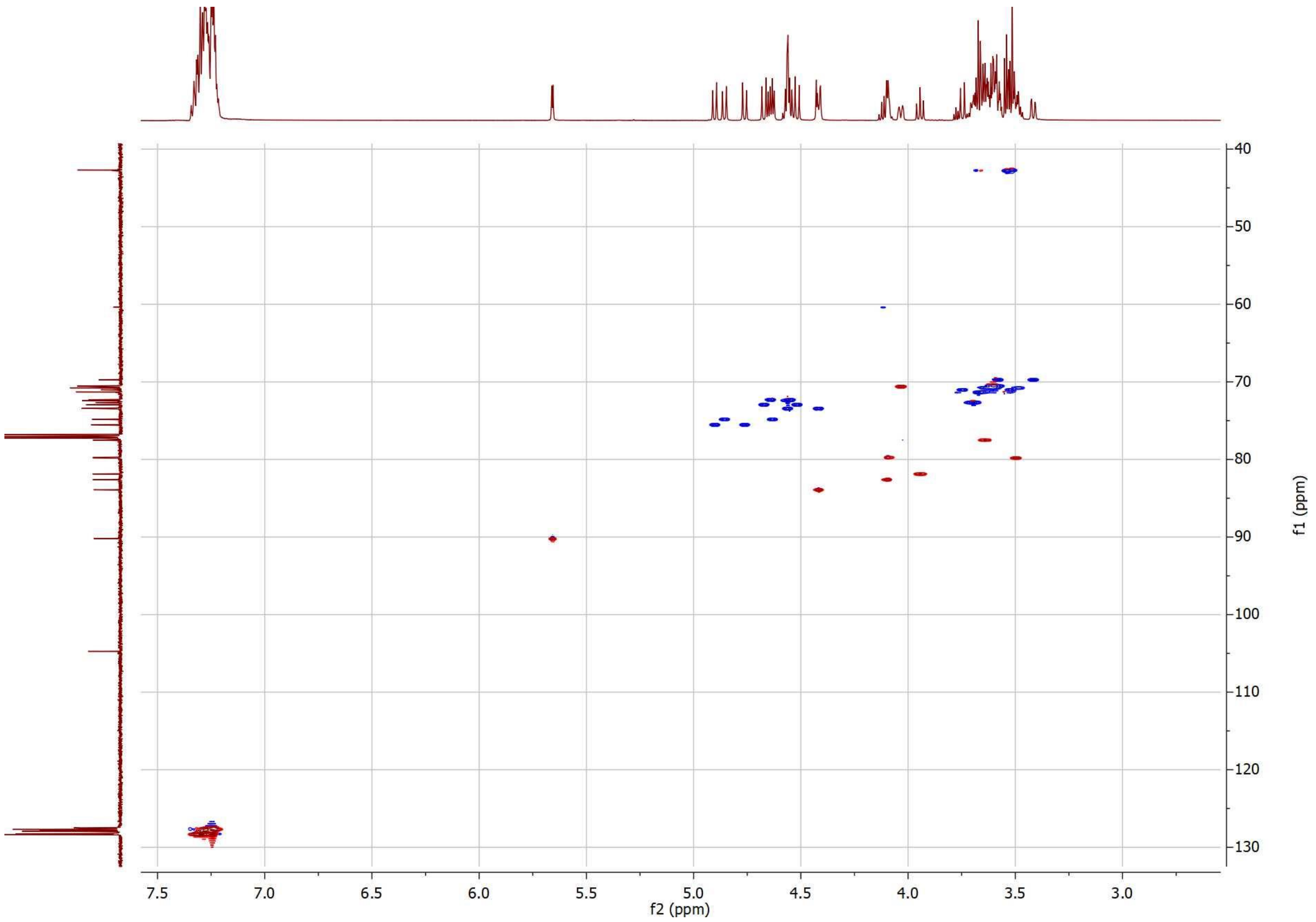

Figure S38. gHSQCAD spectrum of compound **6**.

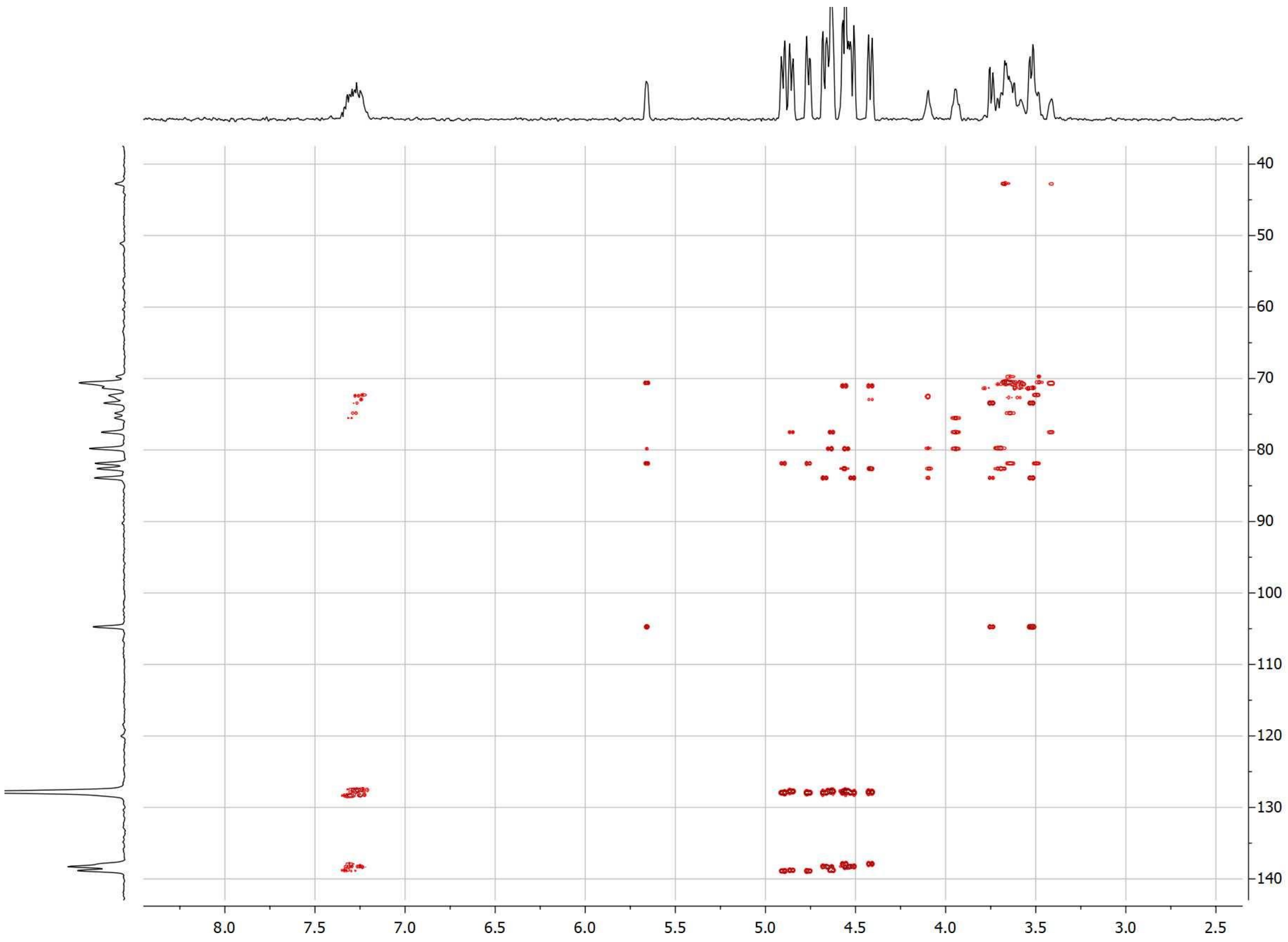

Figure S39. gHMBCAD spectrum of compound **6**.

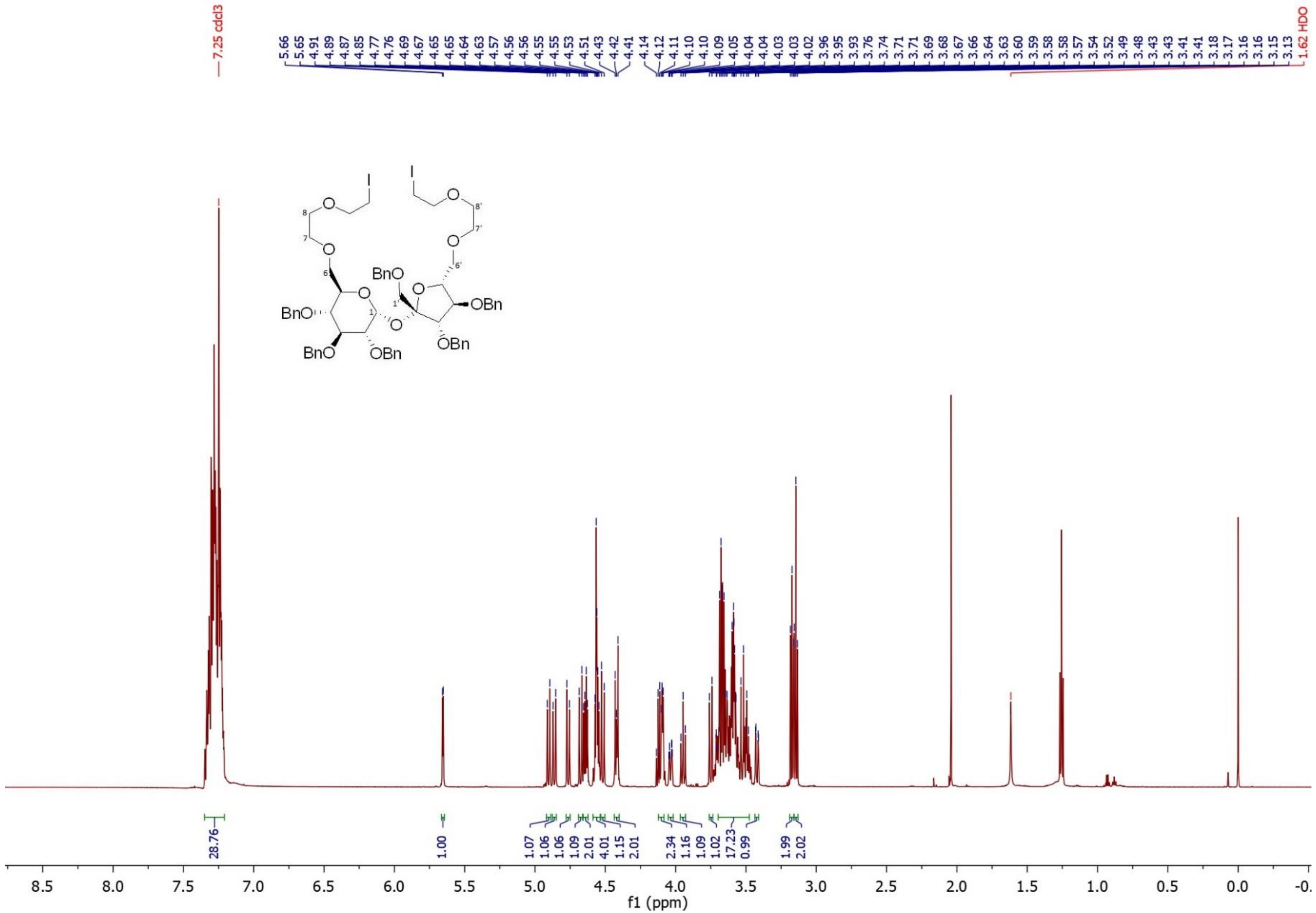

Figure S40. ^{1}H NMR (600 MHz, $CDCl_3$) spectrum of compound **7**.

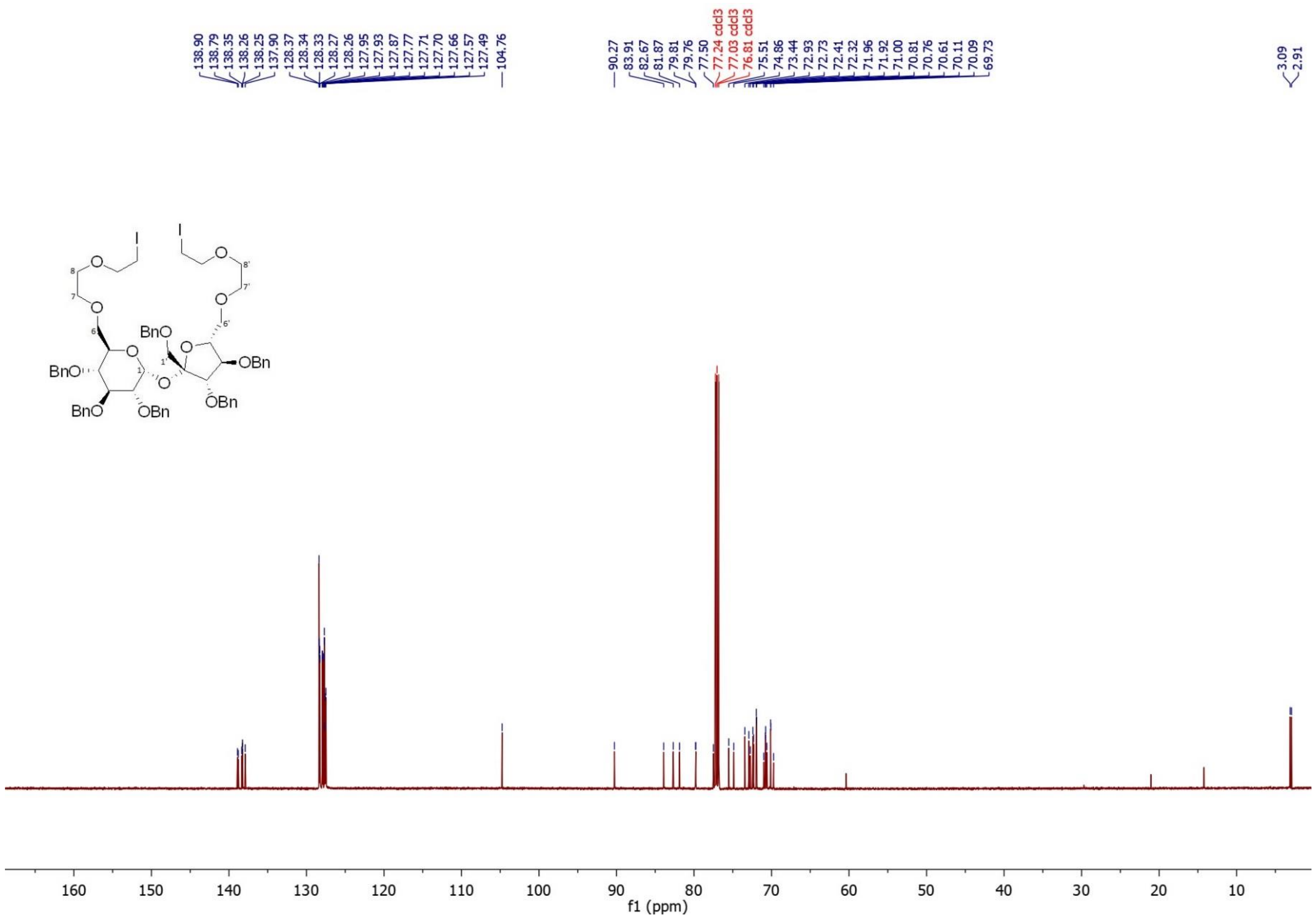

Figure S41. ^{13}C NMR (151 MHz, $CDCl_3$) spectrum of compound **7**.

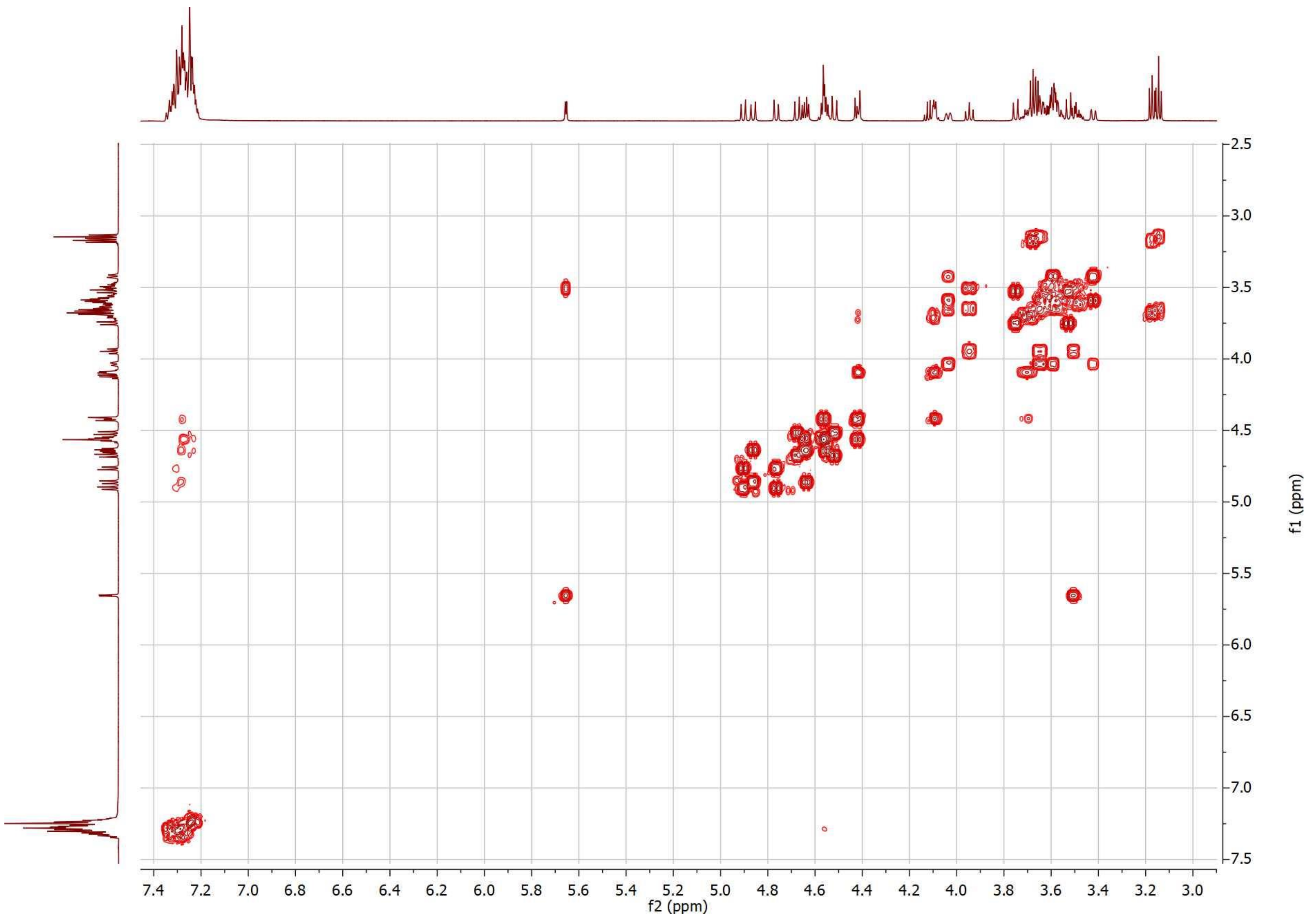

Figure S42. gCOSY spectrum of compound **7**.

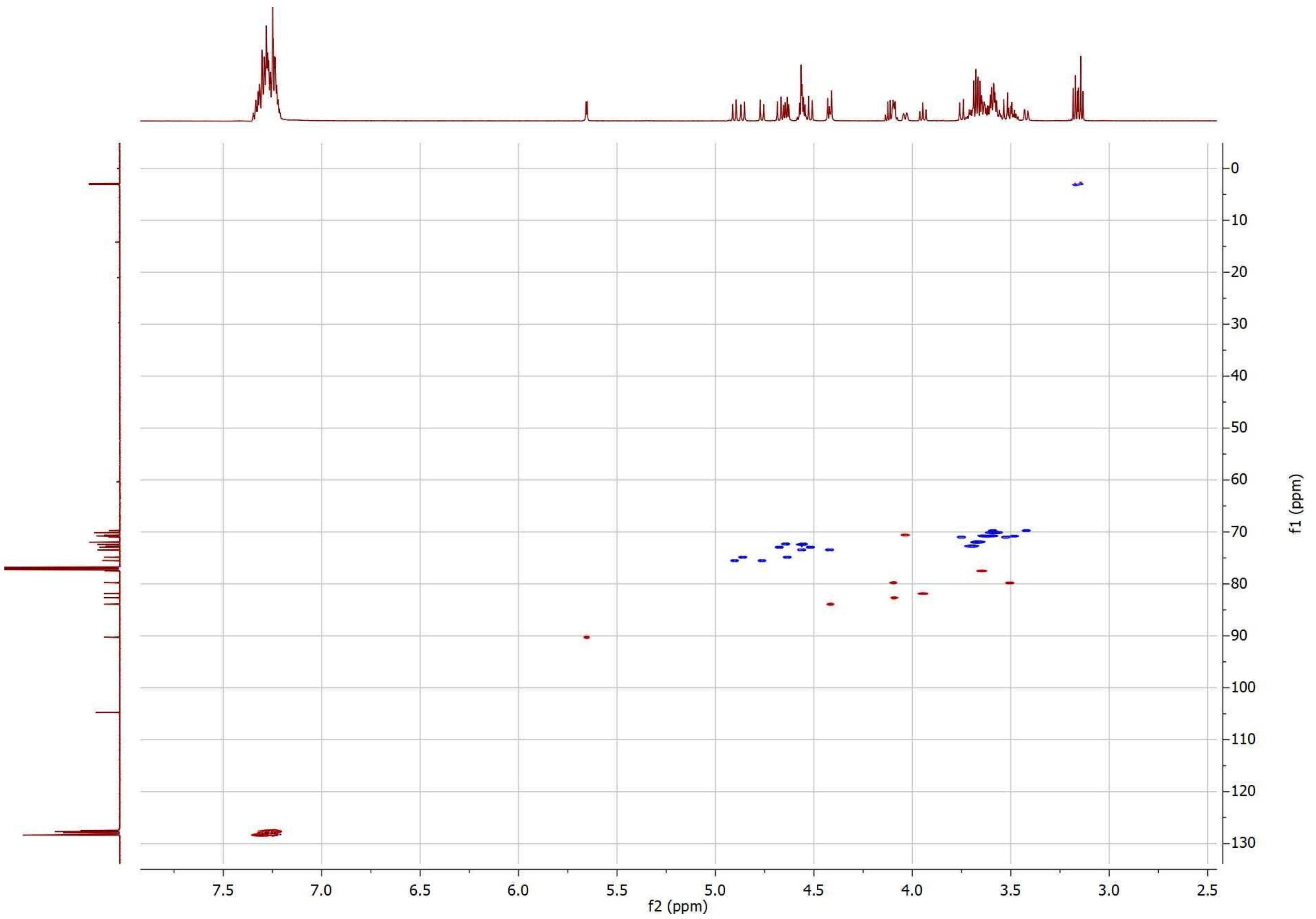

Figure S43. gHSQCAD spectrum of compound **7**.

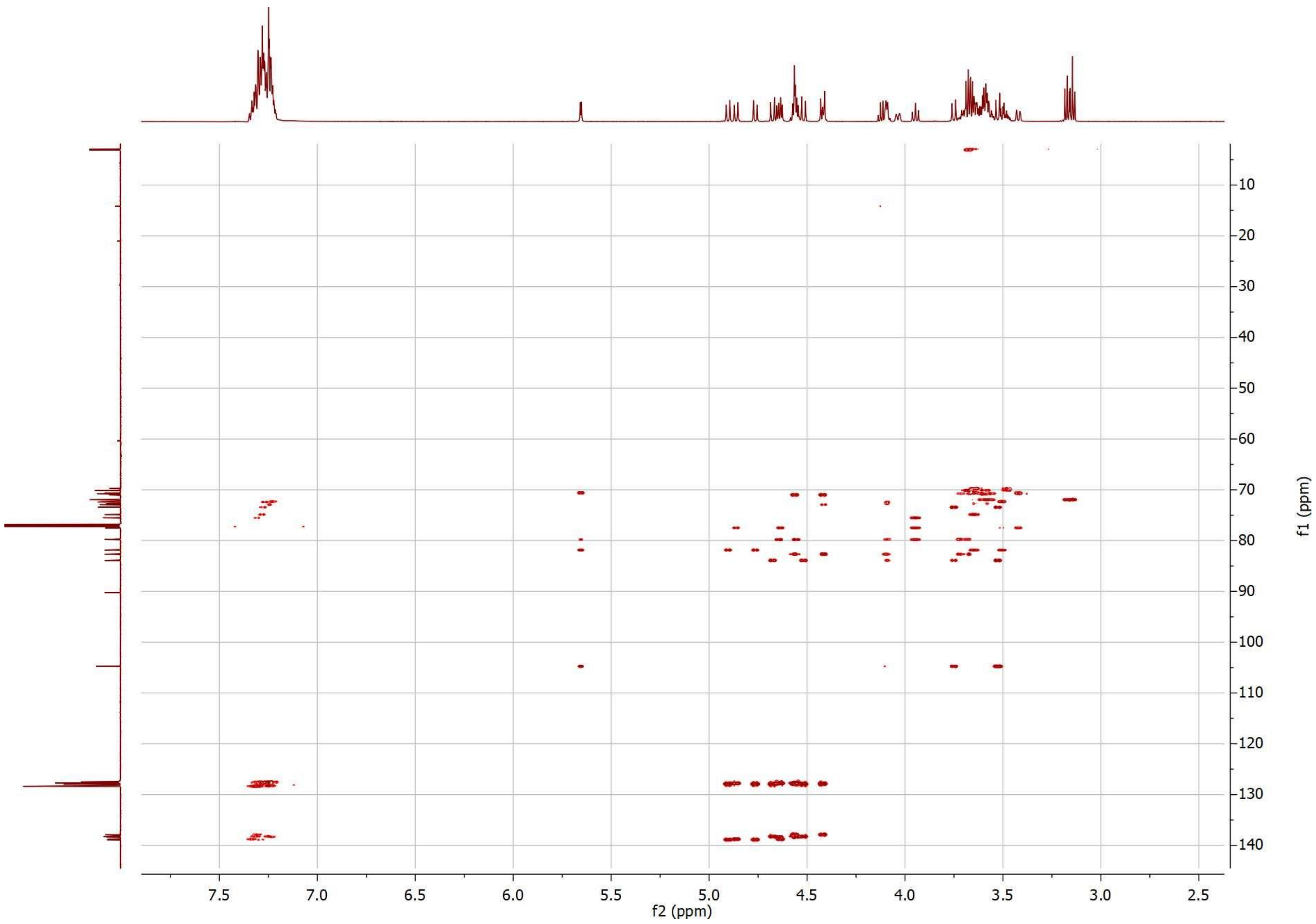

Figure S44. gHMBCAD spectrum of compound **7**.

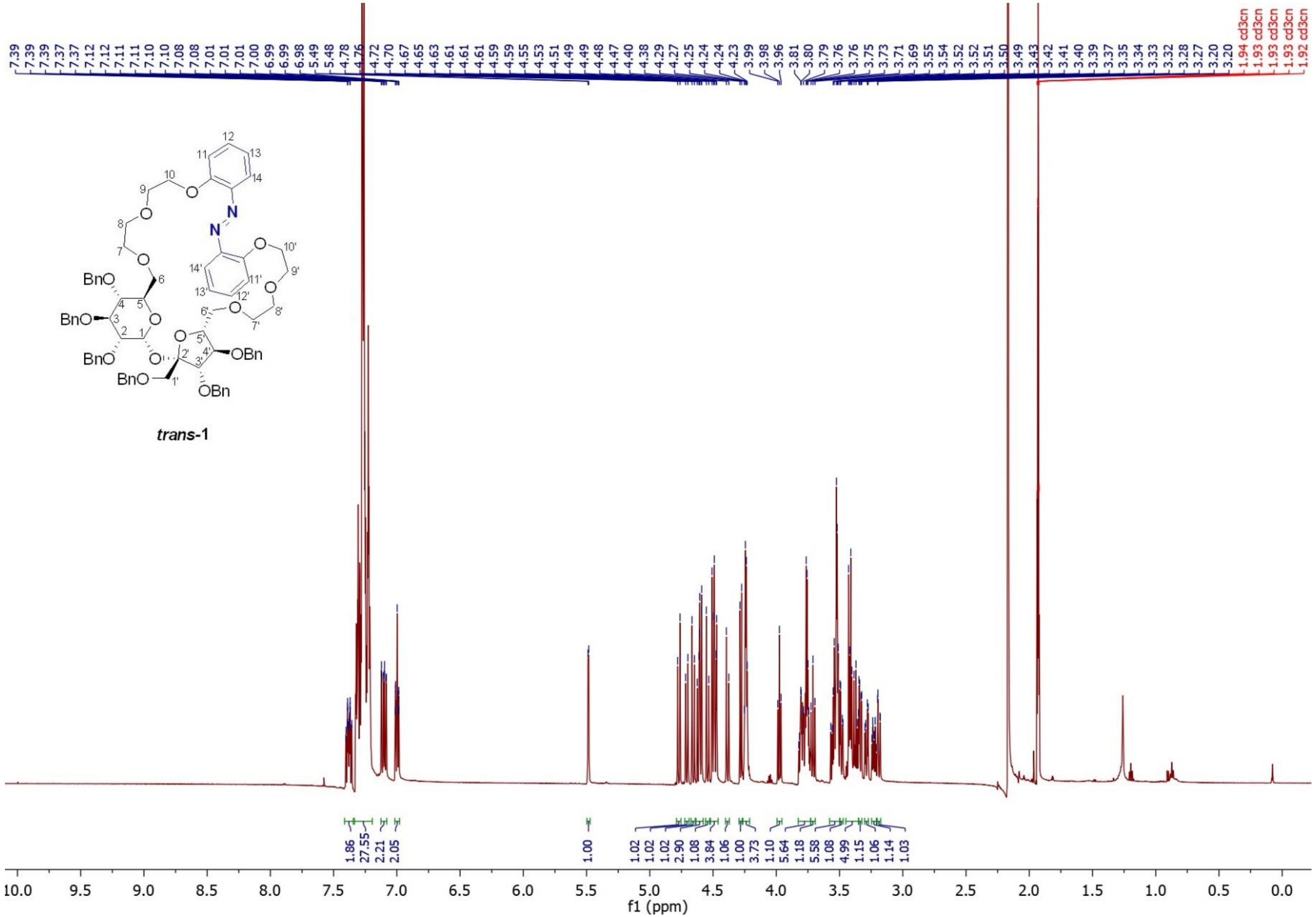

Figure S45. ^{1}H NMR (600 MHz, CD_3CN) spectrum of pure *trans*-**1**.

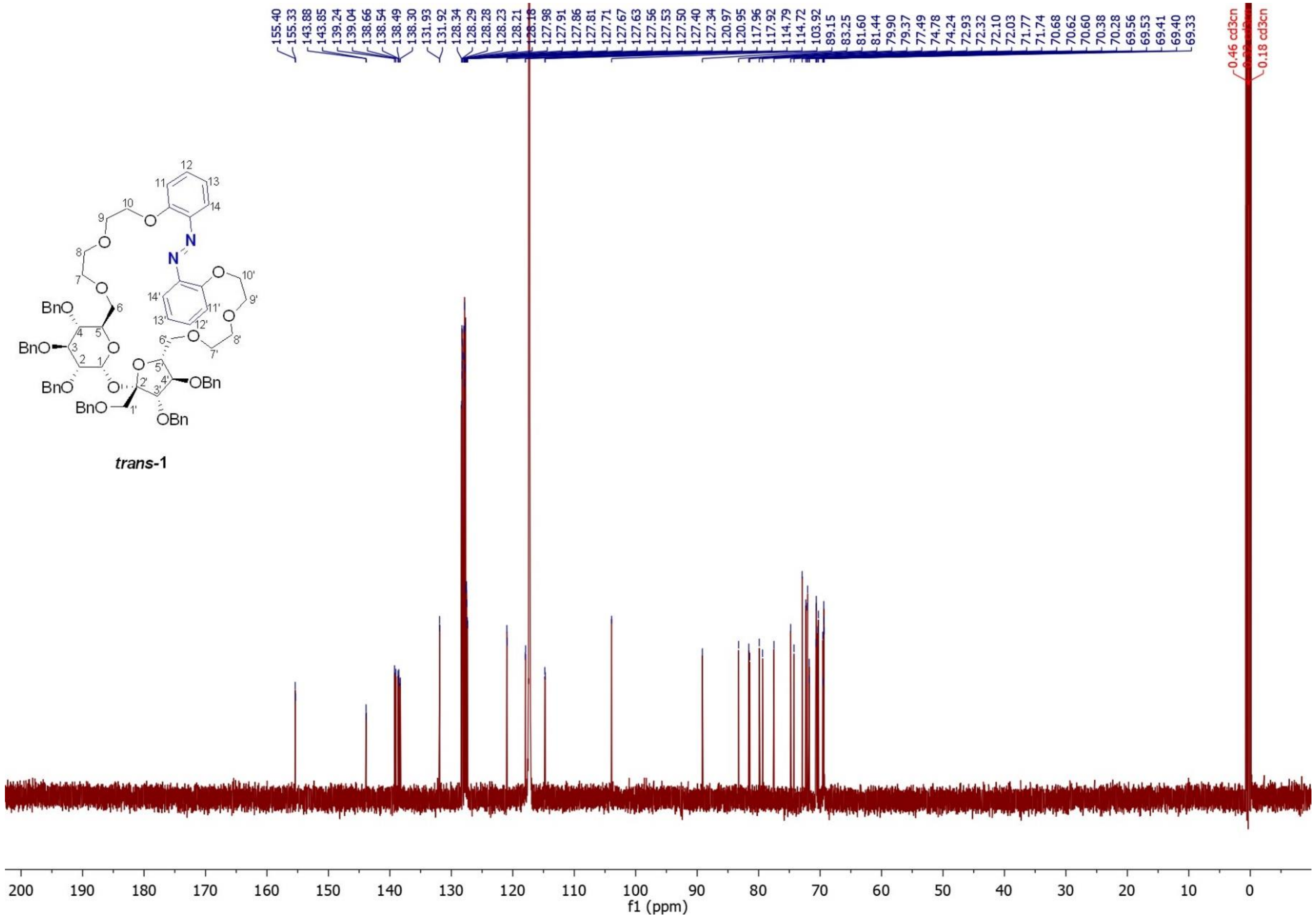

Figure S46. ^{13}C NMR (151 MHz, CD_3CN) spectrum of pure *trans*-**1**.

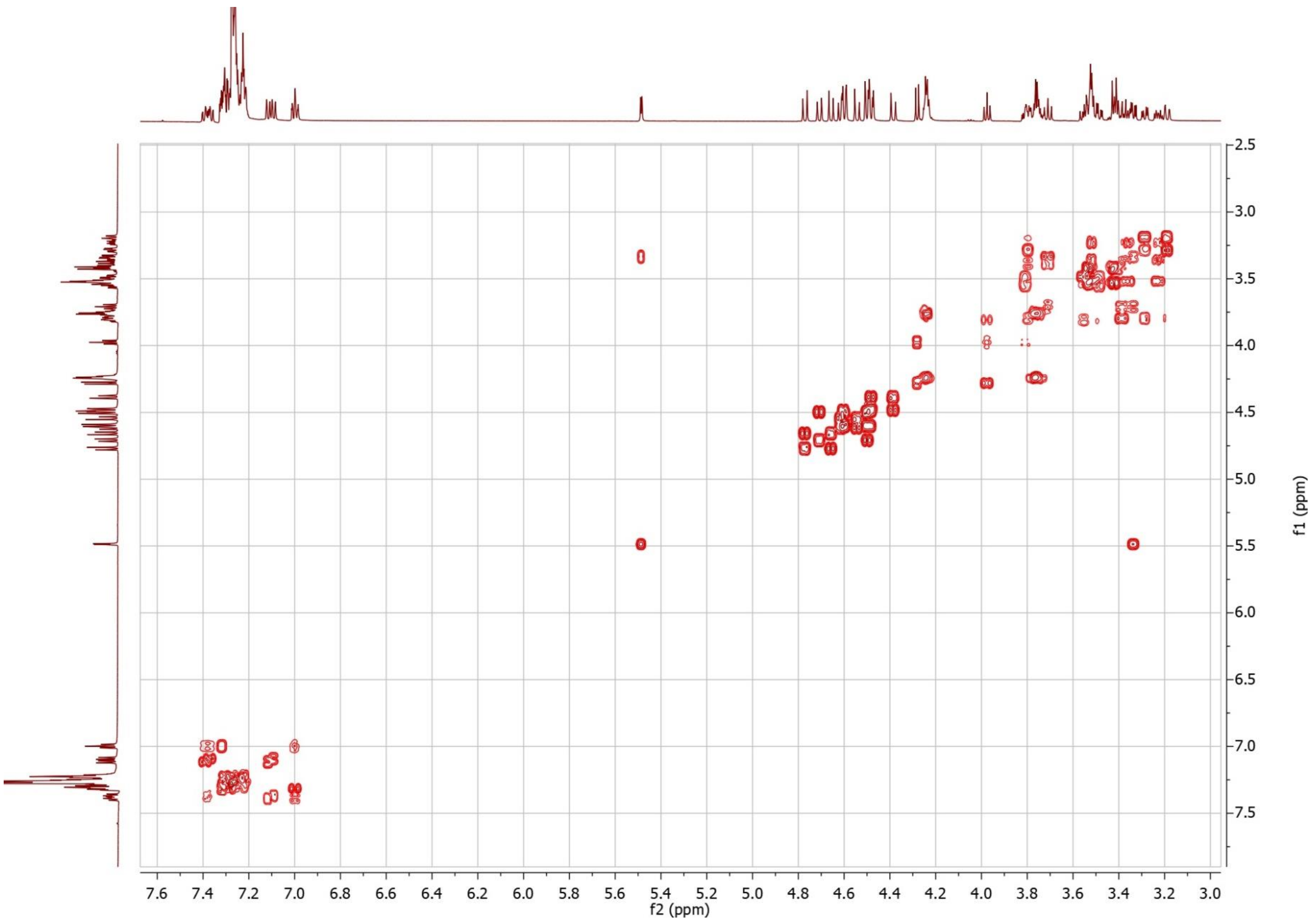

Figure S47. gCOSY spectrum of pure *trans*-**1**.

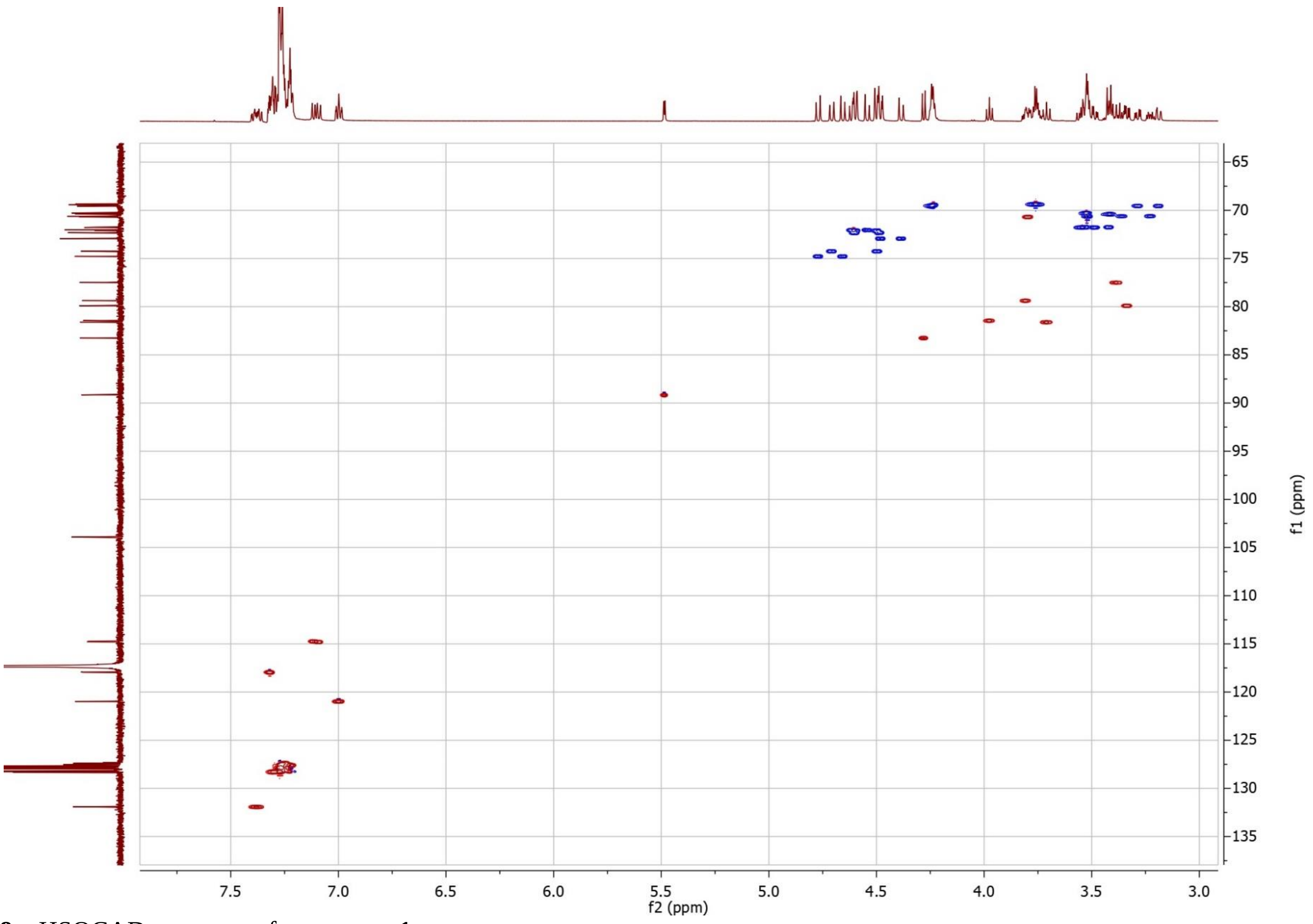

Figure S48. gHSQCAD spectrum of pure *trans*-**1**.

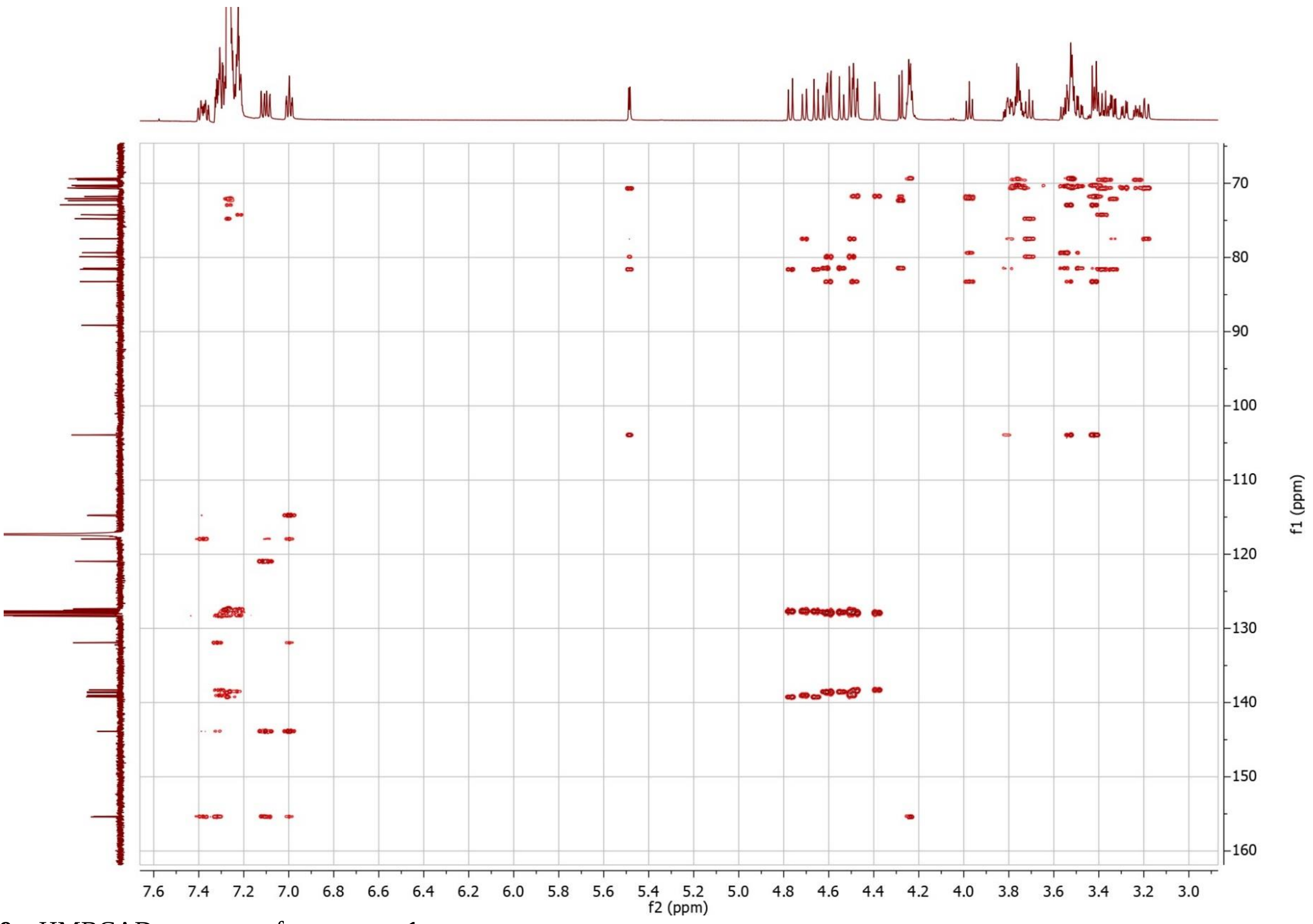

Figure S49. gHMBCAD spectrum of pure *trans*-**1**.

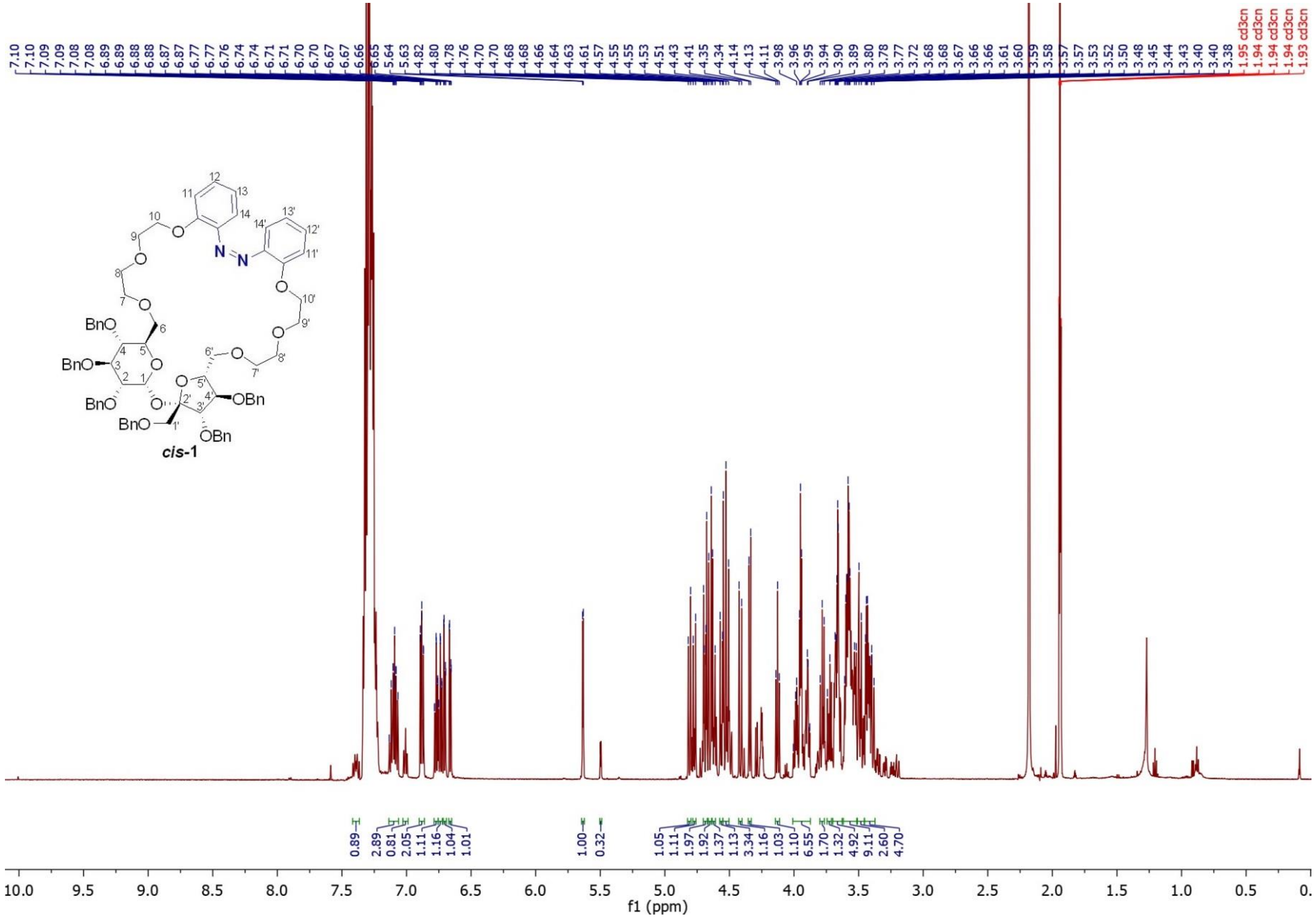

Figure S50. ^{1}H NMR (600 MHz, CD_3CN) spectrum of *cis*-**1** (as *cis*-enriched mixture containing ca. 24.2% of *trans*-isomer).

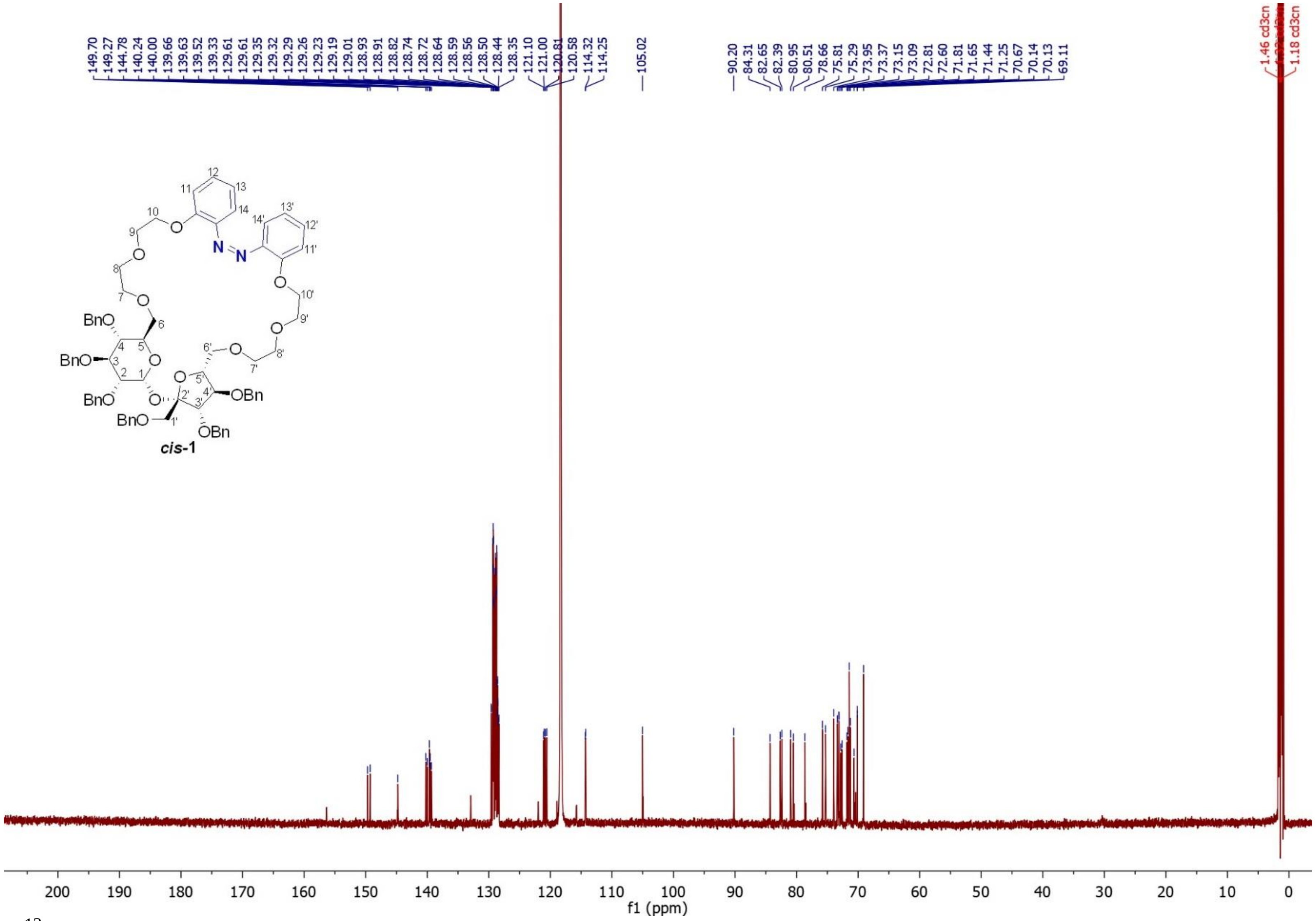

Figure S51. ^{13}C NMR (151 MHz, CD_3CN) spectrum of compound *cis*-**1** (as cis-enriched mixture containing ca. 24.2% of *trans*-isomer).

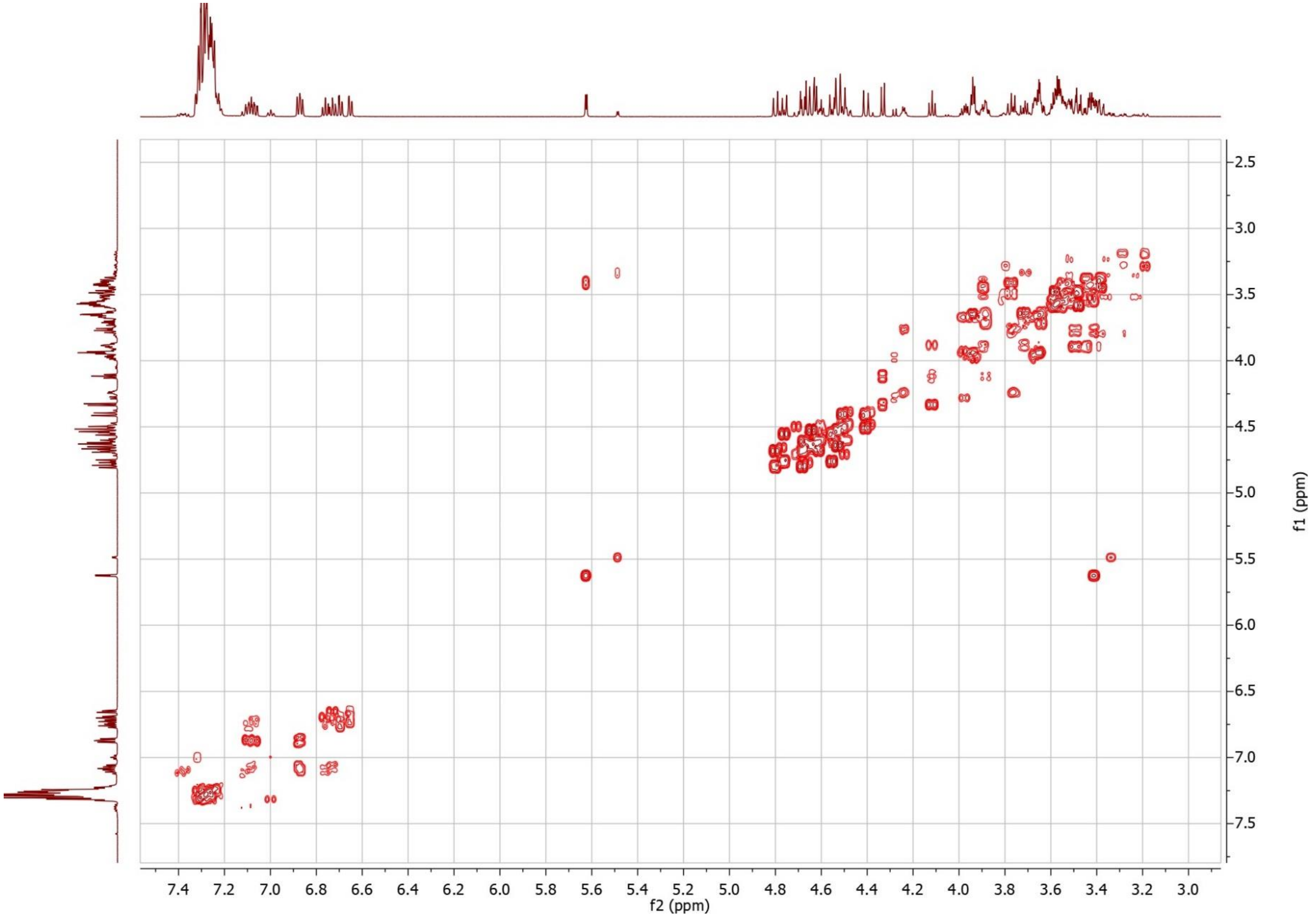

Figure S52. gCOSY spectrum of compound *cis*-**1** (as cis-enriched mixture containing ca. 24.2% of *trans*-isomer).

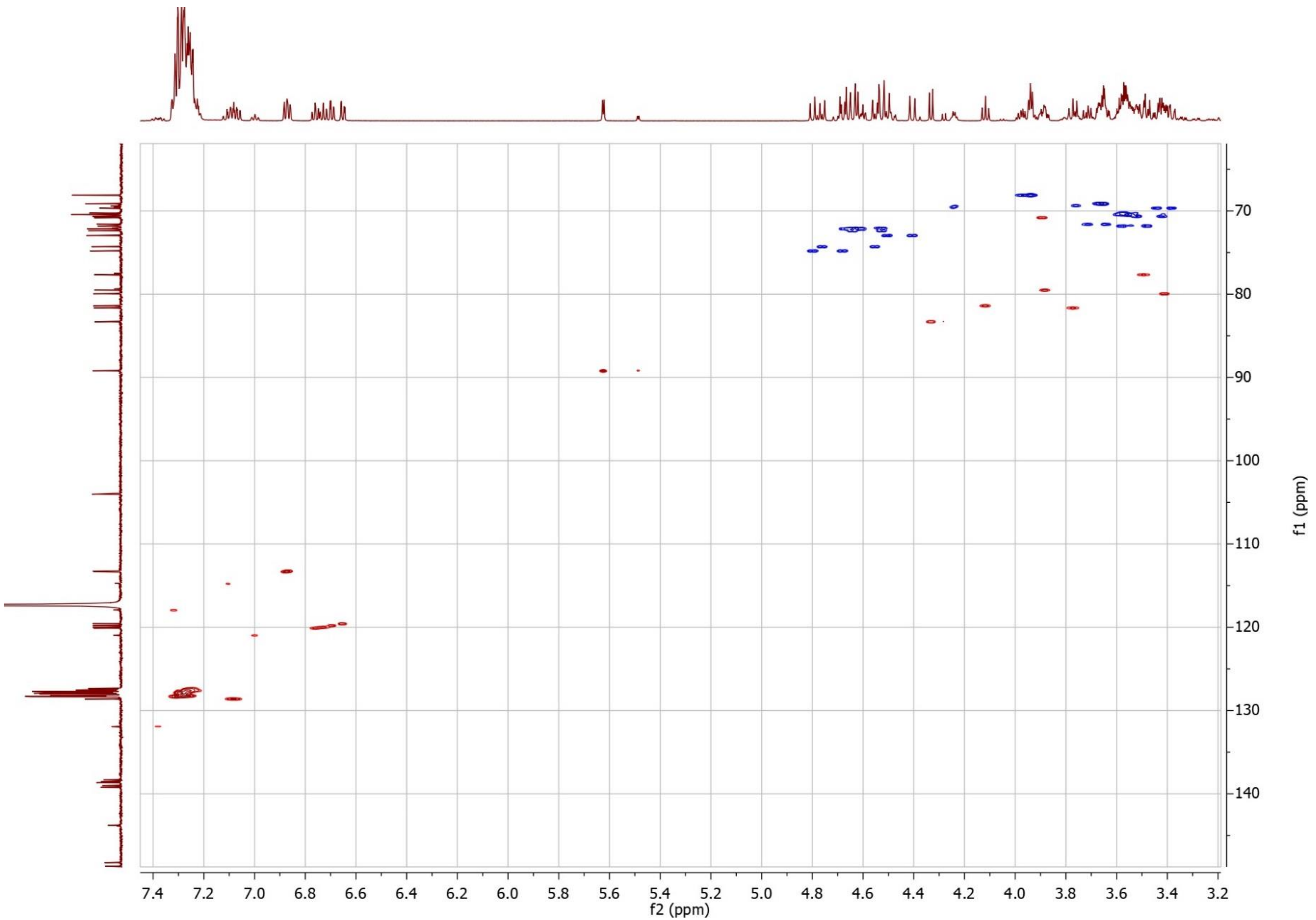

Figure S53. gHSQCAD spectrum of compound *cis*-**1** (as cis-enriched mixture containing ca. 24.2% of *trans*-isomer).

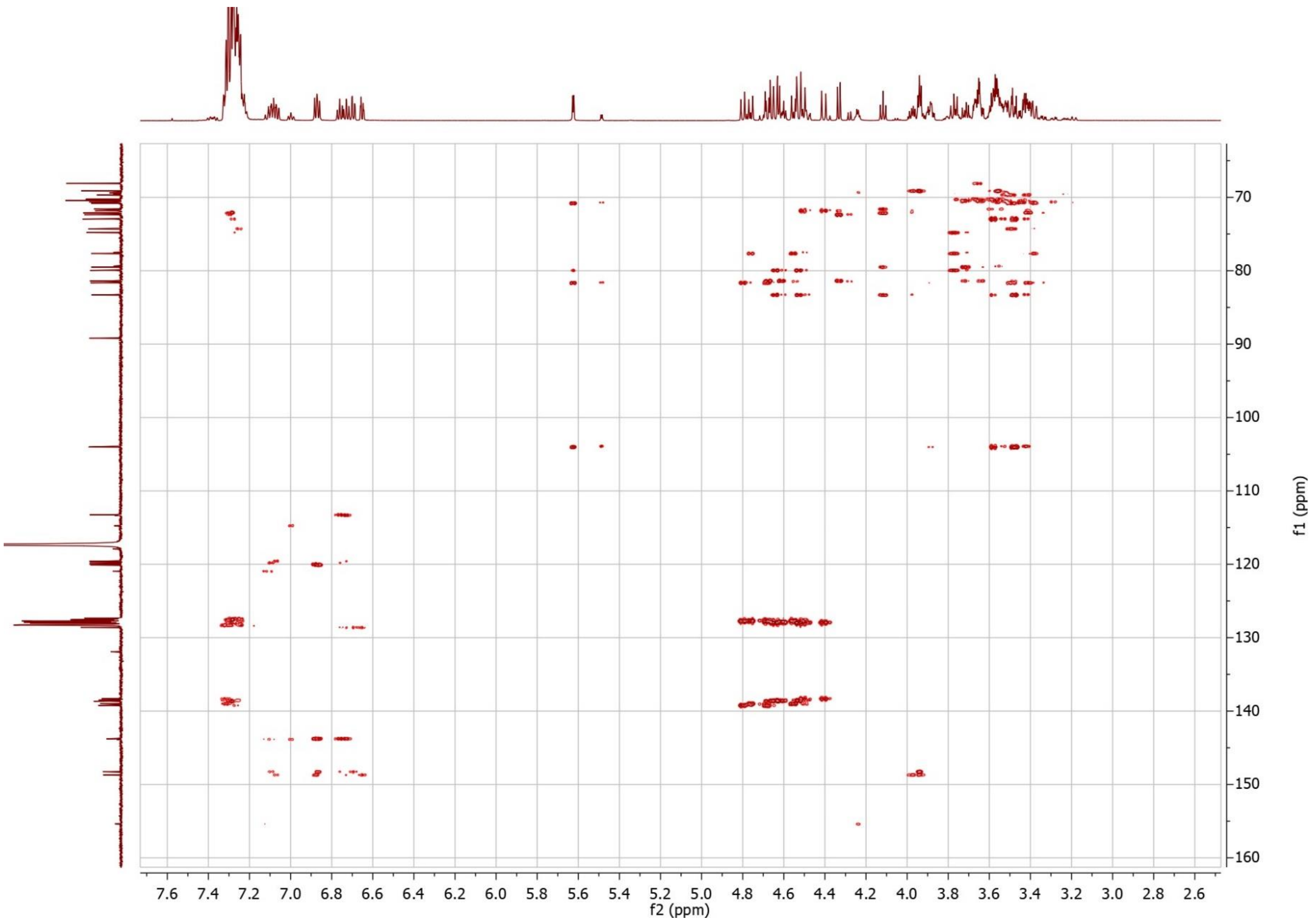

Figure S54. gHMBCAD spectrum of compound *cis*-**1** (as cis-enriched mixture containing ca. 24.2% of *trans*-isomer).

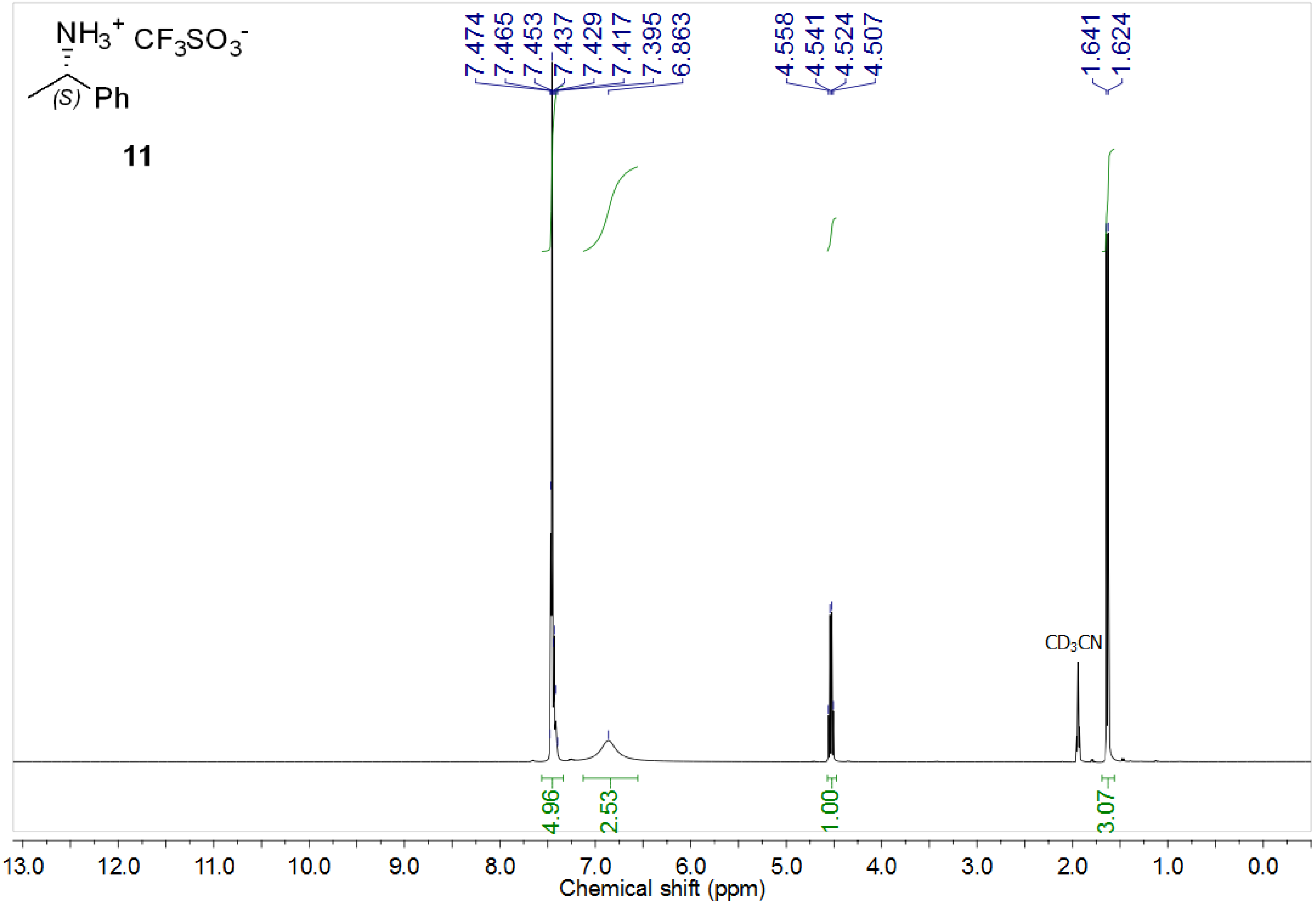

Figure S55. ^{1}H NMR (400 MHz, CD_3CN) spectrum of (*S*)-2-phenylethylammonium triflate (**11**).

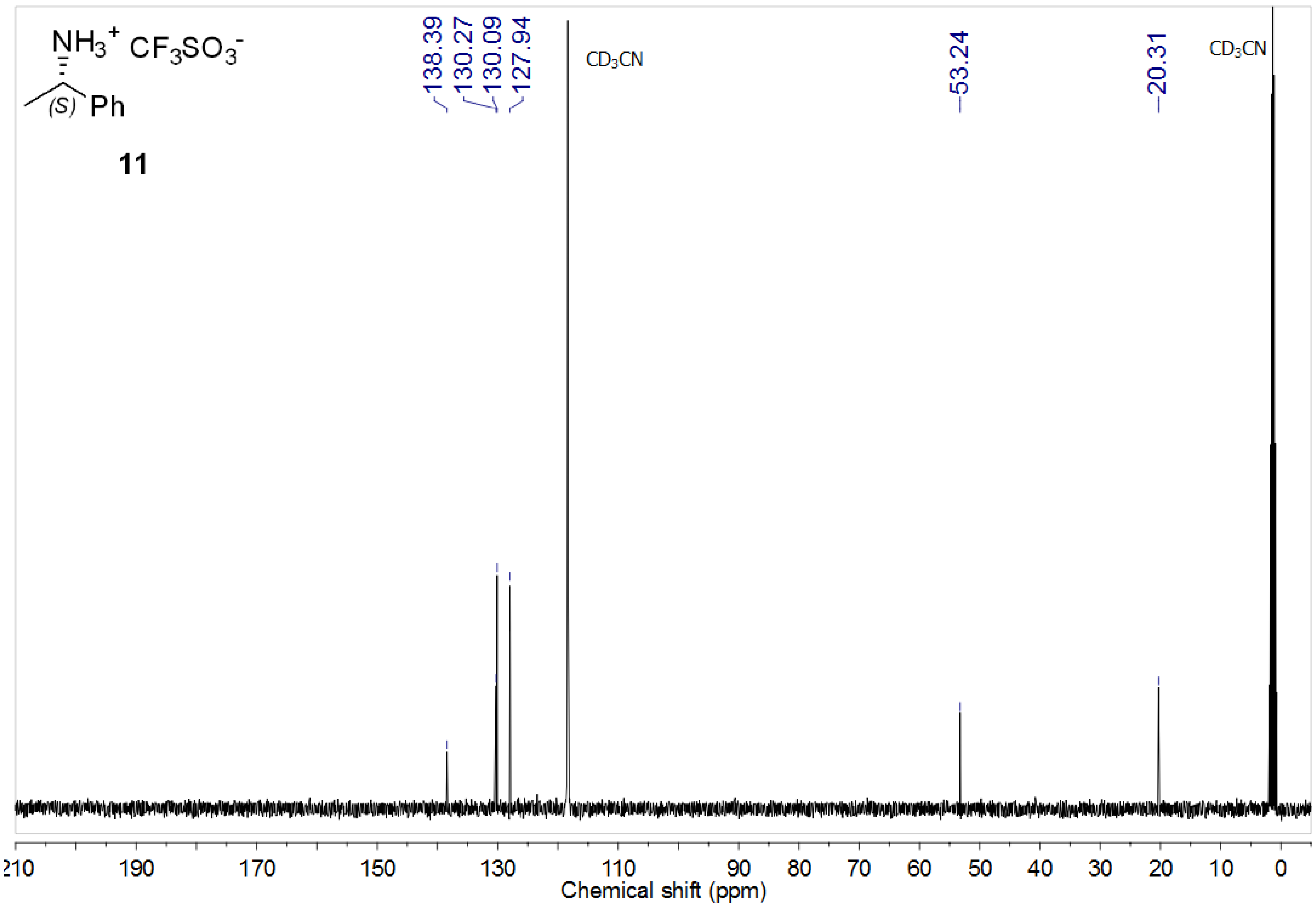

Figure S56. ^{13}C NMR (100 MHz, CD_3CN) spectrum of (*S*)-2-phenylethylammonium triflate (**11**).

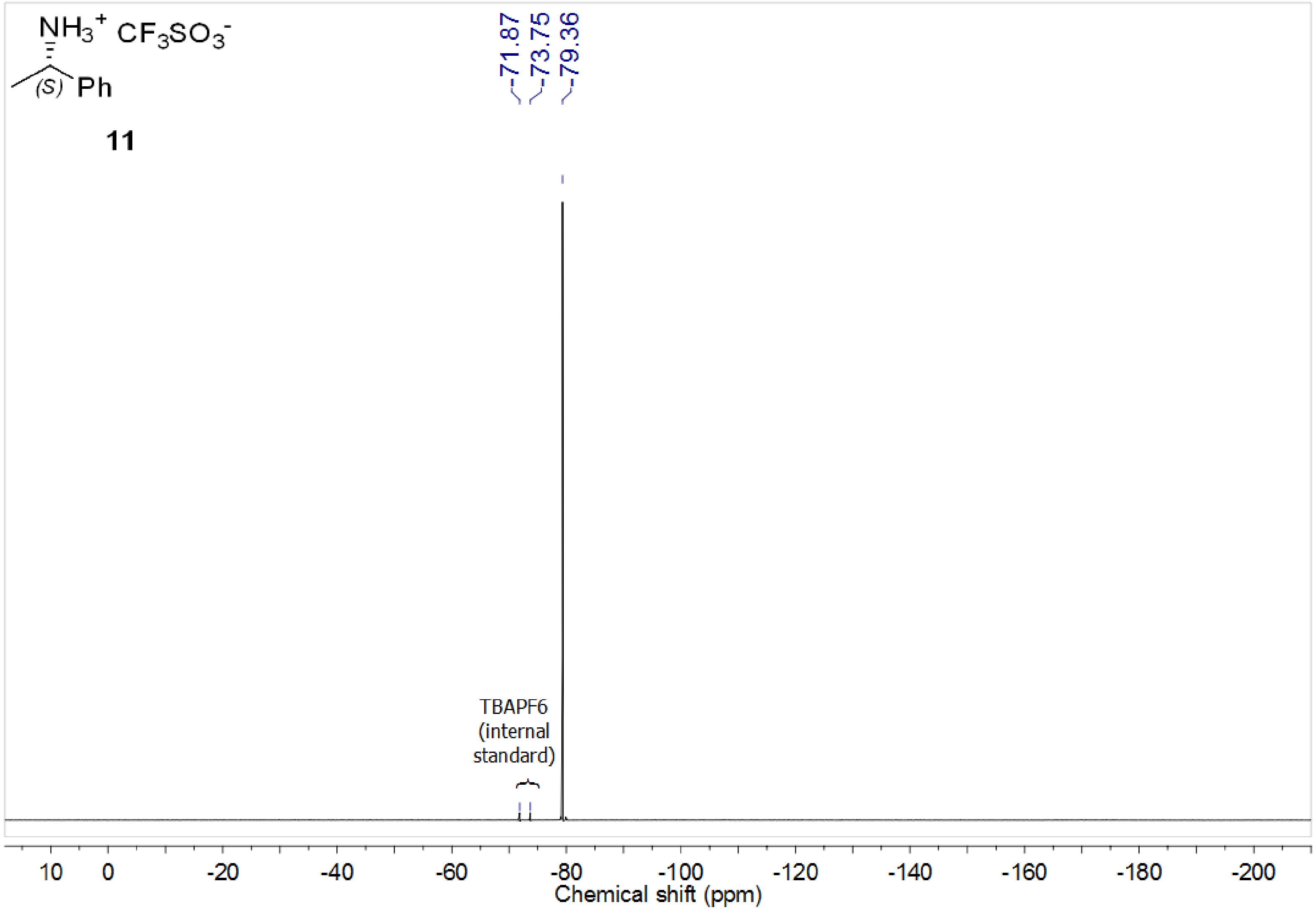

Figure S57. ^{19}F NMR (376 MHz, CD_3CN) spectrum of (*S*)-2-phenylethylammonium triflate (**11**).

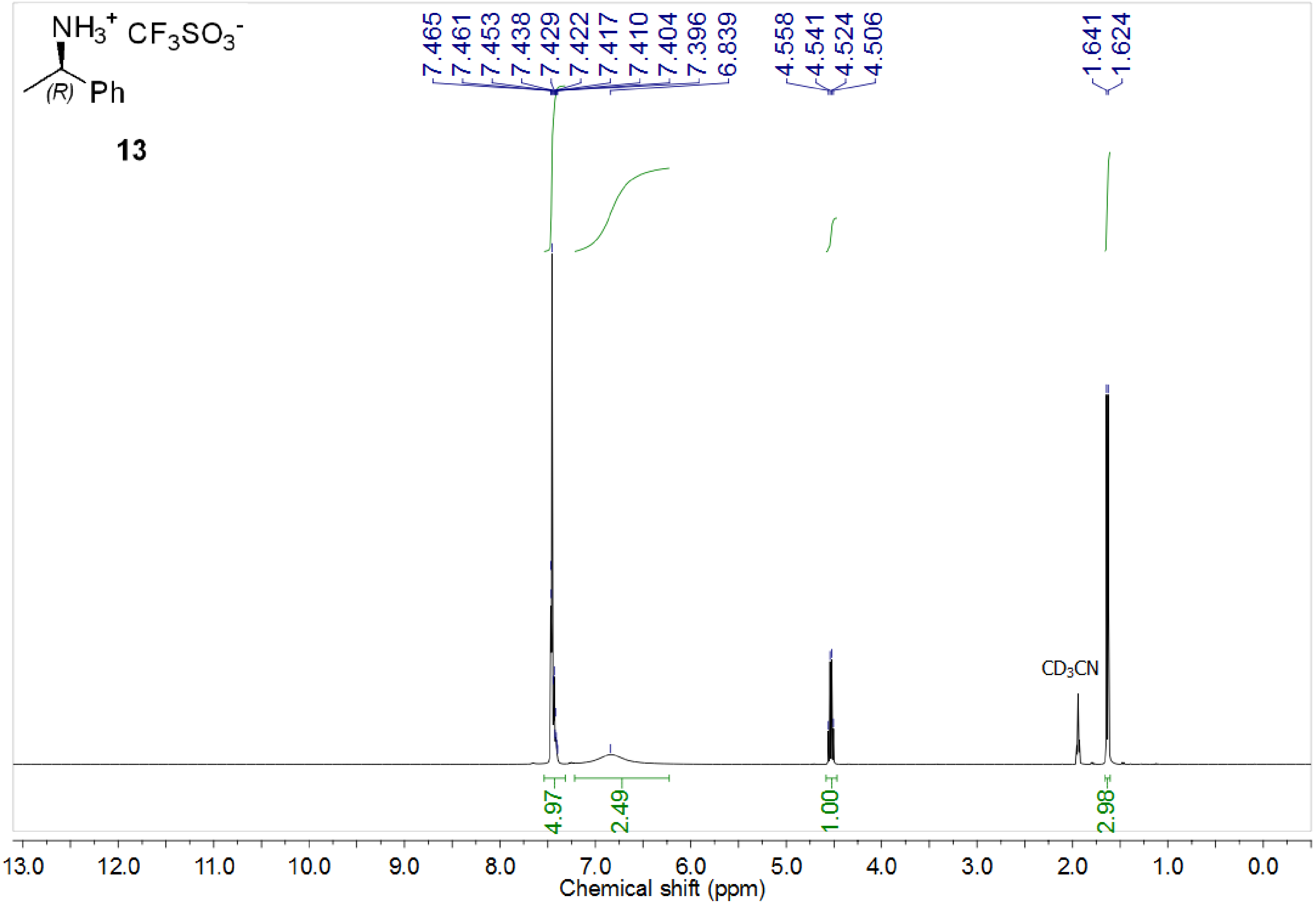

Figure S58. ^{1}H NMR (400 MHz, CD_3CN) spectrum of (*R*)-2-phenylethylammonium triflate (**13**).

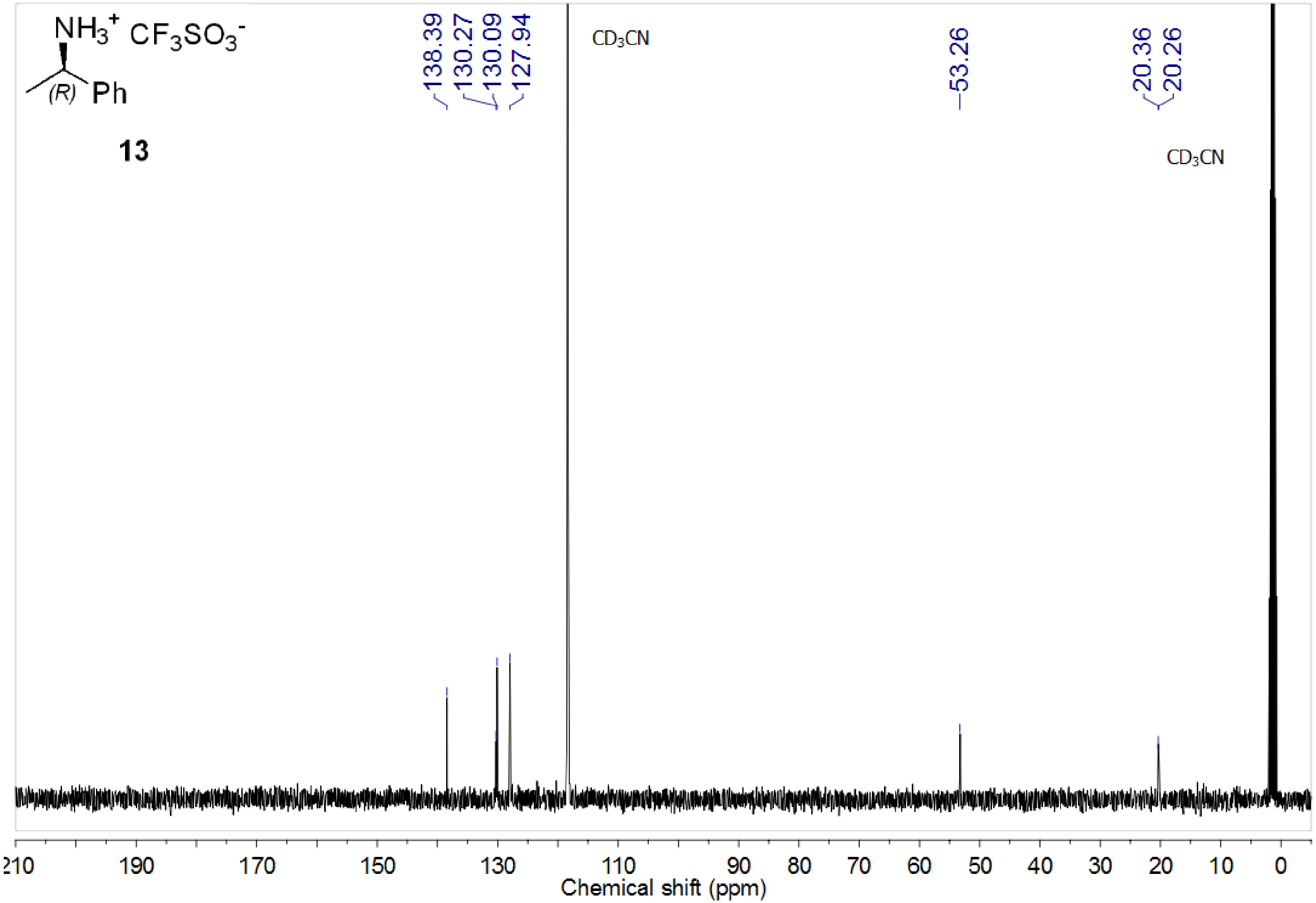

Figure S59. ^{13}C NMR (100 MHz, CD_3CN) spectrum of (*R*)-2-phenylethylammonium triflate (**11**).

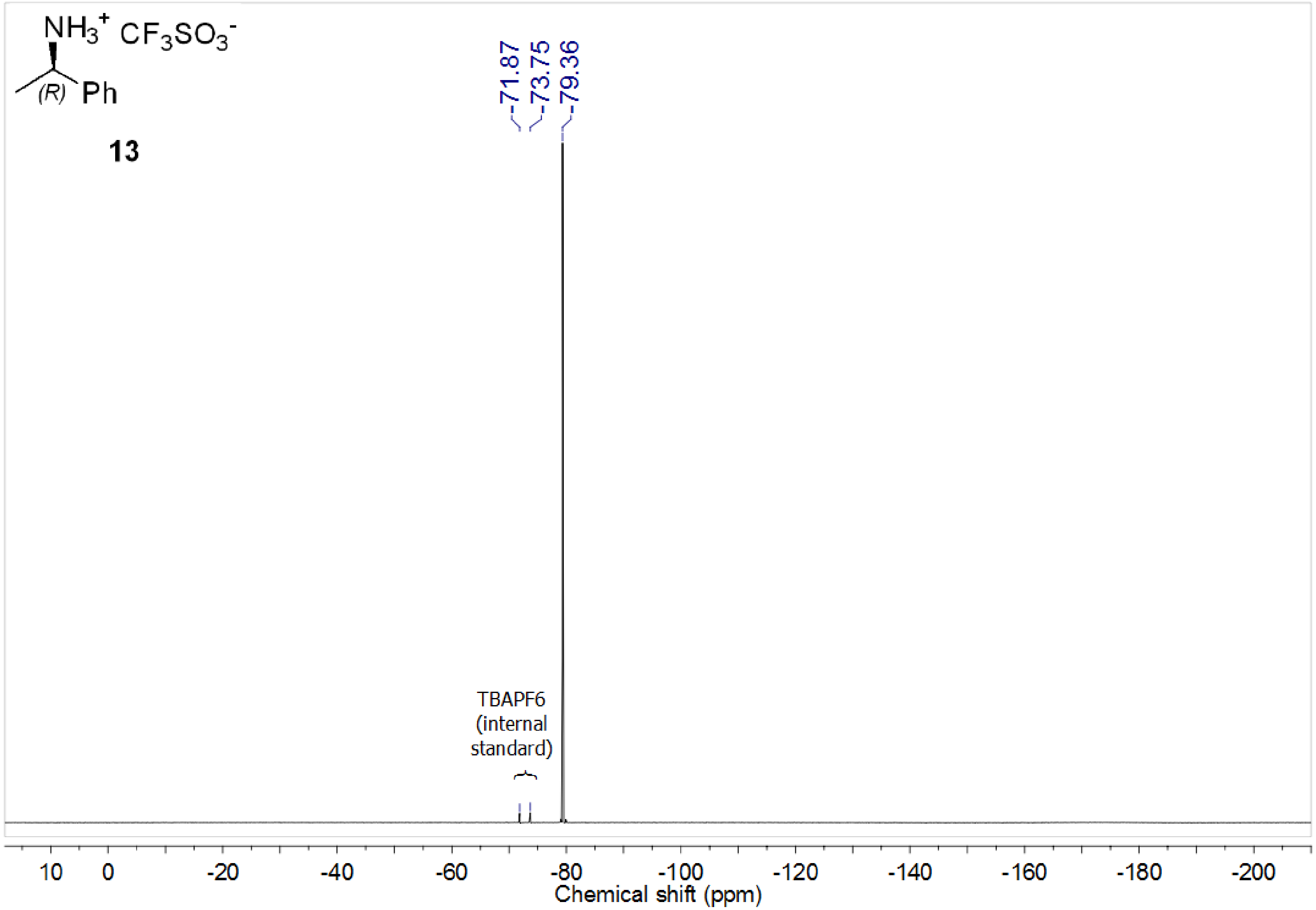

Figure S60. ^{19}F NMR (376 MHz, CD_3CN) spectrum of (*R*)-2-phenylethylammonium triflate (**13**).

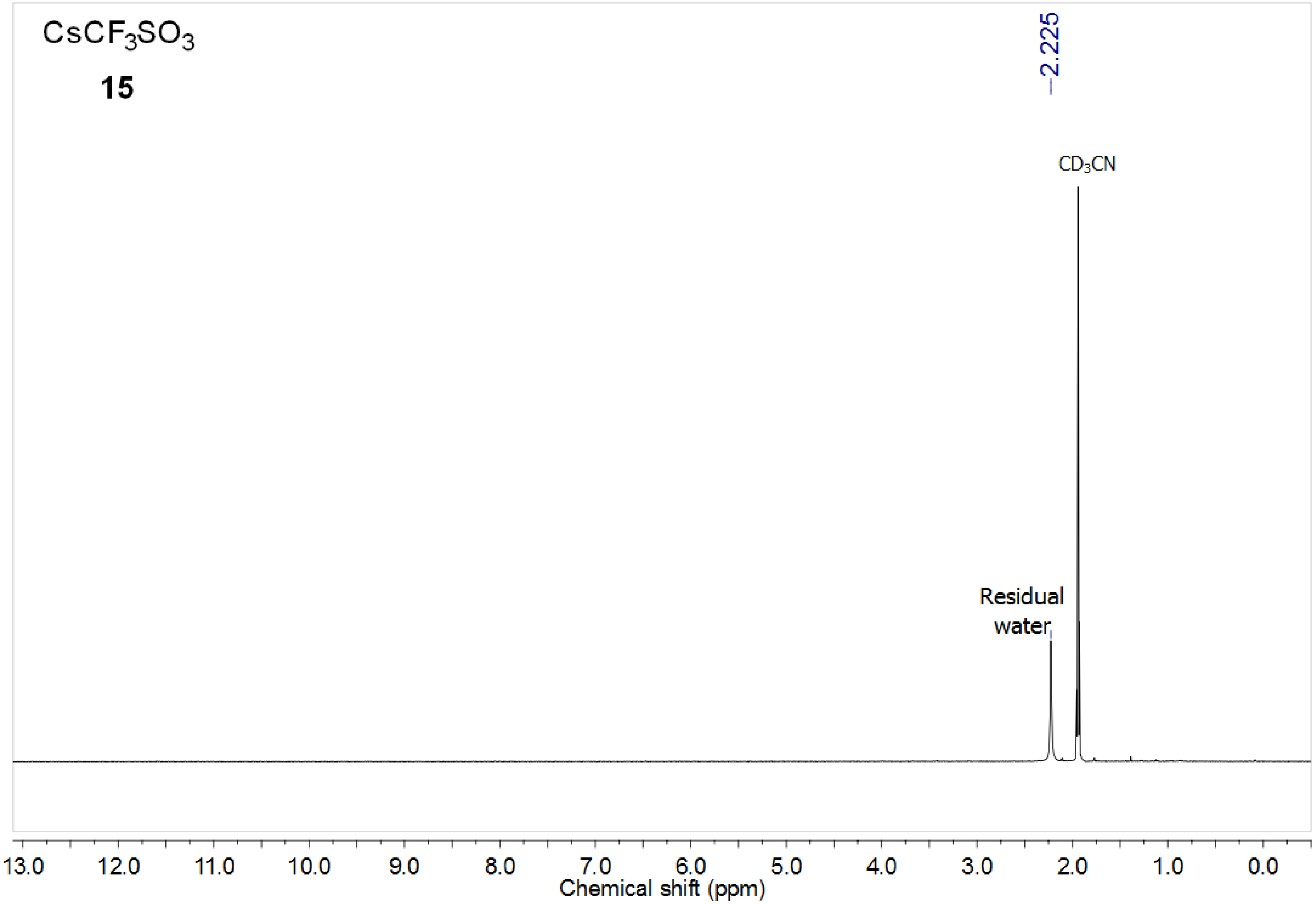

Figure S61. ^{1}H NMR (400 MHz, CD_3CN) spectrum of cesium triflate (**15**).

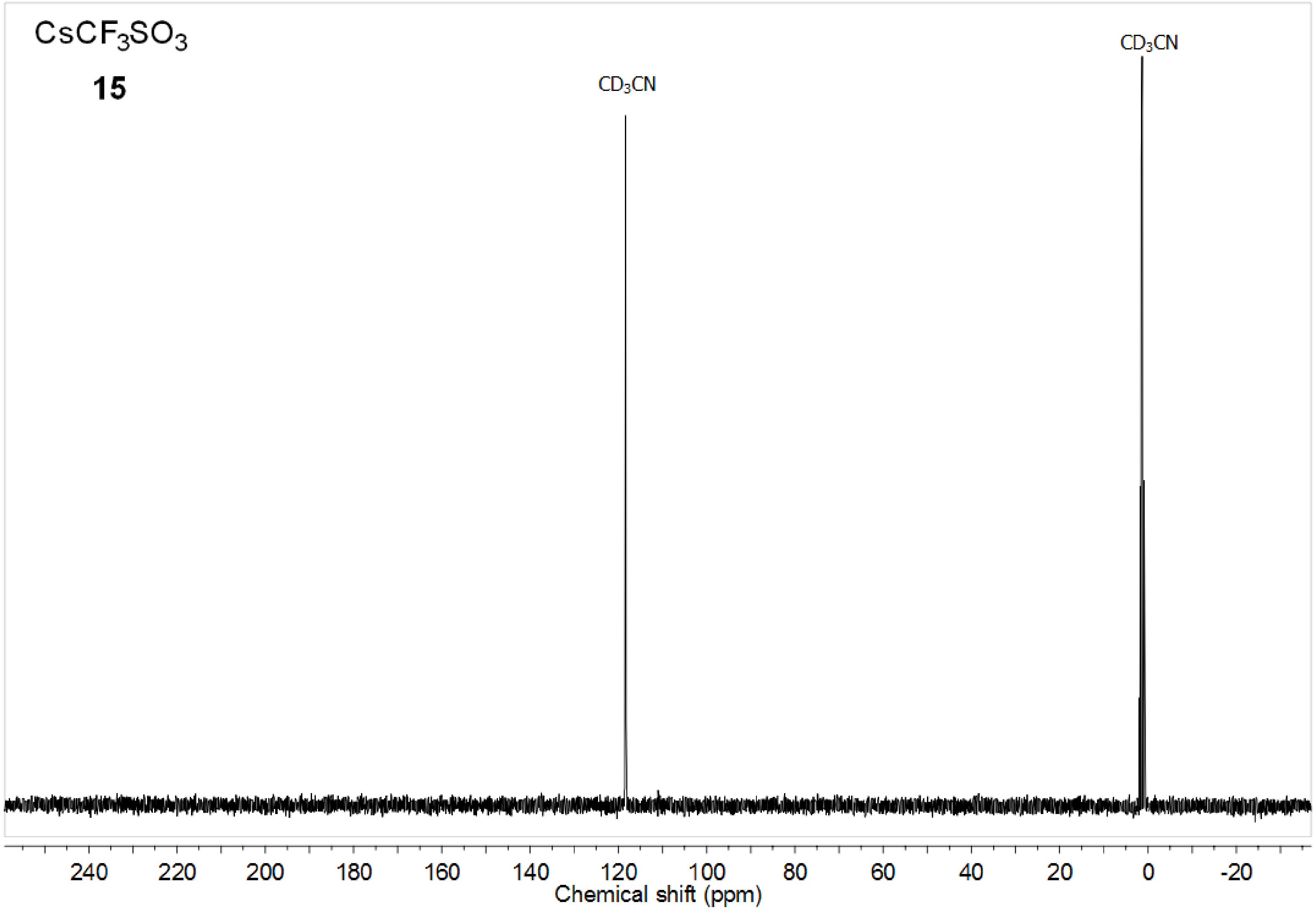

Figure S62. ^{13}C NMR (100 MHz, CD_3CN) spectrum of cesium triflate (**15**).

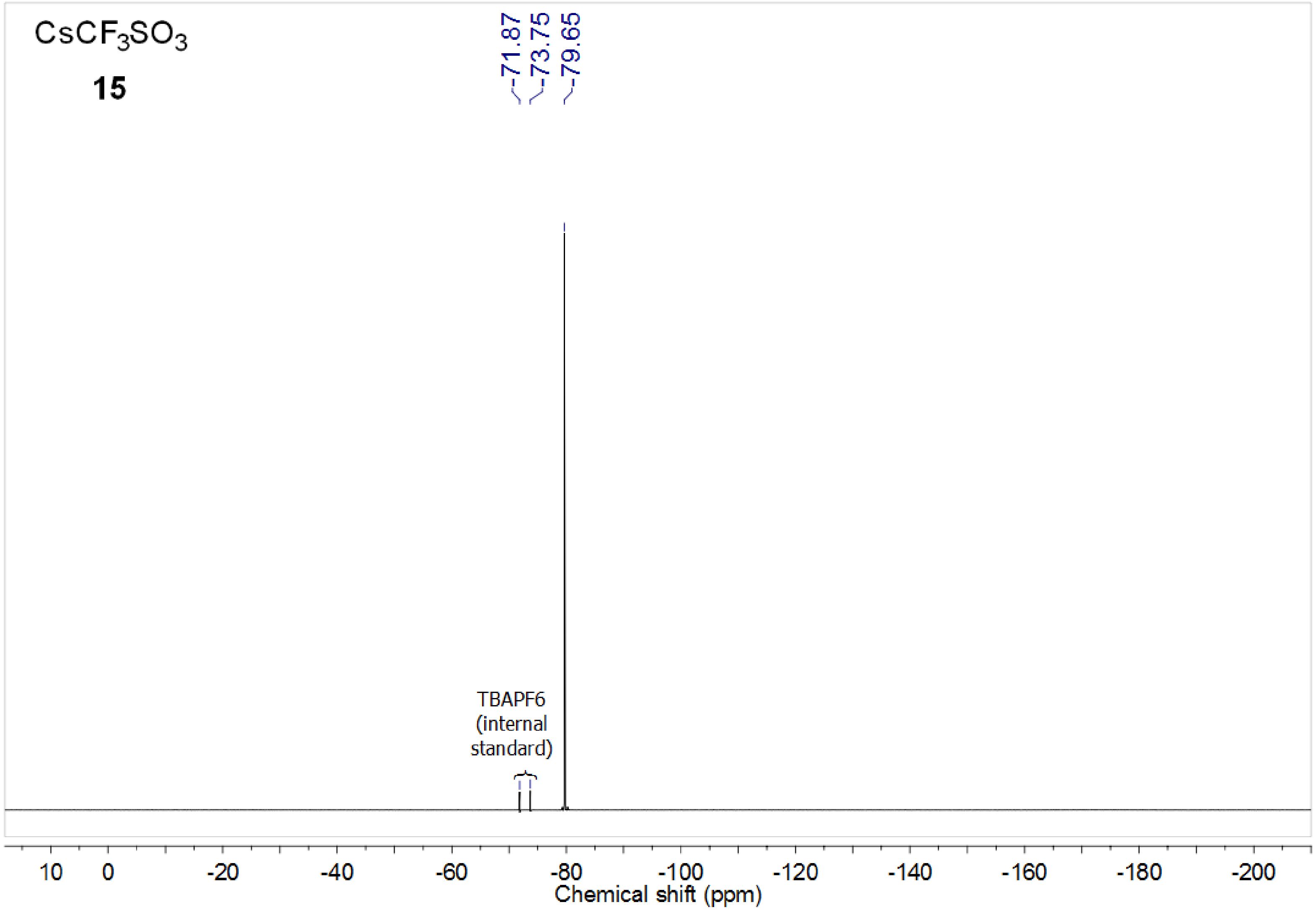

Figure S62. ^{19}F NMR (376 MHz, CD_3CN) spectrum of cesium triflate (**15**).

6. Cartesian coordinates of calculated structures

Table S3. Cartesian coordinates of the studied structures calculated at B3LYP-D3/6-31+G(d)/C-PCM:MeCN (ε=37.5) level of theory; the calculated total energies are given in atomic units (hartrees); 1 hartree = 2625.5 kJ·mol^{-1}; calculated energy for single potassium cation is -599.800432 hartrees; interaction energy for the complex (E_{int}) is defined as $E_{int} = E_{[K\subset\mathbf{1}]+} - (E_{\mathbf{1}} + E_{K+})$ and is given in kJ·mol^{-1}.

trans-**1**<br>*E* = -4106.01615 hartrees	*cis*-**1**<br>*E* = -4106.00079 hartrees<br>$\Delta E_{cis\text{-}trans}$ = 40.3 kJ·mol^{-1}	[K⊂*trans*-**1**]$^+$<br>*E* = -4705.927903 hartrees<br>ΔE_{int} = -292.3 kJ·mol^{-1}	[K⊂*cis*-**1**]$^+$<br>*E* = -4705.913807 hartrees<br>ΔE_{int} = -295.6 kJ·mol^{-1}
C -3.145017 -1.128495 0.140630	C -1.345020 0.510986 -1.234499	C -0.367174 2.782328 0.667116	C -2.795330 -0.432170 -0.304498
C -3.101515 0.378056 -0.205213	C 0.097149 0.059440 -1.528345	C 1.063019 2.503766 0.163989	C -2.396494 1.056431 -0.317196
C -1.664244 0.887377 -0.440474	C 0.928195 -0.192392 -0.256908	C 1.373608 1.006650 -0.011820	C -0.872023 1.309895 -0.329630
C -0.979916 -0.046196 -1.441082	C 0.114822 -1.043067 0.724633	C 0.226192 0.326659 -0.753159	C -0.150970 0.339256 -1.269552
O -0.956610 -1.369353 -0.935407	O -1.134430 -0.440268 1.005622	O -0.994618 0.531941 -0.051833	O -0.559911 -1.006085 -1.045405
C -2.245589 -1.966190 -0.783846	C -1.993284 -0.327122 -0.128950	C -1.419190 1.906875 -0.017699	C -1.954377 -1.262329 -1.271828
C 1.219100 -0.203258 -2.520016	C 0.441071 -1.912502 3.008358	C -0.287891 -1.972298 -1.479590	C 2.191877 -0.144555 -1.858351
C 2.544969 0.580471 -2.386430	C 1.566645 -1.696764 4.037268	C 0.606305 -3.219375 -1.568514	C 3.359989 0.866461 -1.965154
C 3.271641 -0.128287 -1.222308	C 1.231016 -0.285705 4.537005	C 0.537143 -3.744103 -0.127456	C 4.337879 0.481044 -0.846599
C 2.495257 -1.446574 -1.033684	C -0.312113 -0.227088 4.439446	C -0.872848 -3.318348 0.348524	C 3.819695 -0.872874 -0.316806
O 1.582157 -1.514635 -2.149326	O -0.693031 -1.402327 3.678222	O -1.371964 -2.412176 -0.674391	O 2.756359 -1.280835 -1.215549
C -2.875543 -2.313193 -2.149902	C -2.503624 -1.689963 -0.621595	C -1.885976 2.395775 -1.392396	C -2.337204 -1.109266 -2.753648
O -1.913157 -2.787859 -3.084859	O -2.919643 -2.554029 0.437216	O -2.695252 1.348014 -1.920516	O -1.409560 -1.776809 -3.598764
C 0.652712 -0.305803 -3.940048	C 0.091081 -3.352890 2.627701	C -0.916714 -1.470750 -2.780319	C 1.691106 -0.588145 -3.237265
C 1.724879 -1.526351 0.280491	C -0.792237 1.039011 3.747545	C -0.869255 -2.625692 1.699852	C 3.345644 -0.724223 1.119615
O 2.671357 -1.651227 1.335625	O -2.207350 1.021200 3.630635	O -2.218240 -2.336843 2.066132	O 2.942541 -1.985169 1.648757
C -1.209303 -3.950778 -2.656247	C -3.835563 -1.980106 1.378240	C -3.561586 1.728600 -2.986279	C -1.503064 -3.203356 -3.611820
C -0.150753 -4.280547 -3.706472	C -4.820981 -3.038575 1.892177	C -3.993645 0.475775 -3.720754	C -0.135947 -3.813235 -3.837599
O 0.786304 -5.234068 -3.204125	O -6.137983 -2.898220 1.351601	O -4.649263 -0.408255 -2.815620	O 0.708187 -3.584694 -2.707963
C 2.129770 -1.337169 2.617778	C -2.678128 2.057233 2.779650	C -2.350397 -1.787188 3.381279	C 2.636393 -1.911407 3.049708
C 0.845426 -2.115051 2.929942	C -4.160871 1.802297 2.513072	C -2.218955 -0.274757 3.423406	C 1.233073 -1.377061 3.296033
O 0.859247 -2.435927 4.323478	O -4.744743 2.846488 1.736314	O -3.351734 0.294306 2.759451	O 0.267098 -2.294496 2.782283
C 1.821260 -4.634648 -2.430435	C -6.435978 -3.610096 0.160281	C -4.945910 -1.669547 -3.409372	C 1.874874 -4.405974 -2.695562

C 2.748367 -5.740498 -1.950045	C -5.650725 -3.166699 -1.070245	C -5.794023 -2.480816 -2.456802	C 1.615141 -5.753789 -2.044336
O 3.884453 -5.155672 -1.287107	O -5.646886 -1.729307 -1.217449	O -4.993852 -2.791112 -1.292955	O 1.335016 -5.534203 -0.637105
C -0.334263 -3.041557 4.805756	C -4.410299 2.821729 0.348178	C -3.588977 1.654511 3.119232	C -0.257347 -3.228701 3.724284
O -1.430979 -3.937276 2.877278	O -4.750020 1.336321 -1.603674	O -4.505277 2.227946 0.983822	O -1.080275 -4.553825 1.825832
C 3.976195 -4.859074 0.036422	C -3.415637 2.153884 -4.887714	C -5.625460 -3.022206 -0.091382	C -4.456477 -4.702973 0.462668
C -1.609706 -4.850152 1.875051	C -6.565330 0.036643 -4.295399	C -5.555968 2.458210 0.131200	C -1.600567 -7.444176 0.522205
C 2.965884 -4.910054 1.033419	C -3.886636 1.423980 -3.791809	C -6.407840 -2.036689 0.550303	C -3.059699 -4.751433 0.557476
C 3.306763 -4.572693 2.364513	C -4.468620 2.098233 -2.690070	C -6.841559 -2.242012 1.864185	C -2.440062 -4.633124 1.824168
C 4.583205 -4.175340 2.711639	C -4.678461 3.478849 -2.783941	C -6.516295 -3.417991 2.540014	C -3.240079 -4.575852 2.968114
C 5.566658 -4.083417 1.712287	C -4.263848 4.182390 -3.916825	C -5.736483 -4.389447 1.906611	C -4.631627 -4.609377 2.856915
C 5.264974 -4.423048 0.403096	C -3.616635 3.529400 -4.967855	C -5.289093 -4.187096 0.599771	C -5.249091 -4.662031 1.606197
C -2.929357 -5.117661 1.496903	C -7.951871 0.105587 -4.162809	C -5.665530 3.708045 -0.477753	C -0.974400 -8.443806 1.264900
C -3.205072 -5.951869 0.415552	C -8.576984 -0.450011 -3.042819	C -6.659301 3.931693 -1.430939	C 0.418874 -8.465418 1.383788
C -2.155140 -6.532455 -0.306946	C -7.817955 -1.084350 -2.056148	C -7.551097 2.908558 -1.782957	C 1.194723 -7.504482 0.729368
C -0.839464 -6.258060 0.045788	C -6.433367 -1.177295 -2.195025	C -7.443451 1.657854 -1.185942	C 0.575370 -6.520892 -0.035059
C -0.541876 -5.410316 1.129249	C -5.799956 -0.605428 -3.313171	C -6.445934 1.421400 -0.226583	C -0.830205 -6.463611 -0.115625
C -0.747926 -4.320880 4.088722	C -5.372203 1.914069 -0.441974	C -4.818662 2.145656 2.392547	C -0.349505 -4.594475 3.065701
N 1.655972 -5.234072 0.640526	N -3.580610 0.023140 -3.796996	N -6.718805 -0.829500 -0.147411	N -2.377705 -4.768348 -0.698187
N 0.770017 -5.088176 1.531492	N -4.408879 -0.867842 -3.495848	N -6.213497 0.180369 0.405585	N -1.373100 -5.468804 -0.974772
O 0.393168 0.982252 -4.468297	O 1.189054 -3.980707 1.990285	O 0.071762 -1.162822 -3.744268	O 1.574037 0.562632 -4.068047
C -1.388150 2.621028 -4.210159	C 2.079787 -4.120872 -0.260441	C 1.470160 0.780730 -4.150732	C -0.387279 1.945397 -4.506083
C -2.554168 3.230831 -4.693058	C 2.230866 -4.813177 -1.470442	C 1.703931 2.036792 -4.727050	C -1.623979 2.170572 -5.125233
C -3.015367 4.424616 -4.137911	C 3.216176 -4.440468 -2.385507	C 2.966420 2.625214 -4.652657	C -2.358370 3.323684 -4.843909
C -2.303196 5.033616 -3.099375	C 4.071157 -3.371335 -2.096930	C 4.011080 1.963569 -3.997383	C -1.865185 4.264999 -3.934422
C -1.132752 4.437389 -2.625469	C 3.927326 -2.680545 -0.892801	C 3.778882 0.718059 -3.412975	C -0.641803 4.037499 -3.302725
C -0.676975 3.232966 -3.172150	C 2.937012 -3.052629 0.022870	C 2.513154 0.125952 -3.488306	C 0.097493 2.885879 -3.590087
C -0.948986 1.301312 -4.815646	C 0.977067 -4.532663 0.696014	C 0.093304 0.155663 -4.280285	C 0.400009 0.696266 -4.863259
O 2.337861 1.957496 -2.157163	O 2.861238 -1.784894 3.478989	O 1.907050 -2.882565 -1.988555	O 2.892666 2.192206 -1.844151
C 3.398051 3.996752 -1.514476	C 5.142069 -2.437512 3.598355	C 3.992911 -3.376804 -2.969979	C 3.413811 4.502722 -1.779643
C 2.814278 5.154222 -2.042091	C 5.279645 -3.330738 2.527625	C 4.240563 -3.074125 -4.313109	C 2.598356 5.372298 -2.511269
C 2.674515 6.298619 -1.252073	C 6.457105 -3.369474 1.781422	C 5.434718 -2.455272 -4.691580	C 2.186329 6.588348 -1.959196
C 3.123037 6.293629 0.072019	C 7.517695 -2.516965 2.106249	C 6.390271 -2.127322 -3.726431	C 2.594497 6.944231 -0.671092

C 3.696002 5.136413 0.608932	C 7.392225 -1.627903 3.176858	C 6.149653 -2.425567 -2.381449	C 3.402501 6.075048 0.068973
C 3.824950 3.992892 -0.179880	C 6.207080 -1.587914 3.917676	C 4.957192 -3.047776 -2.008299	C 3.803731 4.857824 -0.481206
C 3.530637 2.744075 -2.339241	C 3.837455 -2.379519 4.348554	C 2.677968 -3.966113 -2.532584	C 3.825823 3.168726 -2.337849
O 4.632416 -0.332946 -1.593893	O 1.773788 -0.069682 5.829495	O 0.770227 -5.142386 -0.095750	O 5.655866 0.419758 -1.378149
C 5.810164 0.483744 0.392314	C 2.902091 2.018572 5.180569	C 2.675215 -5.099279 1.455325	C 6.786942 1.889767 0.228227
C 5.174080 0.663767 1.626631	C 2.505071 3.168699 4.490730	C 2.875477 -4.056463 2.368572	C 6.374768 2.138429 1.542426
C 5.465118 1.776851 2.421257	C 3.353450 3.763090 3.549766	C 4.149934 -3.517943 2.568472	C 6.442223 3.428124 2.080084
C 6.398396 2.720528 1.986284	C 4.602531 3.199811 3.281487	C 5.241223 -4.025319 1.859970	C 6.926254 4.482695 1.303952
C 7.033128 2.552928 0.750372	C 5.008190 2.050040 3.969374	C 5.053853 -5.076846 0.955923	C 7.336500 4.244889 -0.012774
C 6.737547 1.442417 -0.040545	C 4.164279 1.469483 4.914551	C 3.778135 -5.603676 0.752003	C 7.264673 2.957969 -0.545360
C 5.479060 -0.701153 -0.489226	C 1.973081 1.318625 6.153610	C 1.288540 -5.629779 1.160032	C 6.687584 0.505754 -0.378369
O -0.982620 0.893607 0.807655	O 1.262187 1.074100 0.290301	O 1.549996 0.417310 1.270504	O -0.361906 1.206583 0.995260
C -0.594339 3.301947 1.135712	C 3.734101 0.915370 0.197273	C 3.974891 0.097615 1.245782	C -0.150034 3.595034 1.453731
C -1.843600 3.480057 1.738846	C 3.791996 1.262807 -1.156721	C 4.453363 0.946672 2.253253	C -1.156013 3.778840 2.413753
C -2.367143 4.761509 1.915174	C 4.971597 1.084891 -1.881457	C 5.696452 1.566166 2.122838	C -1.840909 4.990211 2.496310
C -1.646848 5.881370 1.490073	C 6.110576 0.566460 -1.258026	C 6.472185 1.348790 0.977611	C -1.530044 6.032350 1.613852
C -0.397141 5.708841 0.887847	C 6.058033 0.213953 0.093511	C 6.000182 0.506958 -0.032089	C -0.535939 5.853363 0.650181
C 0.122057 4.424852 0.708317	C 4.871218 0.376759 0.812283	C 4.757286 -0.115944 0.105913	C 0.151601 4.638098 0.571522
C 0.000496 1.919316 0.973816	C 2.487306 1.157713 1.020049	C 2.614381 -0.545786 1.364643	C 0.552256 2.260897 1.349207
O -3.888727 0.505307 -1.393297	O -0.021066 -1.143852 -2.292579	O 1.212061 3.181044 -1.082995	O -2.993196 1.636243 -1.480010
C -5.106432 2.558903 -0.777967	C 1.294466 -0.357919 -4.207765	C 2.981828 4.645043 -0.241673	C -4.627948 3.017840 -0.278062
C -6.209994 1.916849 -0.198862	C 0.196233 0.175608 -4.897168	C 2.189928 5.757032 0.077612	C -5.607760 2.015884 -0.259589
C -7.017782 2.586882 0.719080	C 0.382193 1.165270 -5.862395	C 2.551305 6.604017 1.125143	C -6.641903 2.054008 0.676462
C -6.732578 3.911962 1.067808	C 1.670071 1.630646 -6.152827	C 3.711593 6.348409 1.865827	C -6.715477 3.103075 1.599370
C -5.633361 4.556351 0.497429	C 2.767809 1.104559 -5.467684	C 4.506780 5.244738 1.550967	C -5.748196 4.110396 1.579419
C -4.820838 3.878715 -0.416545	C 2.577160 0.119675 -4.494083	C 4.139381 4.396422 0.502512	C -4.707167 4.062449 0.648086
C -4.248994 1.834073 -1.791759	C 1.088992 -1.423634 -3.152155	C 2.541063 3.681821 -1.319675	C -3.492316 2.963790 -1.279913
O -2.685364 -1.389830 1.462176	O -1.414299 1.869356 -0.805639	O -0.471235 2.486303 2.056700	O -2.593899 -0.991838 0.990917
C -3.723657 -0.011131 3.216174	C 0.212036 3.438052 -1.786083	C 0.288996 2.843388 4.279121	C -3.150870 -0.769314 3.318742
C -2.675041 0.395003 4.055158	C 0.748886 3.913453 -0.582580	C 0.995965 1.632921 4.335771	C -1.998934 -0.036482 3.636867
C -2.719624 1.630945 4.700607	C 2.022659 4.477825 -0.550077	C 1.255534 1.024570 5.563679	C -1.531291 0.006011 4.950416
C -3.815184 2.480915 4.511745	C 2.776350 4.579564 -1.726013	C 0.815351 1.621220 6.751077	C -2.205450 -0.686404 5.962030

C -4.857855 2.090340 3.669405	C 2.249367 4.106094 -2.928654	C 0.111066 2.826020 6.699274	C -3.352472 -1.419791 5.650312
C -4.810425 0.849191 3.029146	C 0.973019 3.534636 -2.954897	C -0.154373 3.432099 5.466490	C -3.820648 -1.460284 4.333464
C -3.656860 -1.350438 2.512862	C -1.182413 2.852968 -1.822831	C 0.032080 3.490304 2.940472	C -3.675696 -0.773669 1.903073
H -4.187612 -1.460800 0.031591	H -1.917475 0.386089 -2.161265	H -0.595272 3.841202 0.479324	H -3.851992 -0.504457 -0.592797
H -3.548160 0.946077 0.615804	H 0.594386 0.836449 -2.114084	H 1.767219 2.895705 0.903443	H -2.788347 1.526335 0.590548
H -1.696200 1.897198 -0.862992	H 1.828460 -0.749070 -0.532278	H 2.281686 0.911071 -0.616629	H -0.715322 2.321406 -0.720299
H -2.045854 -2.893569 -0.243813	H -2.845178 0.243903 0.238425	H -2.297568 1.896733 0.624518	H -2.088099 -2.309172 -0.988541
H 3.126547 0.413129 -3.304075	H 1.433216 -2.418727 4.853270	H 0.136551 -3.944720 -2.247707	H 3.860754 0.713052 -2.929572
H 3.206354 0.497801 -0.324960	H 1.672635 0.420806 3.828802	H 1.306345 -3.218637 0.446094	H 4.282115 1.250702 -0.066930
H 3.163332 -2.307313 -1.119571	H -0.767417 -0.326156 5.430144	H -1.547209 -4.179467 0.367960	H 4.589726 -1.646865 -0.379872
H -3.664641 -3.062594 -1.978219	H -3.333413 -1.500001 -1.309666	H -2.472460 3.313156 -1.245105	H -2.331978 -0.064120 -3.067448
H -3.329011 -1.435771 -2.612454	H -1.730099 -2.239373 -1.158206	H -1.060314 2.623665 -2.078285	H 0.757645 -1.144594 -3.153283
H -0.240771 -0.940441 -3.928683	H -0.804541 -3.341266 1.992763	H -1.552750 -0.608627 -2.557314	H 2.453019 -1.263096 -3.646218
H 1.408117 -0.806167 -4.557308	H -0.161915 -3.888563 3.550407	H -1.559494 -2.275519 -3.156722	H 4.182390 -0.329540 1.715158
H 1.103398 -0.637810 0.415520	H -0.466709 1.914776 4.333245	H -0.417610 -3.296109 2.446325	H 2.524687 0.002079 1.155423
H 1.057616 -2.394764 0.242078	H -0.333223 1.096294 2.754216	H -0.267933 -1.710713 1.641211	H -2.166143 -3.516798 -4.432501
H -0.707634 -3.774123 -1.699836	H -3.259888 -1.546335 2.204679	H -4.429233 2.267936 -2.583306	H -0.274784 -4.889522 -4.006619
H -1.901615 -4.793605 -2.519128	H -4.427617 -1.183906 0.915164	H -3.038387 2.384119 -3.696957	H 0.332835 -3.381215 -4.734754
H -0.606795 -4.723116 -4.597810	H -4.417568 -4.044375 1.705662	H -4.671259 0.756666 -4.541868	H 2.714014 -2.933966 3.432541
H 0.360977 -3.354455 -4.001447	H -4.946708 -2.916720 2.973184	H -3.115982 -0.019055 -4.160539	H 3.381960 -1.288129 3.564358
H 2.898565 -1.627779 3.338935	H -2.550864 3.045866 3.249714	H -3.349684 -2.068862 3.728916	H 1.072125 -1.195438 4.365112
H 1.963247 -0.251610 2.697357	H -2.111768 2.053551 1.838170	H -1.607005 -2.237184 4.054037	H 1.070927 -0.430780 2.771112
H -0.055470 -1.540603 2.671430	H -4.288358 0.822326 2.034761	H -2.199120 0.043212 4.476596	H 2.254657 -4.564425 -3.714489
H 0.836968 -3.036039 2.341524	H -4.707283 1.783523 3.461276	H -1.293009 0.068055 2.945963	H 2.638218 -3.854412 -2.135677
H 2.390086 -3.915359 -3.036694	H -6.265289 -4.691852 0.292588	H -5.509738 -1.533929 -4.344937	H 2.496762 -6.399825 -2.135346
H 1.419005 -4.098185 -1.565339	H -7.507476 -3.453602 -0.000660	H -4.013666 -2.205088 -3.644322	H 0.757216 -6.248043 -2.509465
H 3.171411 -6.272674 -2.806667	H -6.071654 -3.642102 -1.963652	H -6.099345 -3.419831 -2.933428	H 0.399985 -3.328148 4.598051
H 2.229034 -6.445569 -1.301932	H -4.602191 -3.455629 -0.975813	H -6.679023 -1.910529 -2.168497	H -1.234208 -2.871334 4.066569
H -0.138762 -3.273850 5.857436	H -4.474169 3.859702 0.013787	H -3.770669 1.731823 4.202199	H -2.794261 -4.482208 3.949160
H -1.176435 -2.333521 4.759965	H -3.386927 2.476483 0.168873	H -2.718470 2.272816 2.868303	H -5.231060 -4.563248 3.761293
H 2.521277 -4.616673 3.110025	H -5.160915 4.015050 -1.976620	H -7.436206 -1.467531 2.339436	H -6.331121 -4.660266 1.522173
H 4.815951 -3.919818 3.740786	H -4.434818 5.254049 -3.961783	H -6.865560 -3.569799 3.556867	H -1.575618 -9.204645 1.753627

H 6.571654 -3.753414 1.960024	H -3.272240 4.082020 -5.836234	H -5.469309 -5.303326 2.429020	H 0.903852 -9.235842 1.975924
H 6.014876 -4.365093 -0.379534	H -8.541990 0.591198 -4.934526	H -4.652454 -4.912084 0.101653	H 2.278214 -7.505303 0.801386
H -3.727478 -4.651623 2.066368	H -9.655872 -0.386894 -2.932781	H -4.957328 4.483408 -0.203280	H 0.647508 -4.938319 2.777993
H -4.236360 -6.154611 0.140614	H -8.286832 -1.496385 -1.168351	H -6.740596 4.907481 -1.900988	H -0.785833 -5.335390 3.743723
H -2.365287 -7.190799 -1.144821	H -5.639418 1.045684 0.164283	H -8.326498 3.092392 -2.520352	H -2.011602 1.440054 -5.832839
H -0.017443 -6.685532 -0.516893	H -6.294225 2.432258 -0.729256	H -8.122026 0.851738 -1.444100	H -3.317262 3.485778 -5.329476
H 0.123919 -4.937884 3.859935	H 1.572888 -5.650455 -1.696352	H -5.653285 1.454812 2.544066	H -2.439633 5.159683 -3.709594
H -1.445908 -4.890684 4.716878	H 3.318146 -4.985465 -3.320546	H -5.094754 3.142741 2.757778	H -0.256254 4.753783 -2.582217
H -3.108364 2.763508 -5.504999	H 4.839852 -3.077108 -2.806951	H 0.895119 2.550797 -5.243165	H 1.040210 2.698760 -3.087610
H -3.927726 4.879201 -4.515323	H 4.579723 -1.844480 -0.665516	H 3.136497 3.599236 -5.104248	H 0.713079 0.755605 -5.915852
H -2.657339 5.965179 -2.665660	H 2.825778 -2.518581 0.959324	H 4.995082 2.421494 -3.938553	H -0.236709 -0.189109 -4.764916
H -0.572432 4.905349 -1.823313	H 0.938032 -5.629604 0.768255	H 4.584605 0.197706 -2.903983	H 2.280019 5.092194 -3.511998
H 0.227437 2.765151 -2.800129	H 0.002932 -4.211205 0.298738	H 2.333871 -0.847122 -3.045628	H 1.550040 7.255501 -2.534484
H -1.032334 1.352807 -5.911300	H 4.449625 -3.983476 2.271966	H -0.189429 0.108301 -5.341988	H 2.277255 7.890736 -0.241963
H -1.631801 0.503671 -4.490814	H 6.544853 -4.058483 0.945750	H -0.658771 0.786646 -3.786989	H 3.716198 6.343429 1.074368
H 2.467403 5.156056 -3.072923	H 8.435937 -2.544675 1.525682	H 3.491804 -3.315398 -5.063939	H 4.424893 4.176992 0.093815
H 2.218445 7.192135 -1.669747	H 8.212947 -0.963395 3.433090	H 5.615499 -2.222970 -5.737738	H 3.806385 3.163768 -3.436914
H 3.017900 7.184575 0.684977	H 6.111179 -0.896383 4.749894	H 7.317037 -1.641668 -4.020082	H 4.839480 2.912023 -2.008264
H 4.038786 5.121458 1.639991	H 3.504862 -3.391824 4.622175	H 6.886052 -2.171759 -1.623771	H 6.002463 1.317044 2.150494
H 4.258761 3.088506 0.235637	H 3.933539 -1.791464 5.268990	H 4.766502 -3.274520 -0.963397	H 6.116463 3.605090 3.101580
H 3.648581 2.974309 -3.407768	H 1.520344 3.591799 4.675636	H 2.142592 -4.415809 -3.381048	H 6.979289 5.486025 1.717792
H 4.404810 2.170174 -2.012278	H 3.028106 4.650116 3.012122	H 2.823286 -4.736649 -1.767497	H 7.710418 5.063249 -0.622140
H 4.446936 -0.072334 1.953747	H 5.251321 3.640701 2.529729	H 2.027679 -3.657492 2.920262	H 7.581336 2.774485 -1.569501
H 4.961205 1.906240 3.375511	H 5.972057 1.597296 3.753975	H 4.287133 -2.698490 3.268697	H 7.607912 0.250299 -0.911130
H 6.626549 3.586328 2.602063	H 4.467248 0.569178 5.440196	H 6.231980 -3.605000 2.009597	H 6.525420 -0.248457 0.404032
H 7.755523 3.287785 0.405044	H 2.401592 1.301347 7.160478	H 5.899726 -5.477446 0.403994	H -1.401848 2.963520 3.089972
H 7.228149 1.311773 -1.002679	H 1.009513 1.844135 6.205895	H 3.630529 -6.411516 0.039145	H -2.620208 5.123099 3.241951
H 6.385311 -1.100990 -0.953784	H 2.911189 1.671851 -1.639984	H 1.305677 -6.716436 1.041400	H -2.064968 6.976299 1.677620
H 5.009951 -1.501243 0.095974	H 5.000688 1.358070 -2.933251	H 0.596757 -5.385532 1.976724	H -0.287205 6.655145 -0.039055
H -2.409434 2.611601 2.057441	H 7.028440 0.430307 -1.824094	H 3.846417 1.120465 3.139100	H 0.925689 4.502100 -0.178133
H -3.343368 4.878382 2.376365	H 6.927635 -0.211050 0.585103	H 6.060086 2.222205 2.909266	H 1.371041 2.301936 0.623582
H -2.058538 6.878570 1.621727	H 4.822093 0.071876 1.853761	H 7.439010 1.834412 0.875257	H 0.959696 1.954890 2.317899

H 0.172488 6.567128 0.542365<br>H 1.080860 4.293073 0.217621<br>H 0.720637 1.907155 0.149870<br>H 0.536115 1.632191 1.886953<br>H -6.421936 0.884065 -0.463944<br>H -7.867478 2.076826 1.165603<br>H -7.361363 4.434083 1.784033<br>H -5.397183 5.580735 0.771771<br>H -3.955093 4.374458 -0.846524<br>H -4.802930 1.685157 -2.724908<br>H -3.363514 2.428527 -2.038559<br>H -1.818708 -0.260397 4.192763<br>H -1.900395 1.933924 5.347165<br>H -3.848279 3.446203 5.009274<br>H -5.704367 2.748757 3.499638<br>H -5.620181 0.552978 2.367686<br>H -3.337111 -2.129561 3.211510<br>H -4.644990 -1.626021 2.119507<br>H -1.528196 -0.019327 -2.389669<br>O 0.334219 0.405688 -1.593431	H 2.477506 0.479562 1.874854<br>H 2.495055 2.183842 1.401244<br>H -0.804822 -0.175104 -4.658349<br>H -0.477227 1.576878 -6.385718<br>H 1.813786 2.402726 -6.903988<br>H 3.769844 1.467790 -5.680362<br>H 3.428708 -0.275608 -3.946670<br>H 0.841444 -2.387362 -3.609460<br>H 2.007751 -1.565197 -2.571814<br>H 0.171610 3.811517 0.331675<br>H 2.433227 4.829621 0.393378<br>H 3.771976 5.014293 -1.699309<br>H 2.831264 4.163359 -3.844852<br>H 0.573087 3.146830 -3.888910<br>H -1.925567 3.637975 -1.639315<br>H -1.395865 2.433389 -2.812637<br>H -0.034895 -2.038504 0.289813<br>O 0.846991 -1.109229 1.910560<br>H -6.072620 0.449495 -5.169583<br>H -2.920418 1.611114 -5.688094	H 6.594148 0.332548 -0.925503<br>H 4.391096 -0.775907 -0.674301<br>H 2.490597 -1.322375 0.604899<br>H 2.480011 -1.000155 2.349897<br>H 1.277825 5.942053 -0.484641<br>H 1.928184 7.460603 1.368964<br>H 3.989177 7.005505 2.685802<br>H 5.403699 5.032505 2.127180<br>H 4.743903 3.522576 0.274458<br>H 2.478248 4.165342 -2.297836<br>H 3.255034 2.853272 -1.404585<br>H 1.320159 1.172222 3.408376<br>H 1.801054 0.084527 5.594104<br>H 1.016362 1.147090 7.708180<br>H -0.240810 3.291994 7.616046<br>H -0.712118 4.365277 5.429543<br>H -0.694231 4.311330 3.027262<br>H 0.963026 3.919323 2.541487<br>K -3.405732 -0.670450 0.028164<br>H 0.149116 0.742720 -1.761223<br>O 0.515693 -1.032960 -0.785292	H -5.544351 1.196822 -0.970438<br>H -7.389911 1.265314 0.687182<br>H -7.518960 3.130597 2.330703<br>H -5.791668 4.925063 2.297800<br>H -3.941069 4.832791 0.651639<br>H -3.824251 3.283985 -2.271466<br>H -2.681903 3.639779 -0.974674<br>H -1.463977 0.475958 2.844205<br>H -0.633712 0.573353 5.183400<br>H -1.836446 -0.657078 6.983976<br>H -3.879726 -1.967178 6.427337<br>H -4.705486 -2.042242 4.091328<br>H -4.436402 -1.555763 1.768176<br>H -4.159249 0.190459 1.687879<br>K 0.678385 -2.934636 0.099529<br>H -0.371648 0.619231 -2.302231<br>O 1.213274 0.429491 -1.000036<br>H -2.680822 -7.422457 0.425848<br>H -4.900480 -4.736874 -0.528025<br>H -3.357789 -1.495565 -2.890040<br>H -1.917305 -3.583541 -2.675422

7. References

1. Lewandowski, B.; Listkowski, A.; Petrova, K.; Jarosz, S., Functionalisation of terminal positions of sucrose - Part II: Preparation of 1,2,3,3’,4,4’-hexa-O-benzylsucrose and 6,6’-Bis-O-(2-hydroxyethyl)-1,2,3,3’,4,4’-hexa-O-benzylsucrose. 2011 (book chapter), in Carbohydrate Chemistry: Proven Synthetic Methods, vol.I, CRC Press, 413-430.

2. Yang, C.-T.; Fu, Y.; Huang, Y.-B.; Yi, J.; Guo, Q.-X.; Liu, L., Room-Temperature Copper-Catalyzed Carbon–Nitrogen Coupling of Aryl Iodides and Bromides Promoted by Organic Ionic Bases. *Angew. Chem. Int. Ed.* **2009,** *48* (40), 7398-7401.

3. (a) Dąbrowa, K.; Niedbała, P.; Jurczak, J., Engineering Light-Mediated Bistable Azobenzene Switches Bearing Urea d-Aminoglucose Units for Chiral Discrimination of Carboxylates. *J. Org. Chem.* **2016,** *81* (9), 3576-3584; (b) Dąbrowa, K.; Jurczak, J., Tetra-(meta-butylcarbamoyl)azobenzene: A Rationally Designed Photoswitch with Binding Affinity for Oxoanions in a Long-Lived Z-State. *Org. Lett.* **2017,** *19* (6), 1378-1381.

4. (a) Frassineti, C.; Ghelli, S.; Gans, P.; Sabatini, A.; Moruzzi, M. S.; Vacca, A., Nuclear Magnetic Resonance as a Tool for Determining Protonation Constants of Natural Polyprotic Bases in Solution. *Anal. Biochem.* **1995,** *231* (2), 374-382; (b) Frassineti, C.; Alderighi, L.; Gans, P.; Sabatini, A.; Vacca, A.; Ghelli, S., Determination of protonation constants of some fluorinated polyamines by means of 13C NMR data processed by the new computer program HypNMR2000. Protonation sequence in polyamines. *Anal. Bioanal. Chem.* **2003,** *376* (7), 1041-1052; (c) Rodríguez-Barrientos, D.; Rojas-Hernández, A.; Gutiérrez, A.; Moya-Hernández, R.; Gómez-Balderas, R.; Ramírez-Silva, M. T., Determination of pKa values of tenoxicam from 1H NMR chemical shifts and of oxicams from electrophoretic mobilities (CZE) with the aid of programs SQUAD and HYPNMR. *Talanta* **2009,** *80* (2), 754-762.

5. (a) Lowe, A. J.; Pfeffer, F. M.; Thordarson, P., Determining binding constants from 1H NMR titration data using global and local methods: a case study using [n]polynorbornane-based anion hosts. *Supramol. Chem.* **2012,** *24* (8), 585-594; (b) Thordarson, P., Determining association constants from titration experiments in supramolecular chemistry. *Chem. Soc. Rev.* **2011,** *40* (3), 1305-1323.
